# ATF7 drives diabetic wound healing via NOTCH1 repression and N1ICD-dependent macrophage polarization control

**DOI:** 10.1172/jci.insight.201178

**Published:** 2026-08-24

**Authors:** Pengcheng Xu, Yuan Xue, Linlin Feng, Jingwen Kuang, Xiaochen Hu, Huiyi Tang, Biao Cheng, Limin Wei

**Affiliations:** 1 Department of Breast Surgery, and; 2 Department of Thyroid and Head and Neck Oncology Surgery, The First Affiliated Hospital of Henan University of Science and Technology, Luoyang, China.; 3 The First Affiliated Hospital of Henan University of Science and Technology, Luoyang, China.; 4 Henan Key Laboratory of Cancer Epigenetics, Cancer Hospital, The First Affiliated Hospital of Henan University of Science and Technology, Luoyang, China.; 5 Department of Sports Medicine, Guangzhou Sport University, Guangzhou, China.; 6 Department of Burn and Plastic Surgery, General Hospital of Southern Theater Command, PLA, Guangzhou, China.

**Keywords:** Dermatology, Inflammation, Diabetes, Macrophages, Molecular biology

## Abstract

Chronic, non-healing wounds are a severe diabetic complication. The underlying mechanisms are not fully understood, and the role of ATF7 in this context has not been well characterized. In our study, we utilized db/db diabetic mice and AAV-mediated keratinocyte-specific *Atf7* overexpression in vivo. HaCaT keratinocyte/THP-1 macrophage cocultures under high glucose were used in vitro. Our results showed that ATF7 was upregulated in diabetic wounds. Keratinocyte-specific *Atf7* overexpression accelerated diabetic wound closure, enhanced re-epithelialization, granulation tissue formation, and keratinocyte proliferation, while suppressing macrophage M1 polarization and inflammation. Multiomics screening identified NOTCH1 as a key ATF7 target. ATF7 transcriptionally repressed NOTCH1 by recruiting Suv39h1, increasing H3K9me3 at the *NOTCH1* promoter. This reduced NOTCH1 protein and its active intracellular domain (N1ICD) within keratinocyte-derived exosomes. *ATF7*-overexpressing keratinocyte exosomes carried less N1ICD, leading to decreased N1ICD transfer to macrophages and subsequent inhibition of M1 polarization. Notably, local injection of exosomes from *ATF7*-overexpressing keratinocytes accelerated wound healing in db/db mice. In summary, ATF7 promotes diabetic wound healing by repressing NOTCH1 transcription via H3K9me3, thereby reducing exosomal N1ICD secretion from keratinocytes and inhibiting macrophage M1 polarization. This identifies the ATF7/NOTCH1/exosome axis as a therapeutic target.

## Abstract 2

ATF7 in keratinocytes promotes diabetic wound healing by reducing inflammation through exosomes.

## Introduction

Chronic non-healing wounds represent a common complication of diabetes, severely impacting patients’ quality of life and safety, and have become a leading cause of disability among individuals with diabetes (1). The mechanisms underlying diabetic wound healing remain incompletely understood, and there is a lack of ideal therapeutic approaches and drugs (2). Therefore, exploring the mechanisms of diabetic wound healing and identifying molecular targets is of importance.

Factors influencing wound healing in diabetic patients include angiogenesis, collagen deposition, macrophage function, cell proliferation and migration, growth factor production, and granulation tissue formation (3). Wound healing is a complex, dynamic process primarily involving 3 overlapping phases: inflammation, proliferation, and tissue remodeling (4). Keratinocytes, as key cellular components of the skin epidermis, play a crucial role in wound repair. Studies have shown that processes such as keratinocyte proliferation, migration, and differentiation directly impact re-epithelialization and wound closure (5). Furthermore, evidence indicates that keratinocytes influence skin wound repair by communicating with macrophages, regulating macrophage recruitment and their ability to polarize into distinct phenotypes (6, 7).

Activating transcription factor 7 (ATF7), a member of the ATF/CREB superfamily of transcription factors, binds to the cAMP response element (CRE) (8). Research suggests ATF7 functions as a stress-response modulator involved in diverse biological processes, including innate immune responses, adipocyte differentiation, and telomerase regulation (9). Through bioinformatics analysis of the NCBI Gene Expression Omnibus (GEO) GSE182906 dataset, we found that *Atf7* expression is upregulated in wound tissues of db/db diabetic mice on day 14 after skin injury compared with wild-type mice. However, the specific role of ATF7 in diabetic wound repair remains unclear. Previous studies demonstrated that ATF7 suppresses cellular senescence and inflammatory responses by inhibiting the NF-κB signaling pathway (10). Additionally, knockdown of ATF7 has been shown to inhibit proliferation and cell cycle progression in HeLa S3 cells (11). Moreover, Meijer et al. demonstrated that ATF2 and ATF7 are critical protective factors for intestinal epithelial injury repair; simultaneous knockout of ATF2 and ATF7 inhibited epithelial cell proliferation and migration while promoting TNF-α expression (12). This collective evidence suggests ATF7 possesses functions that promote cell proliferation and suppress inflammation, suggesting that the compensatory upregulation of ATF7 in diabetic wound tissue may promote wound repair.

Thus, in our study, we investigated whether ATF7 promotes the healing of diabetic wounds, highlighting the potential role of ATF7 as a therapeutic target for chronic non-healing wounds.

## Results

### Atf7 expression is upregulated in the wound tissues of diabetic mice.

**Figure 1:**
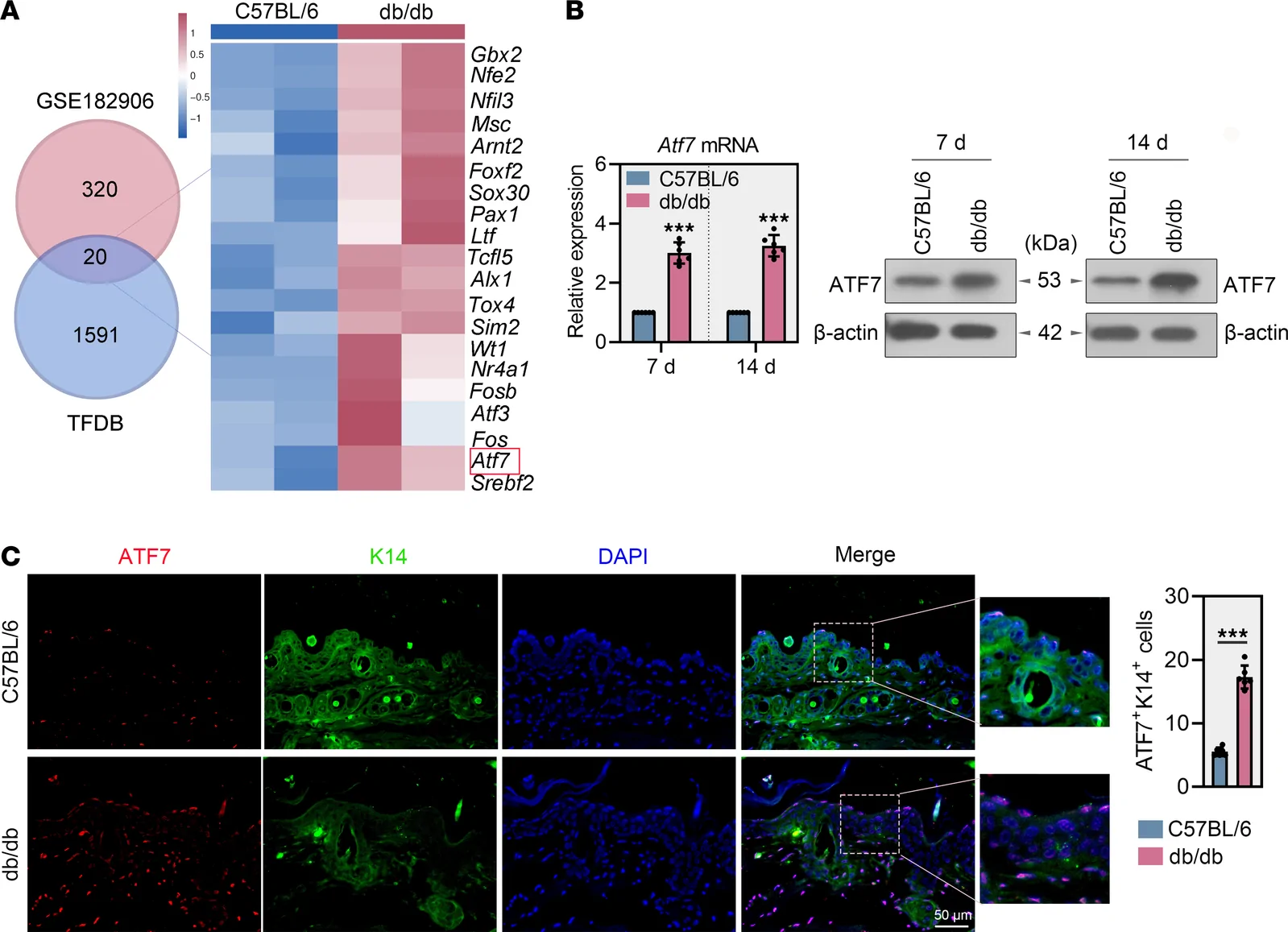
*Atf7* expression is upregulated in wound tissues of diabetic mice. (**A**) Differentially upregulated genes (|logFC| ≥ 1.5, *P* < 0.05) were screened from the GSE182906 dataset (day 14) and intersected with the *Mus* *musculus* transcription factor (TF) dataset from AnimalTFDB. A cluster heatmap displays the expression profiles of the 20 identified TFs. (**B**) Full-thickness excisional skin wounds were created on the dorsum of 10-week-old male db/db and C57BL/6 mice. *Atf7* mRNA and ATF7 protein expression in wound tissues was analyzed by qRT-PCR and Western blotting on days 7 and 14 after wounding. (**C**) Representative immunofluorescence images showing colocalization of ATF7 and the keratinocyte marker keratin 14 (K14) in wound tissues and quantitative analysis for double-positive cells. Nuclei were counterstained with DAPI. Scale bar: 50 μm (original magnification: ×400). Results are expressed as mean ± SD. ***P < 0.001 by 2-tailed, unpaired Student’s *t* test. Each experimental group consisted of 6 animals (*n* = 6).

Differentially upregulated genes were screened from the GSE182906 (day 14) dataset. These genes were then intersected with the transcription factor (TF) dataset from the AnimalTFDB website, yielding 20 differentially upregulated TFs (Figure 1A). ATF7 was selected for further investigation based on its established roles in inflammation and proliferation. A full-thickness skin wound model was established using db/db and C57BL/6 mice. Quantitative real-time PCR (qRT-PCR) and Western blot analysis revealed that ATF7 expression was upregulated in the wound tissues of db/db mice compared with C57BL/6 control mice (Figure 1B). Subsequently, immunofluorescence (IF) costaining for ATF7 and the keratinocyte marker keratin 14 (K14) was performed on wound tissues. In C57BL/6 mice, ATF7 and K14 exhibited colocalization. Within the wound tissues of db/db mice, K14 expression was decreased while ATF7 expression was elevated (Figure 1C).

### Atf7 overexpression promotes diabetic wound healing.

**Figure 2:**
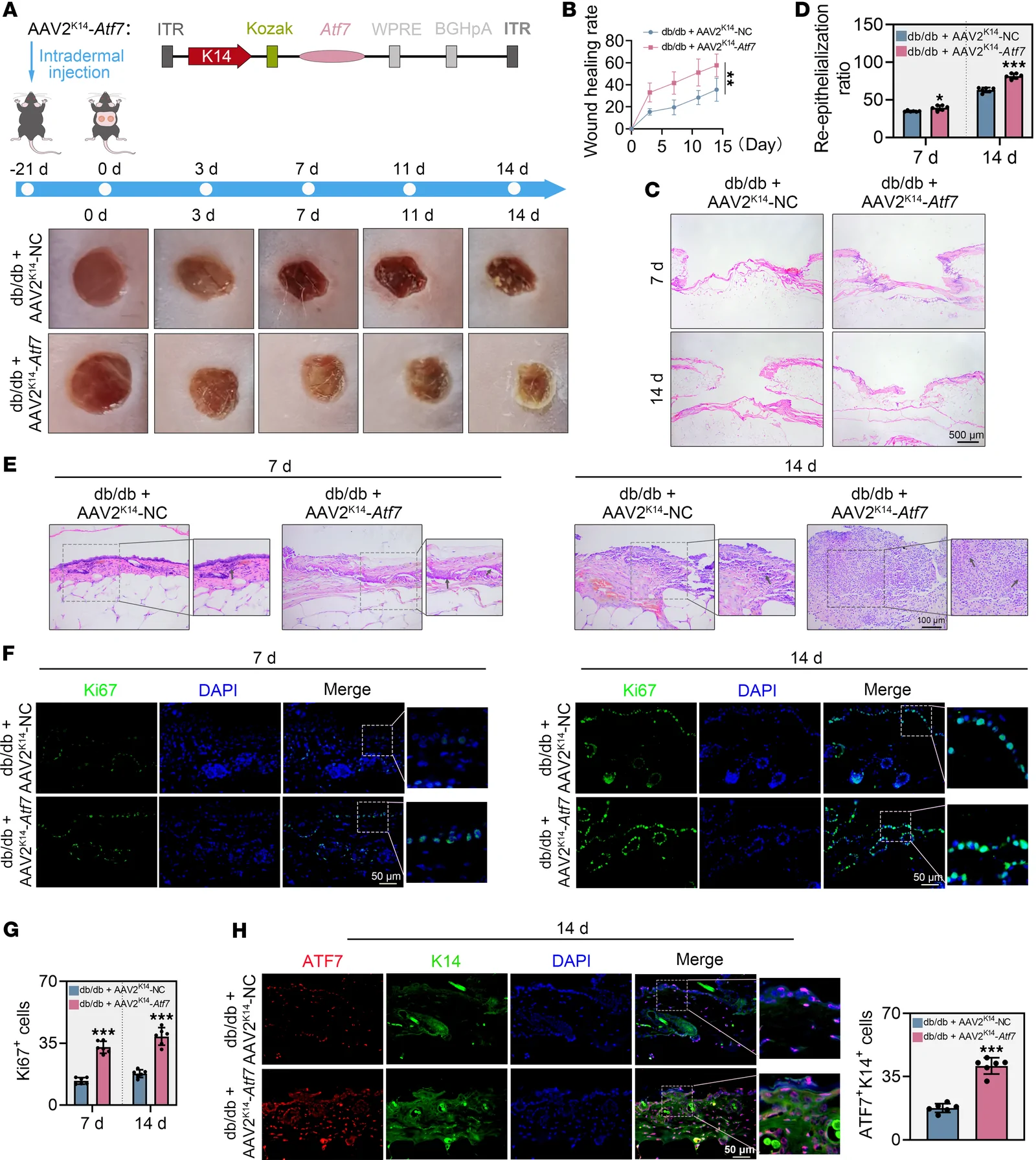
*Atf7* overexpression promotes diabetic wound healing. (**A**) Schematic of the AAV2 vector construct. Keratinocyte-specific overexpression of *Atf7* was achieved using an AAV2 serotype vector driven by the *K14* promoter (AAV2^K14^-*Atf7*). Db/db mice (10-week-old males) received intradermal injections of AAV2^K14^-*Atf7* or control vector at the wound margin site. Three weeks after injection, full-thickness excisional skin wounds were created on the dorsum. Representative wound photographs at indicated days after wounding (days 0, 3, 7, 11, and 14). (**B**) Quantitative analysis of wound closure rate. (**C**) Representative H&E-stained sections of wound tissues. Scale bar: 500 μm (original magnification: ×40). (**D**) Quantification of re-epithelialization ratio from H&E-stained sections. (**E**) Representative H&E-stained sections showing granulation tissue formation. Arrows highlight granulation tissue. Scale bar: 100 μm (original magnification: ×200). (**F**) Representative immunofluorescence images showing Ki67 expression (proliferation marker) in wound tissues. Nuclei counterstained with DAPI. Scale bar: 50 μm (original magnification: ×400). (**G**) The quantitative analysis of Ki67^+^ cells. (**H**) Representative immunofluorescence images showing colocalization of ATF7 and K14 in wound tissues and quantitative analysis for double-positive cells. Nuclei counterstained with DAPI. Scale bar: 50 μm (original magnification: ×400. Results are expressed as mean ± SD. *P < 0.05; **P < 0.01; ***P < 0.001 by 2-way ANOVA with Bonferroni’s post hoc test (**B**) or 2-tailed, unpaired Student’s *t* test (**D**, **G**, and **H**). Each experimental group consisted of 6 animals (*n* = 6).

To investigate the functional role of ATF7 in vivo, we constructed an adeno-associated virus (AAV) carrying *Atf7* under the control of the keratinocyte-specific *K14* promoter. This vector was administered to mice to achieve keratinocyte-specific overexpression of ATF7 (Supplemental Figure 1B; supplemental material available online with this article; https://doi.org/10.1172/jci.insight.201178DS1). Subsequently, a full-thickness skin wound model was established in these mice. We found that AAV-mediated *Atf7* overexpression accelerated wound closure rates in db/db mice compared with controls (Figure 2, A and B). Histological analysis of wound tissues on days 7 and 14 after wounding revealed that *Atf7* overexpression resulted in a reduced distance between the wound edges in diabetic mice (Figure 2, C and D). This finding indicates enhanced re-epithelialization and wound contraction in the diabetic mice following *Atf7* overexpression. Furthermore, *Atf7*-overexpressing wounds exhibited increased granulation tissue formation (Figure 2E). To assess cellular proliferation, we evaluated the expression of the proliferation marker Ki67. IF analysis revealed an increase in Ki67-positive cells within the wounds of *Atf7*-overexpressing db/db mice (Figure 2, F and G). Additionally, increased colocalization of ATF7 and K14 in wound tissues was observed following *Atf7* overexpression (Figure 2H). Collectively, these results suggest that *Atf7* overexpression promotes wound healing in diabetic mice.

### Atf7 overexpression inhibits macrophage M1 polarization in diabetic wounds.

**Figure 3:**
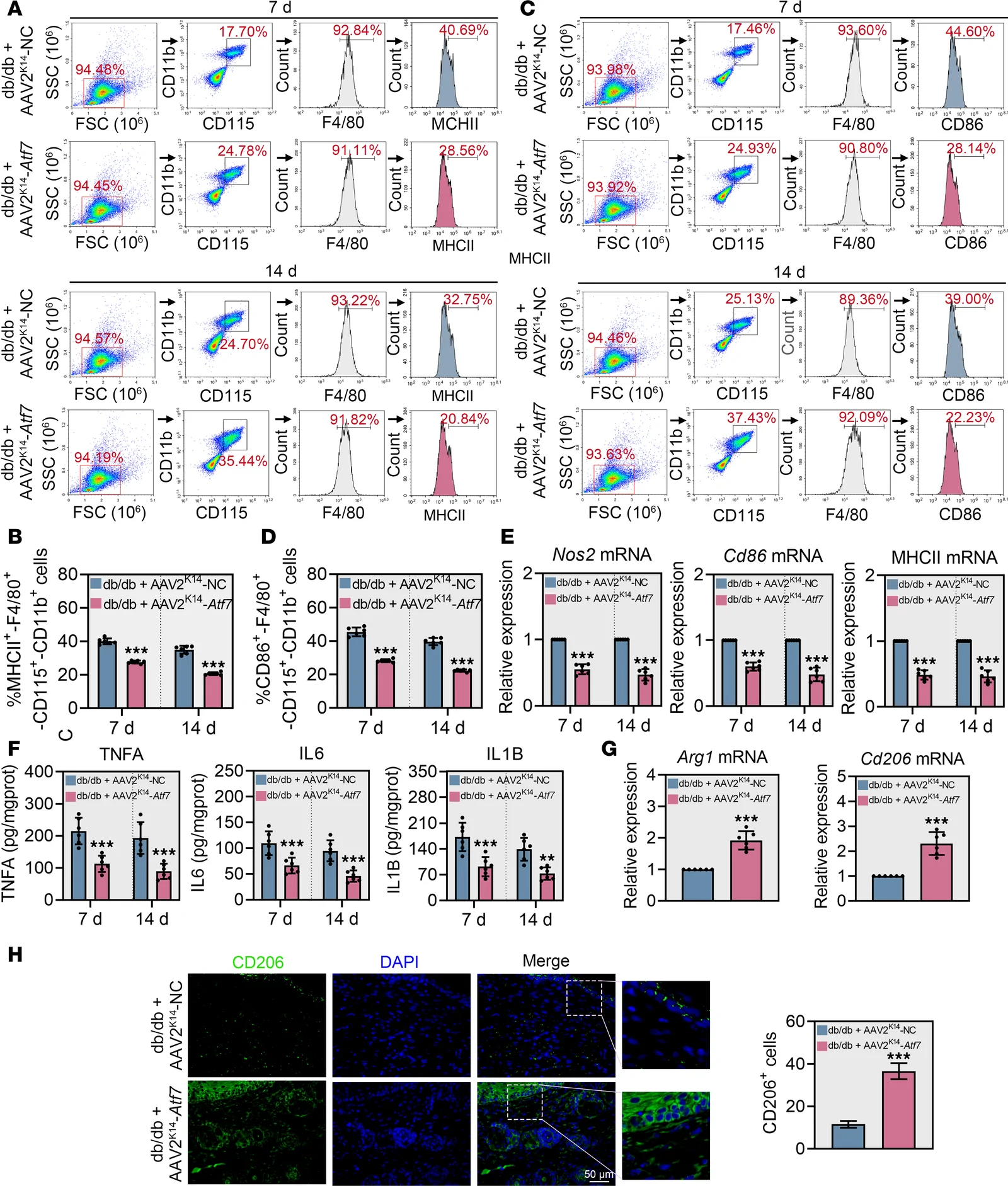
*Atf7* overexpression inhibits macrophage M1 polarization in diabetic wounds. Single-cell suspensions were prepared from skin wound tissues of db/db mice. (**A**) Schematic of the gating strategy for identifying macrophages for the detection of M1 macrophages (MHCII^+^F4/80^+^CD115^+^CD11b^+^). (**B**) Quantitative analysis of the percentage of M1 macrophages (MHCII^+^F4/80^+^CD115^+^CD11b^+^). (**C**) Schematic of the gating strategy for the detection of M1 macrophages (CD86^+^F4/80^+^CD115^+^CD11b^+^). (**D**) Quantitative analysis of the percentage of M1 macrophages (CD86^+^F4/80^+^CD115^+^CD11b^+^). (**E**) mRNA expression levels of M1 macrophage markers (*Nos2*, *Cd86*, and MHCII) in wound tissues were measured by qRT-PCR. (**F**) Levels of proinflammatory cytokines (TNF-α, IL-6, and IL-1B) in wound tissues were measured by ELISA. (**G**) Levels of M2 macrophage markers (*Arg1* and *Cd206*) were measured by qRT-PCR. (**H**) Representative immunofluorescence images showing CD206 expression in wound tissues and quantitative analysis for CD206^+^ cells. Nuclei counterstained with DAPI. Scale bar: 50 μm (original magnification: ×400) (**E**, **F**, and **H**). Results are expressed as mean ± SD. **P < 0.01, ***P < 0.001 by 2-tailed, unpaired Student’s *t* test. Each experimental group consisted of 6 animals (*n* = 6).

To assess macrophage polarization states within the wound microenvironment, single-cell suspensions were prepared from skin wound tissues and analyzed by flow cytometry. The percentages of M1 macrophages, defined as MHCII^+^F4/80^+^CD115^+^CD11b^+^ and CD86^+^F4/80^+^CD115^+^CD11b^+^ cells, were quantified. Flow cytometric analysis revealed a reduction in the percentage of both MHCII^+^ (Figure 3, A and B) and CD86^+^ (Figure 3, C and D) M1 macrophages in wound tissues following *Atf7* overexpression. Consistent with this finding, the expression levels of M1 macrophage markers (*Nos2*, *Cd86*, and MHCII) were downregulated in *Atf7*-overexpressing wound tissues (Figure 3E). Furthermore, the expression of proinflammatory cytokines TNF-α, IL-6, and IL-1B was also decreased in wounds overexpressing *Atf7* (Figure 3F). To determine whether ATF7 overexpression promotes M2 polarization in diabetic wounds, we analyzed M2 macrophage markers in wound tissues. qPCR analysis showed that the mRNA levels of M2-associated genes *Arg1* and *Cd206* were upregulated in *Atf7*-overexpressing wound tissues (Figure 3G). IF for the M2 marker CD206 revealed an increase in CD206-positive cells within the tissue of *Atf7*-overexpressing wounds (Figure 3H). These data indicate that *Atf7* overexpression not only suppresses the proinflammatory M1 phenotype but also promotes an M2 phenotype in the diabetic wound microenvironment.

### ATF7 promotes the proliferation of keratinocytes under high-glucose conditions.

**Figure 4:**
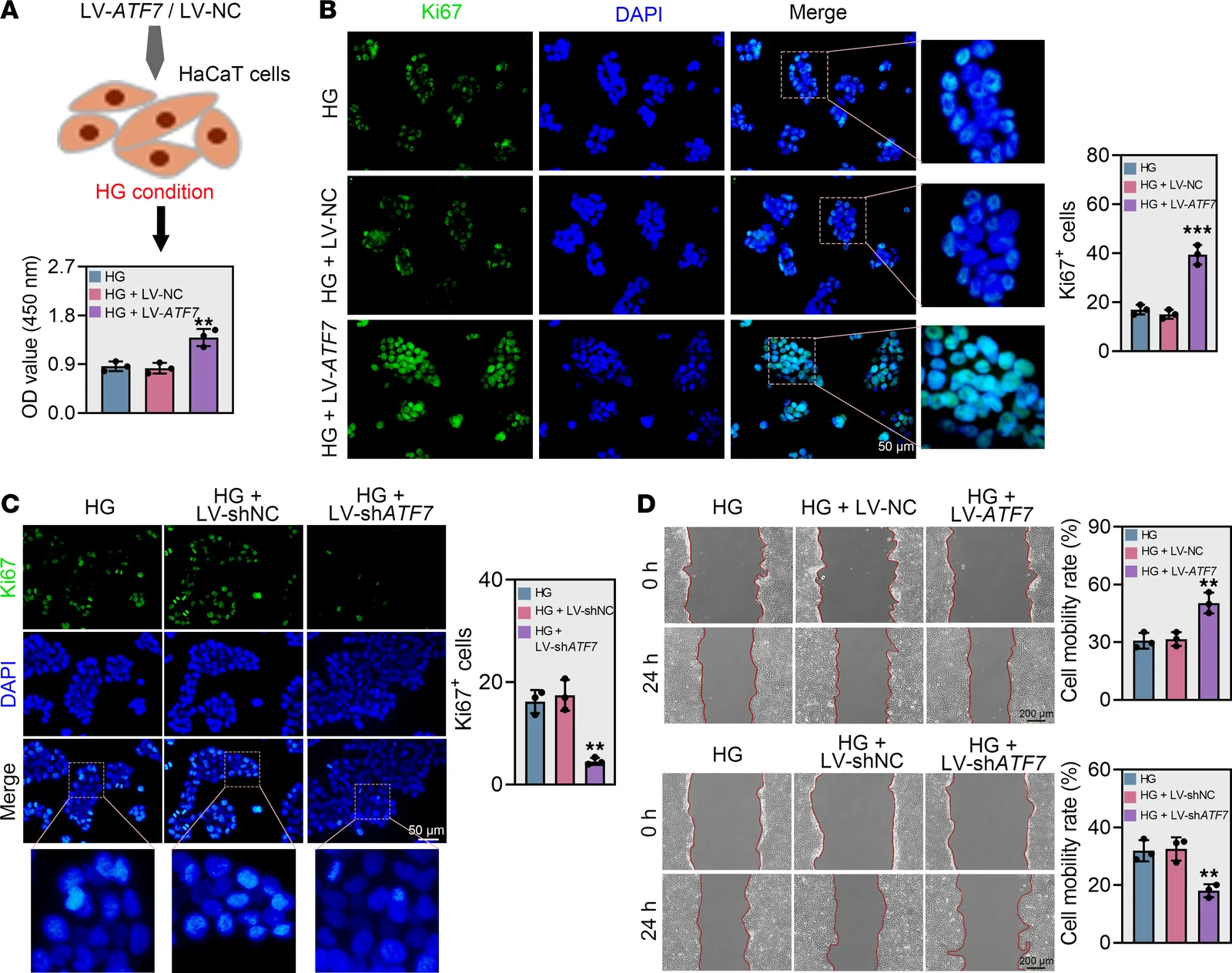
*ATF7* promotes the proliferation of keratinocytes under high-glucose conditions. HaCaT cells were infected with lentivirus expressing *ATF7* (LV-*ATF7*) or sh*ATF7* (LV-sh*ATF7*) for 48 hours, followed by culture in 25 mM high-glucose (HG) medium for 24 hours. (**A**) Cell viability was assessed by CCK-8 assay. (**B** and **C**) Representative immunofluorescence images of Ki67 expression and quantitative analysis for Ki67^+^ cells. Nuclei counterstained with DAPI. Scale bar: 50 μm (original magnification: ×400). (**D**) Cell migration ability was analyzed by the scratch wound healing assay. Left: Representative images at 0 hours and 24 hours after scratching. Scale bars: 200 μm. Right: Quantification of wound closure percentage. Results are expressed as mean ± SD. **P < 0.01; *** *P* < 0.001 by 1-way ANOVA with Bonferroni’s post hoc test. All cell experiments were performed with *n* = 3 replicates.

To investigate the effect of ATF7 on keratinocyte function in vitro, HaCaT cells were infected with lentivirus expressing human *ATF7* (LV-*ATF7*) to achieve *ATF7* overexpression (Supplemental Figure 1C) or with lentivirus expressing shRNA to knock down *ATF7* (Supplemental Figure 1D), followed by culture under high-glucose (HG) conditions. CCK-8 assay results showed that *ATF7* overexpression enhanced the viability of HaCaT cells exposed to HG (Figure 4A). IF staining for Ki67 revealed an increase in Ki67-positive cells in *ATF7*-overexpressing cells (Figure 4B), while *ATF7* knockdown decreased Ki67-positive cells (Figure 4C). Furthermore, a wound healing assay revealed that *ATF7* overexpression promoted the migration of HaCaT cells in HG medium, whereas *ATF7* knockdown impaired cell migration (Figure 4D). Collectively, these results indicate that *ATF7* ameliorates HG-induced damage to keratinocytes by promoting their proliferation and migration.

### ATF7 overexpression inhibits macrophage M1 polarization.

**Figure 5:**
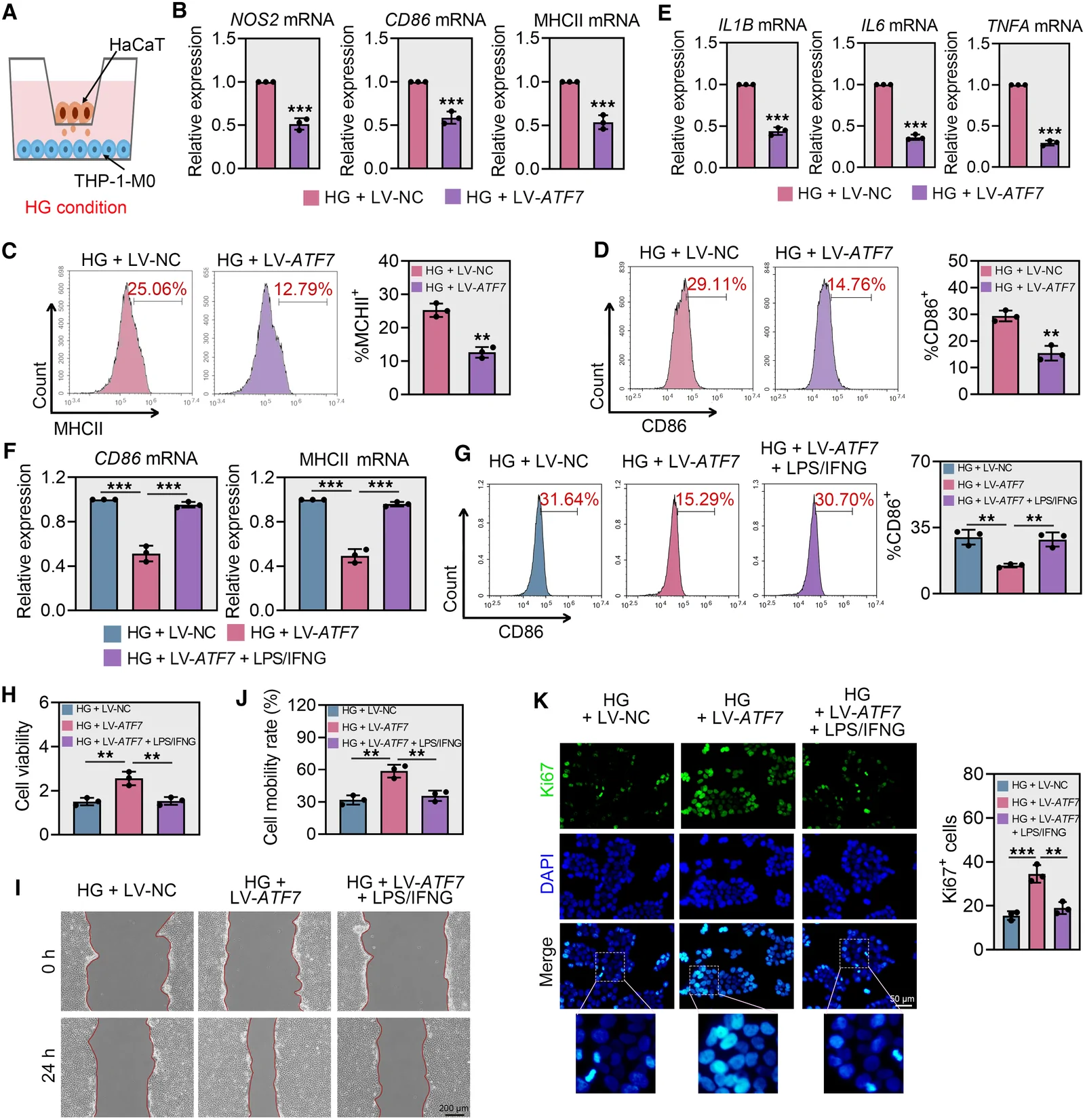
*ATF7* overexpression inhibits macrophage M1 polarization. (**A**) Experimental workflow: Human monocytic THP-1 cells were differentiated into M0 macrophages (THP-1-M0) using 20 nM PMA for 36 hours. HaCaT cells were infected with lentivirus for 48 hours, followed by coculture with THP-1-M0 macrophages in a Transwell system for 24 hours (upper chamber, HaCaT; lower chamber, macrophages). (**B**) mRNA expression of M1 markers (*NOS2*, *CD86*, and MHCII) in cocultured macrophages was measured by qRT-PCR. (**C**) Flow cytometric analysis of MHCII^+^ cells in cocultured macrophages. (**D**) Flow cytometric analysis of CD86^+^ cells in cocultured macrophages. (**E**) mRNA expression of proinflammatory cytokines (*TNFA*, *IL6*, and *IL1B*) in cocultured macrophages were measured by qRT-PCR. HaCaT cells were infected with LV-NC or LV-*ATF7* and cocultured with THP-1–derived macrophages under HG conditions. In the rescue group (LV-*ATF7* + LPS/IFN-γ), macrophages were additionally treated with LPS and IFN-γ during coculture to force M1 polarization. (**F**) mRNA expression of M1 markers (*CD86* and MHCII) in macrophages was measured by qRT-PCR. (**G**) Flow cytometric analysis of CD86^+^ cells in cocultured macrophages. (**H**) Cell viability was assessed by the CCK-8 assay. (**I** and **J**) Cell migration ability was analyzed by the scratch wound healing assay. Scale bar: 200 μm. (**K**) Representative immunofluorescence images of Ki67 expression and quantitative analysis for Ki67^+^ cells. Nuclei counterstained with DAPI. Scale bar: 50 μm (original magnification: ×400). Results are expressed as mean ± SD. **P < 0.01; ***P < 0.001 by 2-tailed, unpaired Student’s *t* test (**B**–**E**) or 1-way ANOVA with Bonferroni’s post hoc test (**F**–**H**, **J**, and **K**). All cell experiments were performed with *n* = 3 replicates.

To investigate the effect of *ATF7*-overexpressing keratinocytes on macrophage polarization, a coculture system was employed. Human monocytic THP-1 cells were differentiated into M0 macrophages (THP-1-M0) and cocultured with HaCaT cells using a Transwell system (Figure 5A). Following coculture, macrophages in the lower chamber were harvested for analysis. Notably, *ATF7* overexpression in HaCaT cells suppressed the expression of M1 macrophage markers (*NOS2*, *CD86*, MHCII) in the cocultured macrophages (Figure 5B). Furthermore, flow cytometric analysis revealed a decrease in the percentage of MHCII^+^ and CD86^+^ cells within the macrophage population (Figure 5, C and D). The expression of *TNFA*, *IL6*, and *IL1B* was downregulated in these macrophages (Figure 5E). To test whether the effects of ATF7 on keratinocytes depend on its ability to suppress M1 polarization, we established a coculture system with a rescue design. HaCaT cells were infected with negative control lentivirus (LV-NC) or LV-*ATF7*, and cocultured with THP-1 under HG conditions. In the rescue group, macrophages were treated with LPS plus IFN-γ. We then assessed both macrophage polarization and keratinocyte function. qPCR and flow cytometry exhibited that in the rescue group, the expression of *CD86* and MHCII in macrophages was restored compared with the LV-*ATF7* group (Figure 5, F and G). CCK-8, IF, and wound healing assays revealed that while *ATF7* overexpression enhanced HaCaT viability, proliferation, and migration, these beneficial effects were attenuated when macrophages were forced to remain in the M1 state (Figure 5, H–K). This in vitro rescue experiment showed that the ability of *ATF7* to suppress M1 polarization is critical for its protective effects on keratinocytes.

### ATF7-overexpressing keratinocyte-derived exosomes promote diabetic wound healing in vivo.

**Figure 6:**
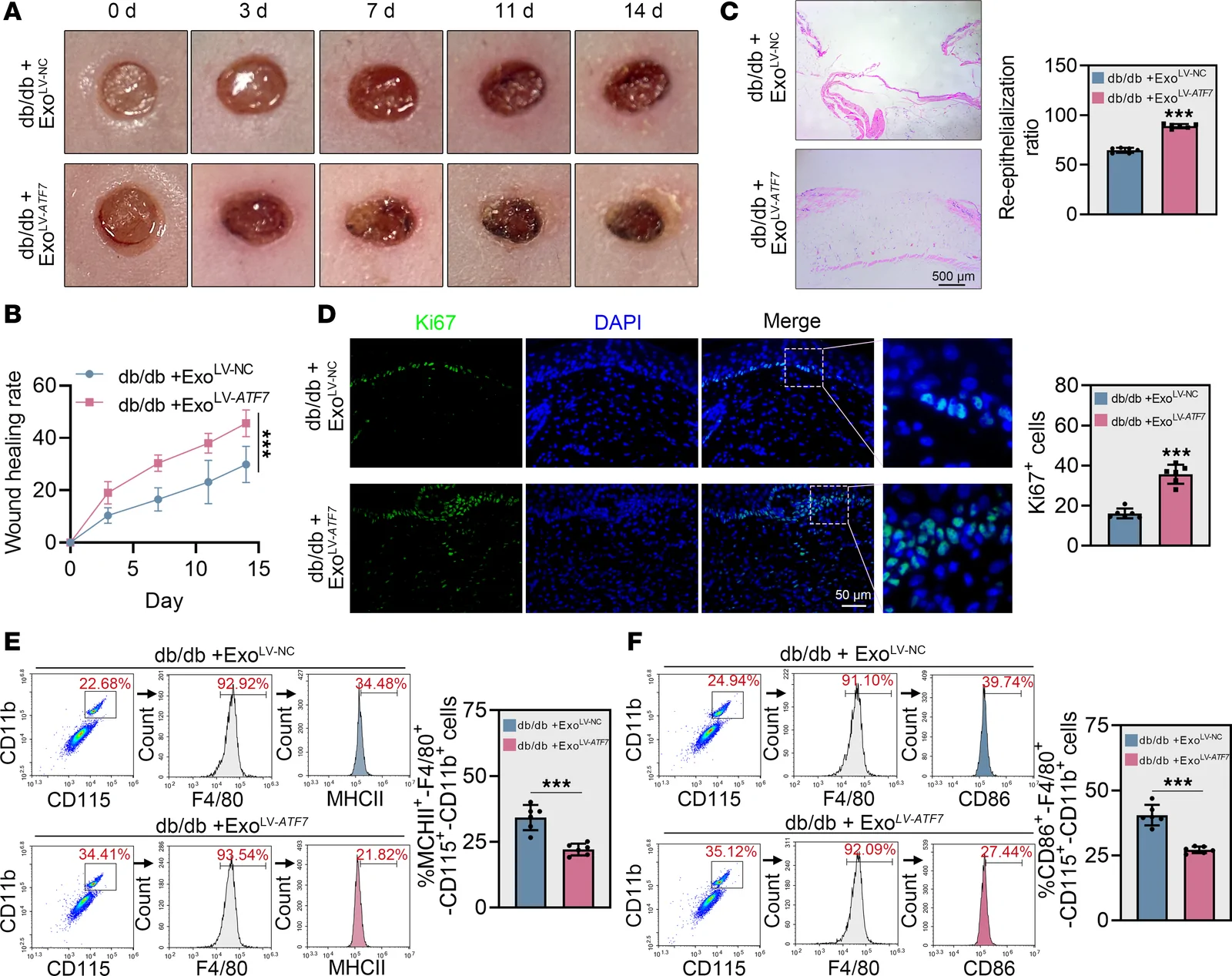
*ATF7*-overexpressing keratinocyte-derived exosomes promote diabetic wound healing in vivo. Exosomes were purified from conditioned media of HaCaT cells infected with LV-NC (Exo^LV-NC^) or LV-*ATF7* (Exo^LV-ATF7^). Full-thickness skin wounds were created on the dorsum of db/db mice, followed by injection of exosomes. (**A**) Representative wound photographs at indicated days after wounding (days 0, 3, 7, 11, and 14). (**B**) Quantitative analysis of wound closure rate. (**C**) Representative H&E-stained sections of wound tissues and quantification of re-epithelialization ratio from H&E-stained sections. Scale bar: 500 μm. (**D**) Representative immunofluorescence images showing Ki67 expression in wound tissues and quantitative analysis for Ki67^+^ cells. Nuclei counterstained with DAPI. Scale bar: 50 μm (original magnification: ×400). (**E**) Quantitative analysis of the percentage of M1 macrophages (MHCII^+^F4/80^+^CD115^+^CD11b^+^). (**F**) Quantitative analysis of the percentage of M1 macrophages (CD86^+^F4/80^+^CD115^+^CD11b^+^). Results are expressed as mean ± SD. ***P < 0.001 by 2-way ANOVA with Bonferroni’s post hoc test (**B**) or 2-tailed, unpaired Student’s *t* test (**C**–**F**). Each experimental group consisted of *n* = 6 animals.

Having established that ATF7 overexpression in keratinocytes suppresses M1 macrophage polarization in vitro, we next asked whether exosomes derived from *ATF7*-overexpressing keratinocytes are sufficient to promote diabetic wound healing in vivo. To this end, we purified exosomes from HaCaT cells infected with LV-NC (Exo^LV-NC^) or LV-*ATF7* (Exo^LV-ATF7^), and injected them into full-thickness skin wounds of db/db mice. As shown in Figure 6, A and B, Exo^LV-ATF7^ treatment accelerated wound closure compared with Exo^LV-NC^ treatment. Histological analysis by H&E staining revealed enhanced re-epithelialization, as evidenced by a reduced epithelial gap and an increased re-epithelialization ratio in Exo^LV-ATF7^–treated wounds (Figure 6C). IF staining for Ki67 showed an increase in proliferative keratinocytes in the Exo^LV-ATF7^ group (Figure 6D). Furthermore, flow cytometric analysis of wound tissues showed that Exo^LV-ATF7^ treatment decreased the percentage of M1 macrophages (MHCII^+^ and CD86^+^ populations) (Figure 6, E and F). Collectively, these results indicate that exosomes derived from *ATF7*-overexpressing keratinocytes promote healing in db/db mice.

### High-throughput omics screening identifies NOTCH1 as a downstream target of ATF7.

**Figure 7:**
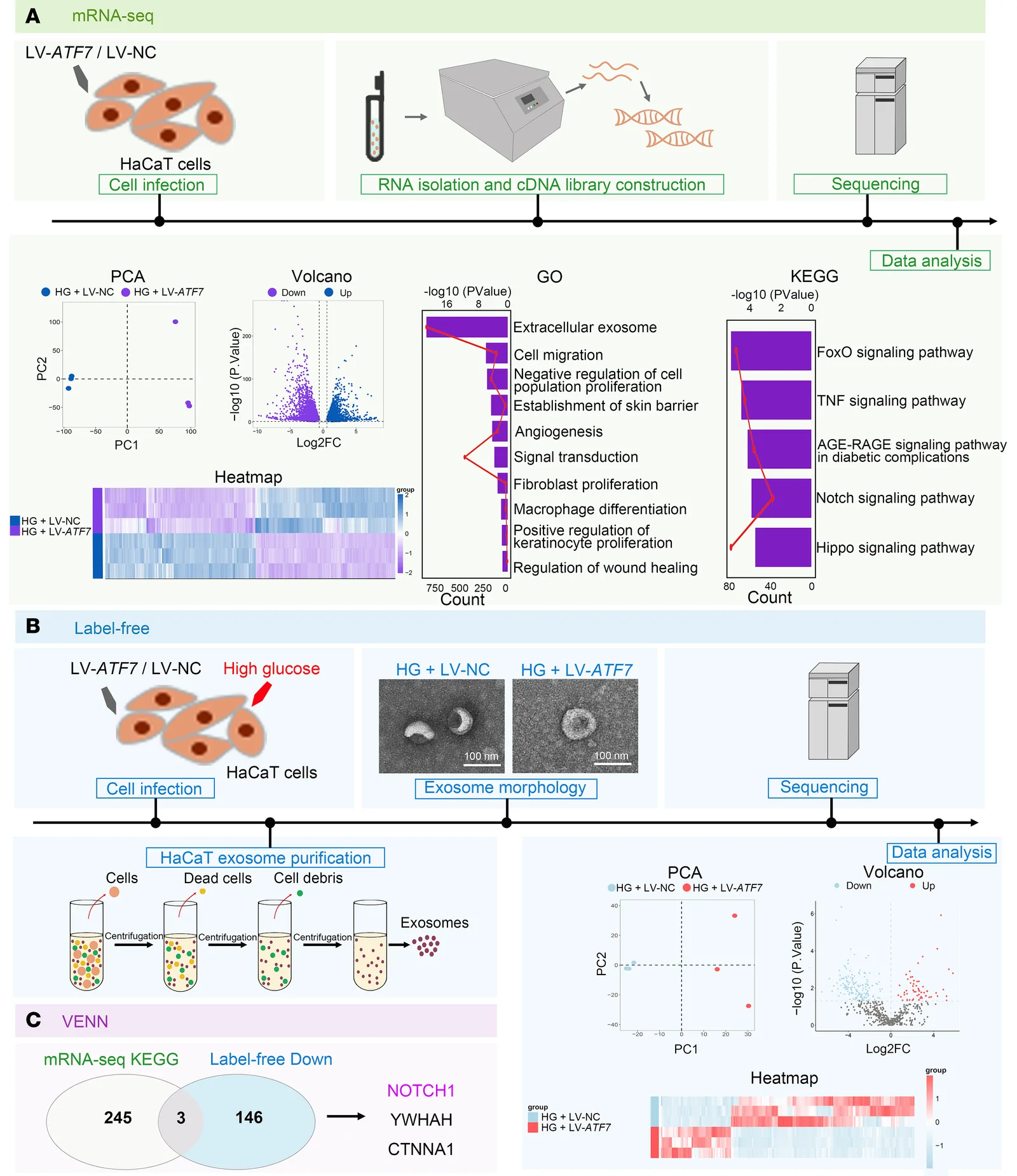
High-throughput omics screening identifies NOTCH1 as a downstream target of ATF7. (**A**) Transcriptomic analysis: HaCaT cells infected with LV-*ATF7* or LV-NC lentivirus (48 hours) were cultured under HG (24 hours) for RNA sequencing. Left to right: Principal component analysis (PCA); volcano plot of differentially expressed genes (DEGs); and cluster heatmap of all DEGs. GO and KEGG pathway enrichment analysis of upregulated and downregulated DEGs are shown. (**B**) Exosomal proteomics: Exosomes purified from HG-treated HaCaT cells were characterized by transmission electron microscopy. Label-free quantitative proteomics was performed. Left to right: PCA; volcano plot of differentially expressed proteins (DEPs); and cluster heatmap of all DEPs. (**C**) Target identification: Intersection analysis between factors enriched in the top 5 KEGG pathways from transcriptomics (**A**) and downregulated proteins from exosomal proteomics (**B**), yielding 3 overlapping molecules (NOTCH1, YWHAH, and CTNNA1). NOTCH1 was prioritized for validation.

To identify downstream molecular targets regulated by ATF7, HaCaT cells were infected with lentivirus and subsequently treated with HG. Transcriptome sequencing (RNA-seq) was then performed on these cells (Figure 7A). Principal component analysis (PCA) demonstrated clear separation between the experimental groups. Heatmap and volcano plot analysis visualized all differentially expressed genes (DEGs) identified by the RNA-seq. Gene Ontology (GO) enrichment analysis of upregulated and downregulated DEGs revealed enrichment in biological processes crucial for wound healing, including extracellular exosome, establishment of skin barrier, and regulation of wound healing. Similarly, Kyoto Encyclopedia of Genes and Genomes (KEGG) pathway enrichment analysis highlighted pathways implicated in wound healing, such as FoxO signaling pathway and Notch signaling pathway. In parallel, exosomes were purified from HG-treated, *ATF7*-overexpressing HaCaT cells and subjected to label-free quantitative proteomics (Figure 7B). This analysis yielded a set of differentially downregulated factors. Subsequently, we intersected the downregulated factors from label-free quantitative proteomics with the genes enriched in KEGG pathways identified by RNA-seq. This intersection yielded 3 candidate molecules: NOTCH1, YWHAH, and CTNNA1 (Figure 7C). Given the extensively documented role of the NOTCH1 signaling pathway in diabetic wound healing, including reports demonstrating its contribution to delayed healing in diabetic mice (13), NOTCH1 was selected for further investigation.

### ATF7 transcriptionally represses NOTCH1 and reduces exosomal N1ICD expression.

**Figure 8:**
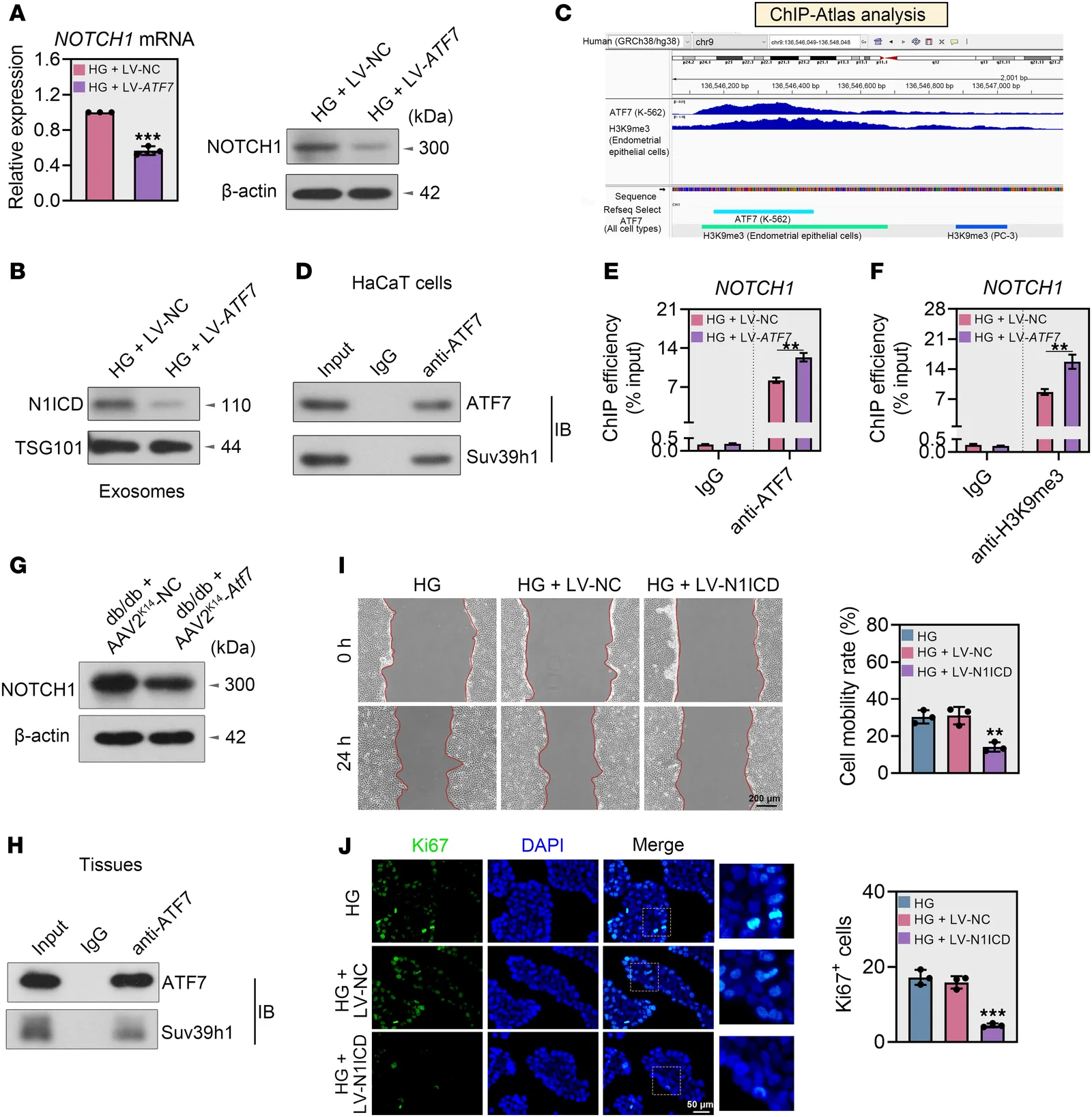
ATF7 transcriptionally represses NOTCH1 and reduces exosomal N1ICD expression. (**A**) *NOTCH1* mRNA and NOTCH1 protein expression in HG-treated (25 mM, 24 hours) HaCaT cells infected with LV-*ATF7* or LV-NC lentivirus (48 hours), analyzed by qRT-PCR and Western blotting. (**B**) N1ICD protein levels in exosomes purified from cells, detected by Western blotting. (**C**) Predicted binding sites of ATF7 and enrichment of H3K9me3 at the *NOTCH1* promoter region through the ChIP-Atlas database. (**D**) Co-IP analysis of ATF7 interaction with histone H3K9 methyltransferase Suv39h1 in HG-treated HaCaT cells (25 mM, 24 hours). IgG: negative control. (**E**) ChIP-qPCR confirming ATF7 binding to the *NOTCH1* promoter in LV-*ATF7*–infected, HG-treated HaCaT cells. (**F**) ChIP-qPCR analysis of H3K9me3 enrichment at the *NOTCH1* promoter. (**G**) Western blot analysis of NOTCH1 protein levels in wound tissues from db/db mice with or without keratinocyte-specific *ATF7* overexpression. (**H**) Co-IP analysis of ATF7 interaction with histone H3K9 methyltransferase Suv39h1 in wound tissues from db/db mice. HaCaT cells were infected with LV-NC or LV-N1ICD and cultured under HG conditions. (**I**) Cell migration ability was analyzed by the scratch wound healing assay. Scale bar: 200 μm. (**J**) Representative immunofluorescence images of Ki67 expression and quantitative analysis for Ki67^+^ cells. Nuclei counterstained with DAPI. Scale bar: 50 μm (original magnification: ×400). Results are expressed as mean ± SD. **P < 0.01, ***P < 0.001 by 2-tailed unpaired Student’s *t* test (**A**, **E**, and **F**) or 1-way ANOVA with Bonferroni’s post hoc test (**I** and **J**). Each experimental group consisted of *n* = 6 animals. All cell experiments were performed with *n* = 3 replicates.

Our investigations revealed that *ATF7* overexpression in HaCaT cells downregulated NOTCH1 expression (Figure 8A). Subsequently, exosomes were purified from these HaCaT cells, and analysis revealed a decrease in the expression of the NOTCH1 intracellular domain (N1ICD) within the exosomes (Figure 8B). Analysis using the ChIP-Atlas database predicted the binding for *ATF7* within the *NOTCH1* promoter region and identified enrichment of H3K9me3 at this promoter (Figure 8C). To validate these predictions, we performed a co-IP assay, which confirmed an interaction between ATF7 and the histone H3K9–specific tri-methyltransferase Suv39h1 (Figure 8D). Furthermore, a chromatin immunoprecipitation–qPCR (ChIP-qPCR) assay exhibited ATF7 binding to the *NOTCH1* promoter in HaCaT cells (Figure 8E). Critically, ChIP-qPCR analysis further revealed an increase in H3K9me3 enrichment at the *NOTCH1* promoter following *ATF7* overexpression (Figure 8F). To extend these mechanistic findings to the in vivo setting, we analyzed wound tissues from db/db mice. Western blotting revealed that NOTCH1 protein levels were reduced in *ATF7*-overexpressing wounds (Figure 8G). Co-IP using wound tissues showed that ATF7 interacted with Suv39h1 in vivo (Figure 8H). Moreover, IF staining exhibited an increased H3K9me3 signal within tissues of *ATF7*-overexpressing wounds (Supplemental Figure 1F). To further test the functional relevance of NOTCH1, HaCaT cells were infected with a lentivirus overexpressing N1ICD (Supplemental Figure 1E) and then cultured under HG conditions. N1ICD overexpression impaired cell migration in a wound healing assay (Figure 8I) and decreased the number of Ki67-positive cells (Figure 8J). Altogether, these results imply that ATF7 recruits the histone methyltransferase Suv39h1 to the *NOTCH1* promoter, leading to increased H3K9me3 deposition, transcriptional repression of NOTCH1, and consequently reduced levels of both cellular NOTCH1 and exosomal N1ICD.

### Keratinocyte ATF7 inhibits macrophage M1 polarization by reducing exosomal N1ICD secretion.

**Figure 9:**
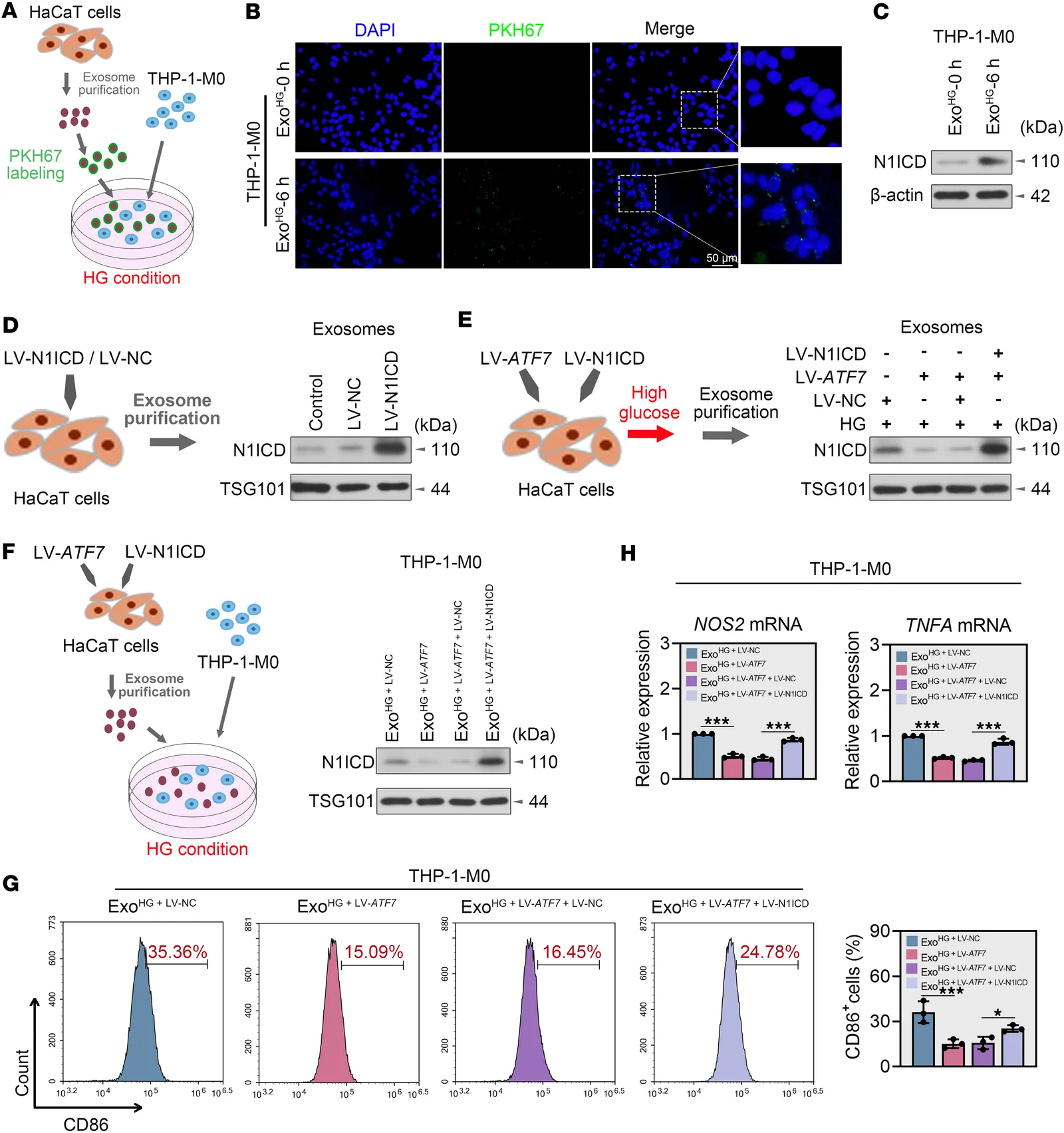
Keratinocyte ATF7 inhibits macrophage M1 polarization by reducing exosomal N1ICD secretion. (**A**) HaCaT cells and THP-1-M0 macrophages were pretreated with 25 mM HG (24 hours). Exosomes purified from HaCaT cells were labeled with PKH67 (green) and co-incubated with macrophages (6 hours). (**B**) Confocal microscopy images showing PKH67^+^ exosome internalization by macrophages (green, exosomes; blue, DAPI). Scale bar: 50 μm (original magnification: ×400). (**C**) N1ICD protein levels in macrophages after exosome incubation, detected by Western blotting. (**D**) HaCaT cells were infected with N1ICD-overexpressing lentivirus (LV-N1ICD, 48 hours). Exosomal N1ICD levels were analyzed by Western blotting. (**E**) HaCaT cells co-infected with LV-*ATF7* or LV-N1ICD (48 hours) and treated with HG (24 hours). Exosomal N1ICD levels were analyzed by Western blotting. (**F**) Macrophages were incubated with exosomes, and recipient macrophage N1ICD levels were detected by Western blotting. (**G**) Flow cytometric analysis of CD86^+^ macrophages. (**H**) mRNA expression of *NOS2* and *TNFA* in macrophages. Results are expressed as mean ± SD. *P < 0.05, ***P < 0.001 by 1-way ANOVA with Bonferroni’s post hoc test. All cell experiments were performed with *n* = 3 replicates.

To elucidate the mechanism of exosome-mediated communication, exosomes derived from HaCaT cells were labeled with the green fluorescent dye PKH67 and subsequently coincubated with THP-1-M0 macrophages (Figure 9A). Microscopy analysis revealed an increase in exosome uptake by macrophages after 6 hours of incubation (Figure 9B). Concomitantly, the expression of N1ICD within these macrophages was also elevated (Figure 9C). Exosomes purified from these cells exhibited increased levels of N1ICD protein (Figure 9D). We then purified exosomes from HaCaT cells that were infected with lentivirus and treated with HG. Analysis showed that ATF7 overexpression in keratinocytes reduced exosomal N1ICD levels. This ATF7-mediated reduction in exosomal N1ICD was reversed by overexpression of N1ICD (Figure 9E). These results suggest that the transfer of keratinocyte-derived exosomal N1ICD to macrophages is regulated by ATF7. To assess the impact on macrophage polarization, THP-1-M0 macrophages were cocultured with HaCaT cells coinfected with the relevant lentiviruses and treated with HG (Figure 9F). Flow cytometric analysis revealed that *ATF7* overexpression in keratinocytes decreased the percentage of CD86^+^ macrophages. Notably, this *ATF7*-induced decrease was partially reversed when N1ICD was co-overexpressed (Figure 9G). Furthermore, the *ATF7* overexpression–mediated downregulation of *NOS2* and *TNFA* expression in cocultured macrophages was also counteracted by N1ICD co-overexpression (Figure 9H). These findings indicate that *ATF7* in keratinocytes inhibits macrophage M1 polarization by reducing the secretion of N1ICD within exosomes.

## Discussion

**Figure 10:**
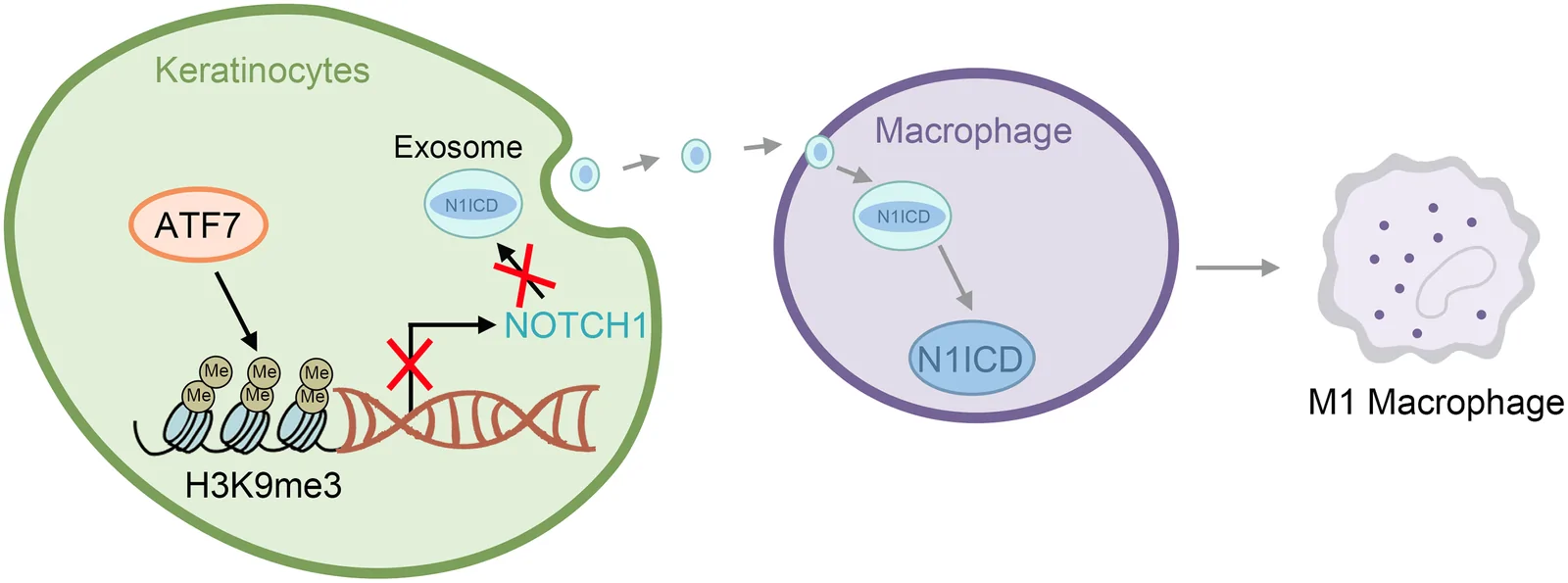
Proposed mechanism. ATF7 promotes diabetic wound healing by repressing NOTCH1 transcription via H3K9me3, thereby reducing exosomal N1ICD secretion from keratinocytes and inhibiting macrophage M1 polarization.

This study reveals an important role for ATF7 in diabetic wound healing. We demonstrate that *Atf7* overexpression promotes wound closure in diabetic db/db mice. ATF7 achieves this function by acting as a transcriptional repressor; it recruits the histone methyltransferase Suv39h1 to the *NOTCH1* promoter. This recruitment facilitates trimethylation of H3K9me3, leading to transcriptional silencing of *NOTCH1* in keratinocytes. A key functional consequence of this ATF7-mediated NOTCH1 suppression is a reduction in the levels of N1ICD within keratinocyte-derived exosomes. Consequently, the delivery of this signaling molecule via exosomes to wound macrophages is diminished. This reduction in exosomal N1ICD transfer inhibits proinflammatory M1 polarization (Figure 10). These findings position ATF7 as a critical transcriptional hub in diabetic wound repair and highlight the therapeutic potential of modulating the ATF7/NOTCH1/exosome axis.

We identify ATF7 as a beneficial regulator in the diabetic wound milieu. Its previously established roles in cellular stress responses and NF-κB pathway suppression (10), along with its pro-proliferative and migratory capacities (11, 12), are crucial for tissue repair. We observed an upregulation of *Atf7* in wounds of db/db mice, which likely represents a physiological compensatory response to the diabetic stress within the wound environment. However, this endogenous upregulation appears insufficient to ameliorate the pathological state. Subsequent in vivo and in vitro overexpression of ATF7 confirmed our hypothesis that augmenting ATF7 levels promotes wound healing. Furthermore, utilizing transcriptomic analysis combined with exosomal proteomic screening, we identified NOTCH1 as a potential downstream target of ATF7.

NOTCH1, a member of the NOTCH protein family, is involved in diverse processes including cell proliferation, migration, and angiogenesis (14). The NOTCH receptor family comprises 4 members (NOTCH1–4) located on the cell membrane, activated by binding to ligands (such as JAG1, -2, or Delta-like 1, 3, and 4) on neighboring cells (15). Upon activation, the NOTCH1 receptor undergoes cleavage, releasing N1ICD, which translocates to the nucleus to regulate gene expression (16, 17). The role of NOTCH signaling in diabetic wound healing is well documented. For instance, Shao et al. demonstrated that NOTCH1 activation (knockin) inhibits fibroblast proliferation and migration, delaying wound healing in diabetic mice (13). Conversely, the NOTCH1 inhibitor DAPT promotes collagen accumulation and accelerates diabetic wound healing (18). In keratinocytes, HG treatment activates the NOTCH1 pathway, and both DAPT treatment and keratinocyte-specific NOTCH1 knockout promote skin wound healing in diabetic mice (19). Our findings demonstrate that ATF7 suppresses NOTCH1 transcription via H3K9me3 modification. Crucially, the therapeutic benefits of ATF7 overexpression were reversed by forced expression of N1ICD, unequivocally confirming the inhibitory effect of ATF7 on NOTCH1 signaling in the context of diabetic wound healing.

Moreover, multiple studies report that the NOTCH1 pathway promotes macrophage M1 polarization (20, 21). During diabetic wound healing, blocking the NOTCH1 pathway suppresses M1 polarization and mitigates the detrimental effects of angiogenesis inhibitors on wound repair (22). Evidence suggests that glioma stem cell–derived exosomes are enriched in NOTCH1 protein. Upon uptake by recipient cells, these exosomes release the N1ICD, influencing downstream gene transcription and target cell function (23). In our study, ATF7 overexpression suppressed markers of M1 polarization in wound macrophages, achieved through NOTCH1 blockade, and reduced exosomal N1ICD transfer. This shift in macrophage polarization phenotype is central to ATF7’s therapeutic effect, as sustained M1 inflammation is a major driver of chronicity in diabetic wounds, complementing prior findings.

Therapeutically, elucidating the ATF7/NOTCH1/exosome axis reveals multiple promising intervention points. Our rescue experiments provide genetic validation for targeting this pathway. Potential strategies include developing pharmacological agonists of ATF7, inhibiting NOTCH1 signaling using inhibitors like peptide inhibitor TAT-ANK (24) and the clinically relevant γ-secretase inhibitor DAPT (25). Alternatively, exosomes have been engineered to specifically deplete N1ICD cargo, analogous to promising applications of stem cell–derived exosomes (26). Targeting ATF7 or its downstream effectors offers potential advantages over growth factor therapies (VEGF and FGF) by acting upstream at the level of a transcriptional regulator, potentially yielding broader and more sustained effects while circumventing issues of protein instability or short half-life encountered by some growth factors in the harsh wound environment (27, 28).

Several limitations of this study should be acknowledged. First, while our experiments in db/db mice demonstrate that keratinocyte-specific *Atf7* overexpression accelerates diabetic wound healing, and complementary in vitro experiments show that *ATF7* knockdown impairs keratinocyte proliferation and migration, in vivo evidence from keratinocyte-specific *Atf7*-deficient mice is lacking. We recognize that the availability of such evidence would provide more proof of the important role of ATF7 in diabetic healing. Second, validation of our findings in human diabetic wounds is still at a preliminary stage. We collected human diabetic foot ulcer (DFU) tissues (*n* = 6) and non-diabetic normal skin tissues (*n* = 2). Western blot analysis revealed that both ATF7 and NOTCH1 proteins were elevated in DFU samples compared with controls (Supplemental Figure 1A). We interpret this co-upregulation as an endogenous protective attempt that is insufficient to overcome the complex chronic wound microenvironment. Consequently, NOTCH1 remains elevated and healing fails. This interpretation aligns with the literature showing that NOTCH1 impairs diabetic wound healing and underscores the therapeutic rationale for exogenously enhancing ATF7 activity — exactly as we validated in our mouse model. However, due to the small sample size of normal skin, definitive conclusions regarding ATF7 and NOTCH1 expression levels in human diabetic wounds await confirmation in larger cohorts. We are actively continuing sample collection and will examine ATF7, NOTCH1, and posttranslational regulators in future studies once sufficient tissue is obtained.

In conclusion, this study advances our understanding of the pathogenesis and repair processes in diabetic wounds by identifying ATF7 as a critical pro-healing regulator. It provides insights for developing innovative strategies to restore healing in chronic diabetic wounds.

## Methods

### Sex as a biological variable.

Human tissues were collected from both male and female donors, and sex was not incorporated as a variable in the analysis of human samples. Male db/db and C57BL/6 mice were used in animal experiments. The relevance of the findings to females remains to be investigated in future studies.

### Viral vector construction.

For AAV production, the coding sequence (CDS) of *Atf7* (NM_001310070) was inserted into the multiple cloning site (KpnI/XhoI restriction sites) of an AAV2 serotype vector under the control of the keratinocyte-specific *K14* promoter. The recombinant plasmid was transfected into AAV-293 cells (iCell Bioscience) using Lipofectamine 3000 (Invitrogen). Seventy-two hours after transfection, viral particles were harvested, filtered through a 0.45 μm membrane, and concentrated. The final titer of AAV2^K14^-*Atf7* was determined to be 1 × 10^12^ vector genomes (vg)/mL.

For lentiviral vector production, the CDS of *ATF7* (NM_001130060.2) or N1ICD (NM_017617.5) was inserted into the multiple cloning site (XhoI/NotI sites) of the pLVX-IRES-Puro vector (Fenghui Biotechnology). Recombinant plasmids were transfected with packaging plasmids (psPAX2 and pMD2.G) into HEK293T cells (Icell Bioscience) using Lipofectamine 3000. Viral supernatants were collected at 48–72 hours after transfection, filtered through 0.45 μm membranes, and concentrated via ultracentrifugation. Final titers were determined: LV-*ATF7*, 1.8 × 10^8^ transducing units (TU)/mL; LV-N1ICD, 1.9 × 10^8^ TU/mL.

### Animals.

Ten-week-old male mice were used for full-thickness excisional wound studies (*n* = 6 mice). Diabetic db/db mice were obtained from Cavens Laboratory Animal Co., Ltd., and non-diabetic C57BL/6 mice from HuaChuang Sino Pharmaceutical Technology Co., Ltd. Before experiments, fasting blood glucose was measured, and db/db mice with levels of greater than 300 mg/dL were included.

Wound surgery was performed following established protocols (29), and anesthesia was induced with 3% isoflurane and maintained with 2% isoflurane. Dorsal hair was shaved, and 2 symmetrical 4-mm full-thickness excisional wounds were created using a sterile biopsy punch. For ATF7 overexpression, intradermal injections of AAV2^K14^-*Atf7* (1 × 10^12^ vg/mL) were administered at 4 points (20 μL per site) around predetermined wound sites. Three weeks after injection, wounding was performed as described above. At designated time points, wound images were captured, followed by euthanasia via CO_2_. Wound tissues, including the peripheral margins, were harvested for analysis.

Additionally, exosomes were purified from HaCaT cells infected with LV-NC (Exo^LV-NC^) or LV-*ATF7* (Exo^LV-ATF7^). After wound surgery, from day 1 to day 5 after wounding, 100 μg of exosomes suspended in 100 μL of PBS were injected around each wound at 4 injection sites (25 μL per site). Wound healing was assessed by measuring wound areas on day 14 after wounding.

### Human samples.

Human skin tissue samples were collected from patients with DFUs (*n* = 6) and non-diabetic patients (*n* = 2). Tissue samples from patients were stored at –80°C for Western blotting.

### Cell culture.

HaCaT and THP-1 monocytes were obtained from Icell Bioscience. HaCaT cells were maintained in DMEM (Servicebio) supplemented with 10% fetal bovine serum (FBS), while THP-1 cells were cultured in RPMI-1640 medium (Solarbio) containing 10% FBS. All cells were incubated at 37°C in a humidified 5% CO_2_. For HG treatment, cells were exposed to media containing either 25 mM glucose (HG group) or 5.5 mM glucose (normal glucose control) for 24 hours. THP-1 monocytes were differentiated into M0 macrophages (THP-1-M0) by treatment with 20 nM phorbol 12-myristate 13-acetate (PMA) for 36 hours. For coculture experiments, HaCaT cells and THP-1-M0 macrophages were cocultured for 24 hours using Transwell inserts with HaCaT cells in the upper chamber and macrophages in the lower chamber. For the rescue experiments, THP-1 monocytes were polarized to an M1 phenotype by treatment with LPS (100 ng/mL) and IFN-γ (20 ng/mL) for 24 hours.

### qRT-PCR.

Total RNA from cells or tissues was isolated using TRIpure reagent (BioTeke), followed by reverse transcription into cDNA. qRT-PCR was performed using the Pangaea 3 Real-Time PCR System (Aperbio). β-Actin served as the endogenous reference gene for normalization. Primer sequences are provided in Supplemental Table 1.

### Western blot.

Protein lysates were extracted from cells or tissues using RIPA Lysis Buffer (Proteintech). Protein concentrations were quantified using a BCA Protein Assay Kit (Beyotime). Equal amounts of protein were separated by SDS-PAGE and transferred onto PVDF membranes (Millipore). Membranes were blocked with nonfat milk in TBST, followed by overnight incubation at 4°C with primary antibodies anti-ATF7 (Affinity, DF3105), anti-CD63 (Proteintech, 67605-1-Ig, clone 3D4D1), anti-TSG101 (Proteintech, 28283-1-AP), anti-NOTCH1 (Abclonal, A7636), and anti-N1ICD (Cell Signaling Technology, 4147, clone D3B8). After TBST washes, membranes were incubated with secondary antibodies for 1 hour. Protein bands were visualized using ECL Prime Substrate (Proteintech) and imaged. Band intensity was quantified using Gel-Pro Analyzer software version 4 (Media Cybernetics).

### Histological analysis.

Wound tissues were fixed in neutral-buffered formalin, dehydrated, cleared in xylene, and embedded in paraffin. Tissue sections (5 μm thickness) were cut using a rotary microtome (Leica). Sections (5 μm) were cut using a rotary microtome (Leica), deparaffinized in xylene, rehydrated through graded alcohols, and stained routinely with H&E. Morphological assessment was performed under an Olympus microscope. Re-epithelialization and granulation tissue deposition were determined. Re-epithelialization was quantified as (length of neo-epithelium/total wound length) × 100.

### IF.

Paraffin-embedded wound tissue sections or fixed cell monolayers were blocked, and primary antibody incubation was performed overnight at 4°C using anti-Ki67 (Proteintech, 27309-1-AP), anti-ATF7 (Affinity, DF3105), anti-K14 (Santa Cruz Biotechnology, sc-53253, clone LL001), anti-CD206 (Zenbio, 251716), and anti-H3K9me3 (Zenbio, R40010, clone R08-7S-1). After PBS washes, samples were incubated for 1 hour with secondary antibody Cy3-conjugated goat anti-rabbit IgG (Proteintech, SA00009-2), FITC-conjugated goat anti-mouse IgG (Proteintech, SA00003-1), or FITC-conjugated goat anti-rabbit IgG (Proteintech, SA00003-2). Nuclei were counterstained with DAPI, and images were captured using an Olympus BX53 fluorescence microscope.

### Flow cytometric analysis.

For tissue-derived single-cell suspensions, skin wound tissues were minced into fragments under sterile conditions, digested with Dispase (Biosharp) at 4°C overnight, followed by hyaluronidase and collagenase treatment at 37°C for 2 hours. After terminating digestion, cell suspensions were filtered through a strainer and centrifuged. Cells were washed once with PBS, resuspended in buffer, and incubated with anti-CD16/CD32 blocking antibody (Bio X Cell, BE0307) for 30 minutes. After washing, cells were resuspended in buffer and stained with fluorochrome-conjugated antibodies anti-mouse MHCII (FITC; Elabscience, E-AB-F0990C), anti-mouse F4/80 (APC; Elabscience, E-AB-F0995E), anti-mouse CD115 (PE; Elabscience, E-AB-F1107D), anti-mouse CD11b (PerCP; Elabscience, E-AB-F1081J), and anti-mouse CD86 (FITC; Elabscience, E-AB-F0994C). Cells were washed twice with PBS, centrifuged, resuspended in buffer, and analyzed immediately. For coculture systems, lower-chamber macrophages were collected, washed with PBS, resuspended in buffer, and stained with anti-human CD86 (PE; Elabscience, E-AB-F1012D) or anti-human MHCII (FITC; Santa Cruz Biotechnology, sc-59318) for 30 minutes, followed by 2 PBS washes and resuspension in buffer. All samples were processed using an Agilent flow cytometer.

### ELISA analysis.

Skin wound tissues were mechanically homogenized in a tissue grinder under ice-bath conditions. Supernatants were collected, and protein concentrations quantified using a BCA Protein Assay Kit. Cytokine levels were measured using commercial ELISA kits according to manufacturer protocols: mouse TNF-α (Lianke Biotech, EK282), mouse IL-1B (Lianke Biotech, EK201B), and mouse IL-6 (Lianke Biotech, EK206), with absorbance read on a microplate reader.

### CCK-8 assay.

Cells were seeded in 96-well plates at a density of 5 × 10^3^ cells/well and incubated at 37°C with 5% CO_2_. Following incubation, 10 μL of CCK-8 reagent (BioSharp) was added to each well, and plates were further incubated for 2 hours. Absorbance was measured at 450 nm using a microplate reader.

### Cell migration assay.

Cells were cultured to 90%–100% confluence, pretreated with serum-free medium containing 10 μg/mL mitomycin C (MedChemExpress) for 1 hour, after which a standardized wound was created using a sterile pipette tip. Following PBS washing to remove debris, cells were maintained in serum-free medium. Wound closure was monitored at 0 hours and 24 hours after scratching using a microscope.

### ChIP assay.

ChIP was performed using the ChIP Assay Kit (Beyotime). Briefly, cells were cross-linked in 1% formaldehyde, quenched with Glycine Solution, and washed twice with ice-cold PBS containing 1 mM PMSF. Cells were resuspended in SDS Lysis Buffer and incubated on ice for 10 minutes. Chromatin was sonicated to shear DNA. After centrifuging sonicated lysates, supernatants were diluted 10-fold in ChIP Dilution Buffer (1 mM PMSF). A 20 μL aliquot was saved as the Input control; remaining lysates were precleared with Protein A/G Agarose/Salmon Sperm DNA, then incubated overnight at 4°C with anti-ATF7, anti-H3K9me3, or control IgG. Immune complexes were precipitated with Protein A/G Agarose/Salmon Sperm DNA, followed by sequential washes. Purified DNA was analyzed by qPCR.

### Co-IP.

Whole-cell lysates were extracted using RIPA buffer, and protein concentrations were quantified by BCA assay. For IP, a Co-IP Kit (Thermo Fisher Scientific) was used. Lysates were bound to AminoLink Plus Resin precoupled with anti-ATF7 (Abcam, ab87844) or anti-Suv39h1 (Proteintech, 10574-1-AP), with IgG as a negative control. Beads were washed, and immunoprecipitated complexes were analyzed by Western blotting.

### Bioinformatic analysis.

Transcriptomic data from the GSE182906 dataset (day 14) were retrieved from the NCBI GEO database (https://www.ncbi.nlm.nih.gov/geo/), and differentially upregulated genes were screened using thresholds of |logFC| of 1.5 or greater and a *P* value of less than 0.05. TFs were identified by intersecting these genes with the *Mus* *musculus* TF dataset (https://guolab.wchscu.cn/AnimalTFDB4/#/). The binding between ATF7 and the *NOTCH1* promoter region was predicted using ChIP-Atlas (https://chip-atlas.org/).

### High-throughput omics analysis.

For transcriptomic sequencing, mRNA was enriched from total RNA using Oligo dT magnetic beads, followed by cDNA synthesis. Libraries were prepared through end repair, A-tailing, and PCR amplification, with quality-controlled libraries quantified and sequenced on the Illumina NovaSeq 6000 platform. Raw reads were processed via base calling and quality filtering using the Illumina platform. For proteomic analysis, protein samples underwent reduction/alkylation, tryptic digestion, desalting, and lyophilization. Then the samples were analyzed by high-performance liquid chromatography. Peptide identification and protein inference were performed.

### Statistics.

Animal experiments were performed with 6 replicates, while cell-based assays were conducted with 3 replicates. Data are presented as mean ± standard deviation (SD). Comparisons between 2 groups were analyzed using 2-tailed, unpaired Student’s *t* test. For multiple-group comparisons, 1-way analysis of variance (ANOVA) with Bonferroni’s post hoc test was applied. Two-way analysis of ANOVA with Bonferroni’s post hoc test was applied for wound healing data. Statistical significance was defined as a *P* value of less than 0.05, with all analysis performed using GraphPad Prism.

### Study approval.

Human tissue collection and use were approved by the Ethics Committee of the First Affiliated Hospital of Henan University of Science and Technology (Luoyang, China, approval number: 2026-03-H0040) in accordance with the Declaration of Helsinki. Written, informed consent was obtained from patients. All animal experiments were approved by the Ethics Committee of the First Affiliated Hospital of Henan University of Science and Technology (Luoyang, China, approval number: D-2025-B003) and were performed in accordance with the NIH *Guide for the Care and Use of Laboratory Animals* (National Academies Press, 2011).

### Data availability.

Genomic data generated in this manuscript have been deposited in the NCBI’s GEO database (GSE326162). The proteomics data were deposited in the iProX database (PXD076554). Values for each data point presented in the graphs can be found in the Supporting Data Values file.

## Author contributions

PX, BC, and LW designed the research studies. PX, YX, LF, JK, and XH conducted experiments. PX, YX, and LF acquired data. PX, YX, and HT analyzed data. PX and YX wrote the manuscript. BC and LW reviewed and revised the manuscript, and provided administrative and material support and study supervision.

## Conflict of interest

The authors have declared that no conflict of interest exists.

## Funding support

- National Natural Science Foundation of China (82102338 to PX).
- Joint Construction Project of Henan Medical Science and Technology Research Program (LHGJ20230449 to XH).

## Supplementary Material

Supplemental data

Unedited blot and gel images

Supporting data values

## Version 1 08/24/2026

Electronic publication
