# Supplementary material for: ATF7 drives diabetic wound healing via NOTCH1 repression and N1ICD-dependent macrophage polarization control
Source: JCI Insight. 2026 Aug 24;11(16):e201178. doi: 10.1172/jci.insight.201178 (PMC13502182; doi:10.1172/jci.insight.201178)
Supplement: Supplementary file 1 [file jciinsight-11-201178-s290.pdf]

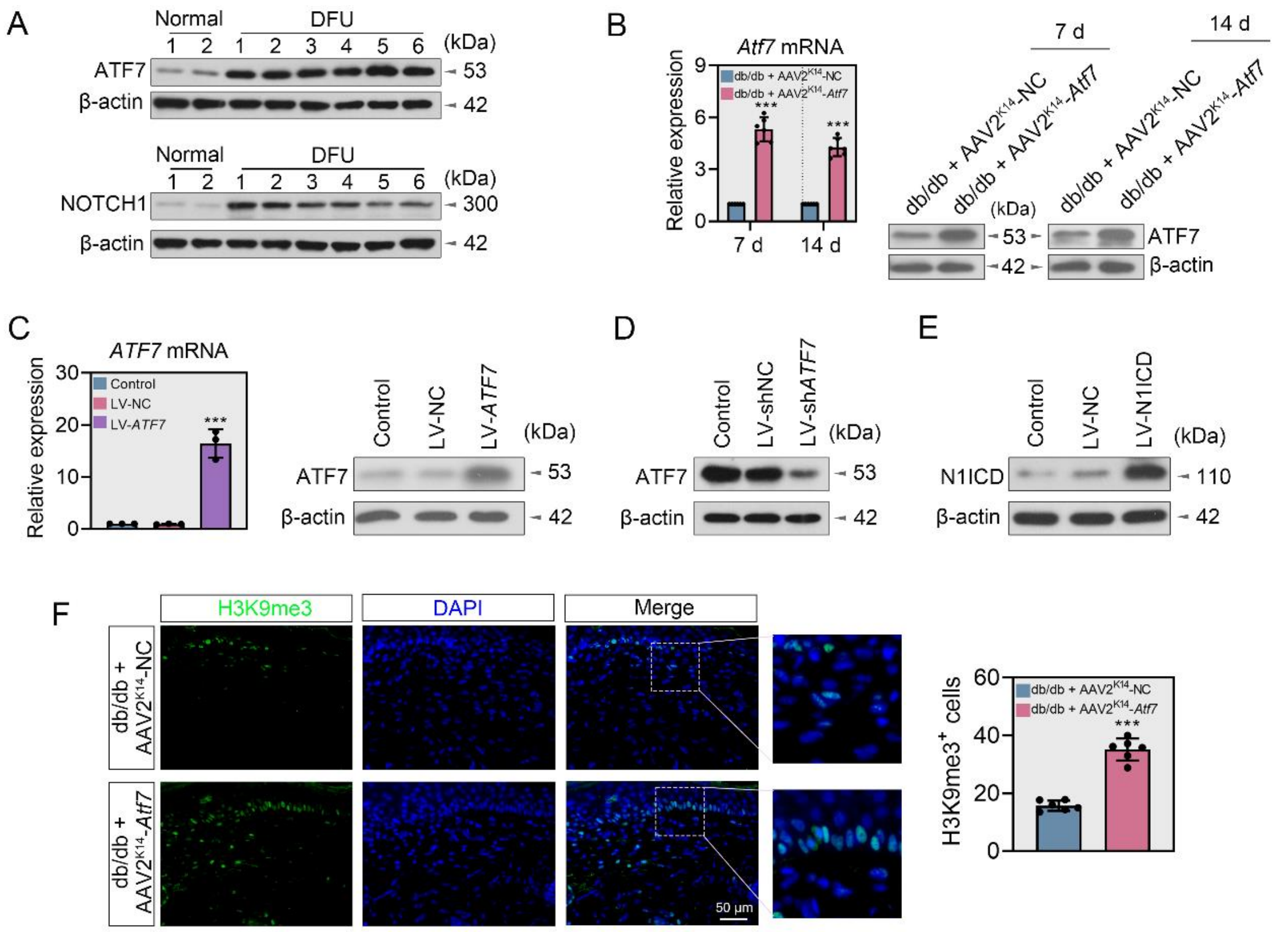

Figure. S1 (A) Protein expression of ATF7 and NOTCH1 in human diabetic foot ulcer (DFU) tissues (n = 6) and non-diabetic control skin samples (n = 2). (B) qRT-PCR and western blot analysis of ATF7 expression in wound tissues. (C) Human keratinocyte HaCaT cells were infected with LV-*ATF7* or control lentivirus (LV-NC) for 48 h. *ATF7* mRNA and ATF7 protein expression were validated by qRT-PCR and western blot, respectively. (D) HaCaT cells were infected with LV-sh*ATF7* or control lentivirus (LV-shNC) for 48 h. Protein expression was validated by western blot. (E) HaCaT cells were infected with LV-N1ICD or control lentivirus (LV-NC) for 48 h. Protein expression was validated by western blot. (F) Representative immunofluorescence images of H3K9me3 expression and quantitative analysis for H3K9me3-positive cells. Nuclei counterstained with DAPI. Scale bars: 50 μm. Results are expressed as means ± SD. ***p < 0.001. Cell experiments were performed with n=3 replicates, animal

experiments were performed with n=6 replicates. Statistical analysis was performed by two-tailed unpaired Student's t-test (B, F) and 1-way ANOVA (C).

Gene	Forward (5’-3’)	Reverse (5’-3’)
Mus *Nos2*	CACCACCCTCCTCGTTC	CAATCCACAACTCGCTCC
Mus *Cd86*	ATGGGCTCGTATGATTGT	CTTCTTAGGTTTCGGGTG
Mus *Atf7*	ATCCAACAGGCAAATAGG	CTTCTGACGGCATAGGG
Mus MHCII	CAGCCAAGATCAAAGTGCG	GCCAAGCCCGAGGAAGA
Mus *Arg1*	TATCTGCCAAAGACATCG	ATCACCTTGCCAATCCC
Mus *Cd206*	GCAAGTGATTTGGAGGCT	ATAGGAAACGGGAGAACC
Homo *IL1B*	TATTACAGTGGCAATGAGG	ATGAAGGGAAAGAAGGTG
Homo *IL6*	GTCCAGTTGCCTTCTCCC	GCCTCTTTGCTGCTTTCA
Homo *NOS2*	AGCGGTAACAAAGGAGATAG	GGGAACACGGTGATGG
Homo *TNFA*	CGAGTGACAAGCCTGTAGCC	TGAAGAGGACCTGGGAGTAGAT
Homo *ATF7*	TGTGAGGAGGTGGGACTCTT	TTGGGGTAACCTCCTCCACT
Homo *CD86*	CTCTGGTGCTGCTCCTCT	GGGTCCAACTGTCCGAAT
Homo MHCII	CAGGCGAGTTTATGTTTGAC	ATGATTTCCAGGTTGGCTTT
Homo *NOTCH1*	TGAATGGCGGGAAGTGTGAA	ACTTGTACTCCGTCAGCGTG

Supplementary Table 1. Sequences of qRT-PCR primers (5’-3’)
