# Supplementary material for: ATF7 drives diabetic wound healing via NOTCH1 repression and N1ICD-dependent macrophage polarization control
Source: JCI Insight. 2026 Aug 24;11(16):e201178. doi: 10.1172/jci.insight.201178 (PMC13502182; doi:10.1172/jci.insight.201178)

Full unedited gel for Figure 1B

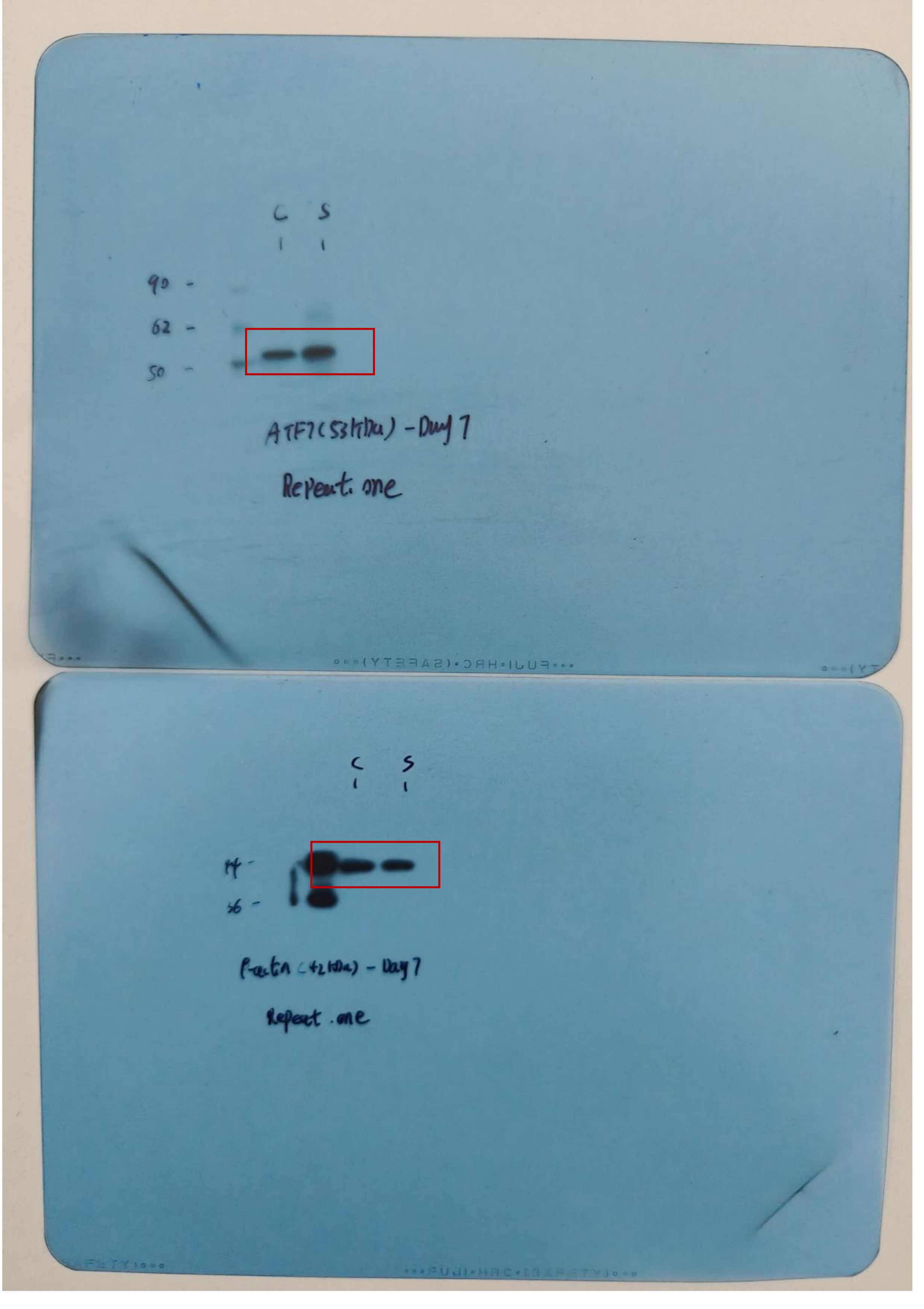

Full unedited gel for Figure 1B

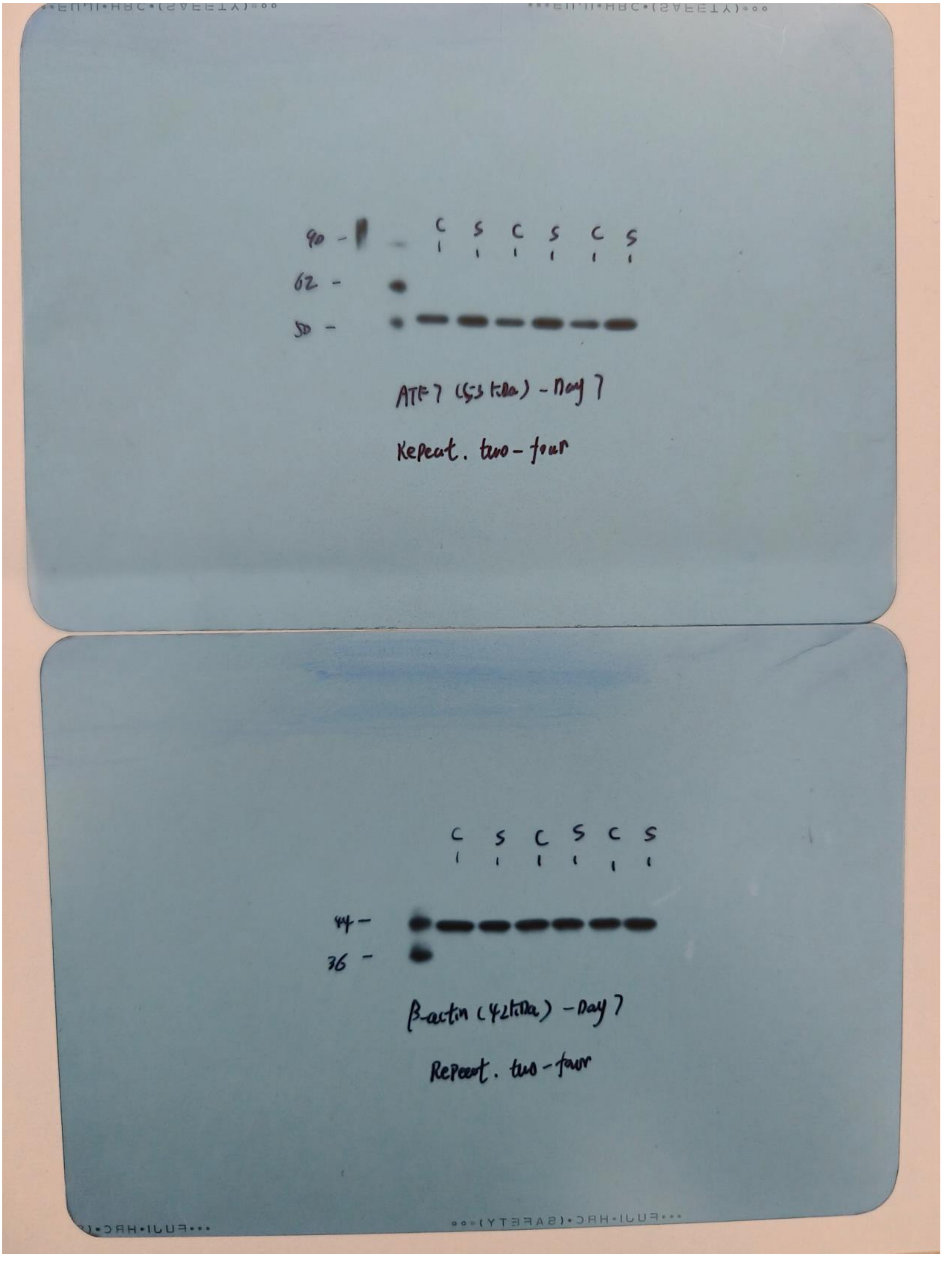

Full unedited gel for Figure 1B

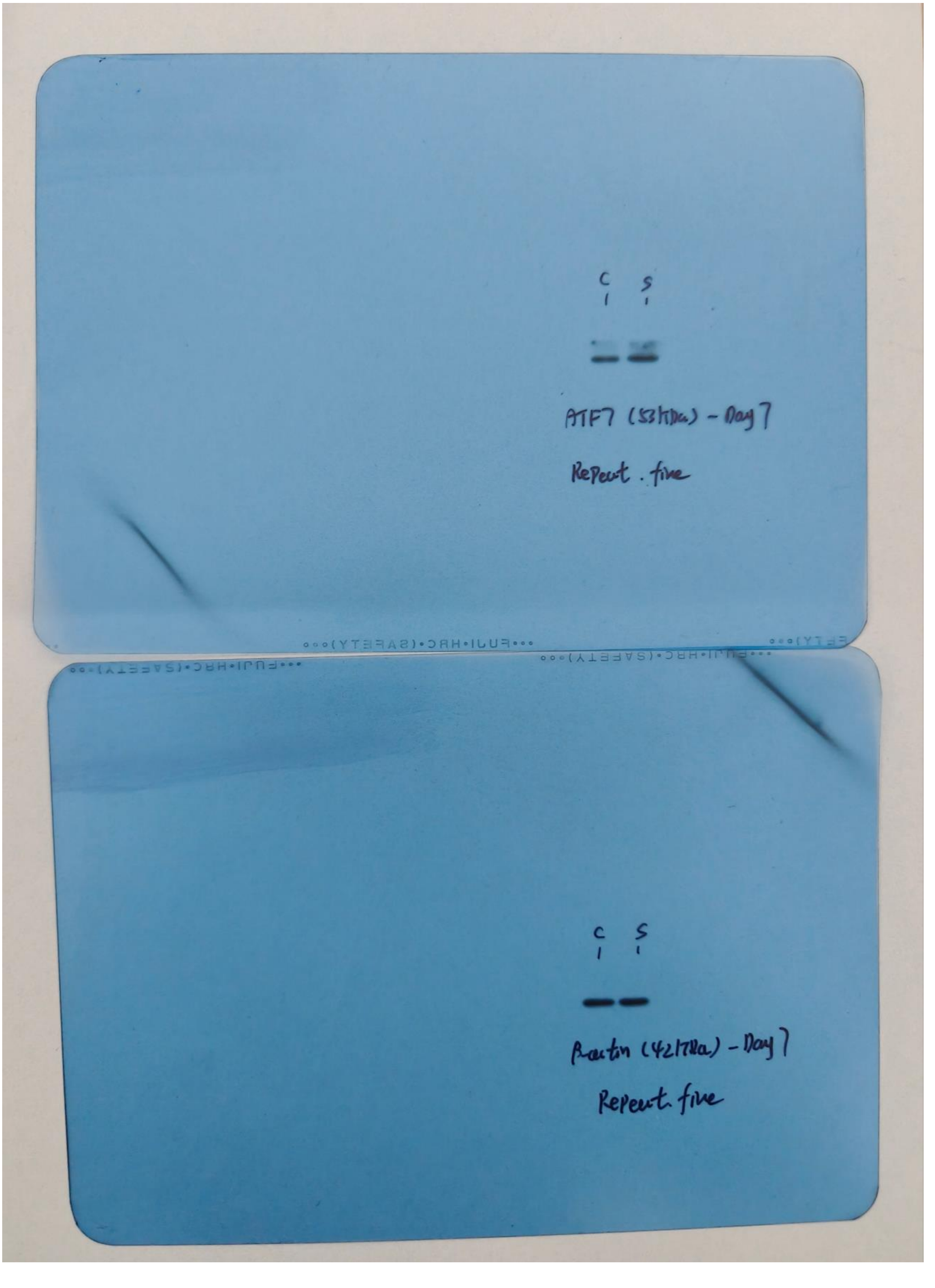

Full unedited gel for Figure 1B

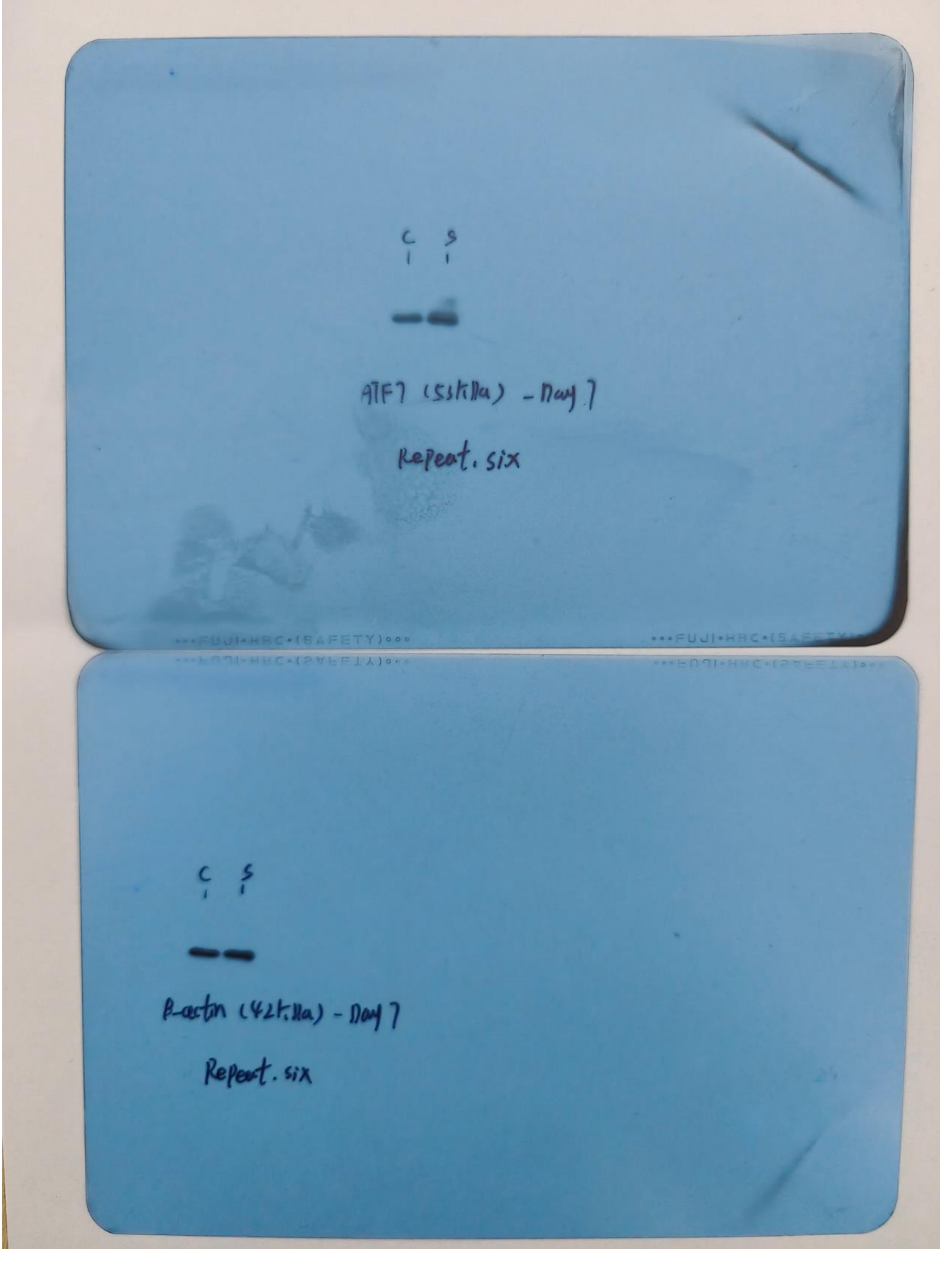

Full unedited gel for Figure 1B

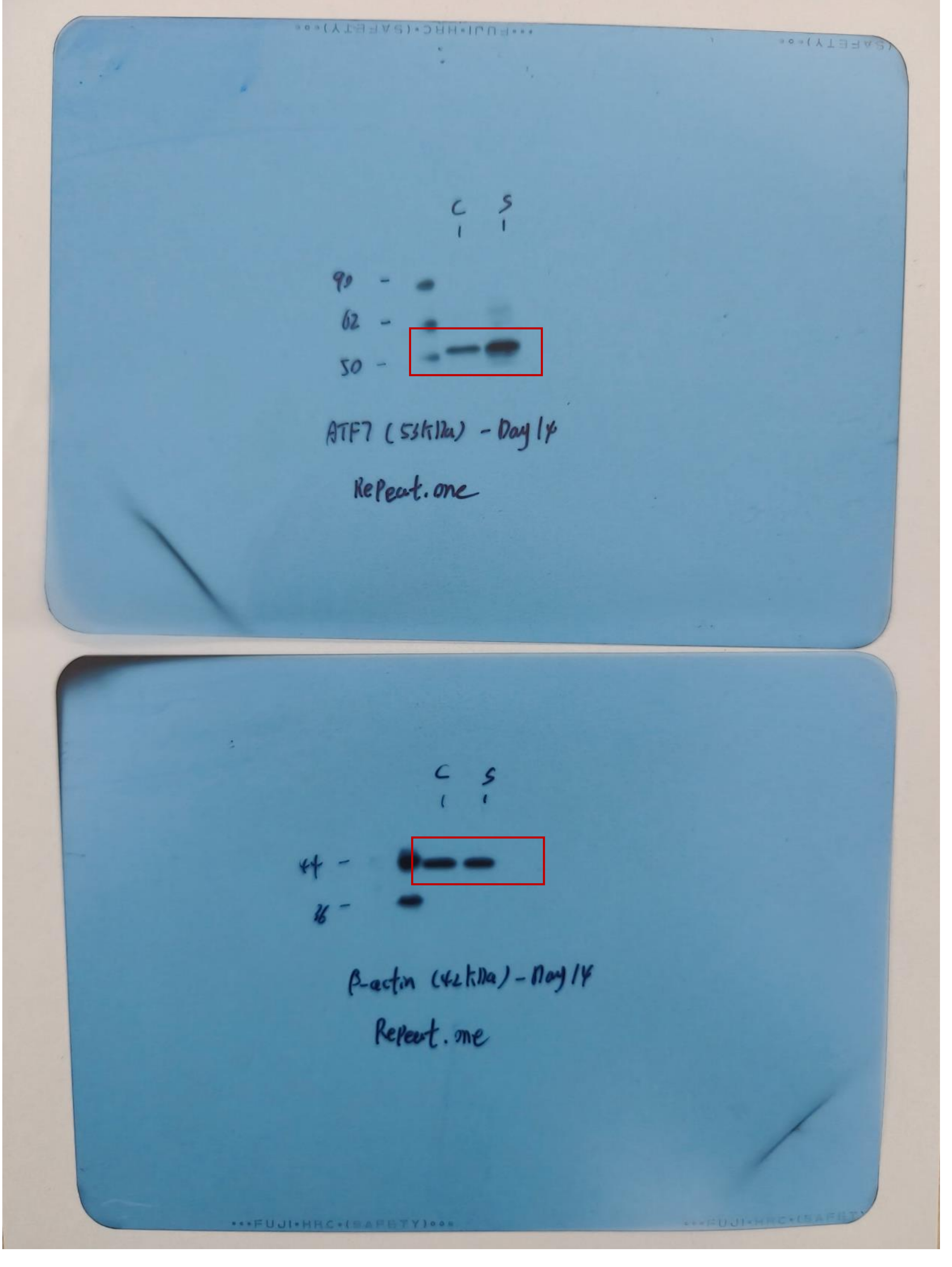

Full unedited gel for Figure 1B

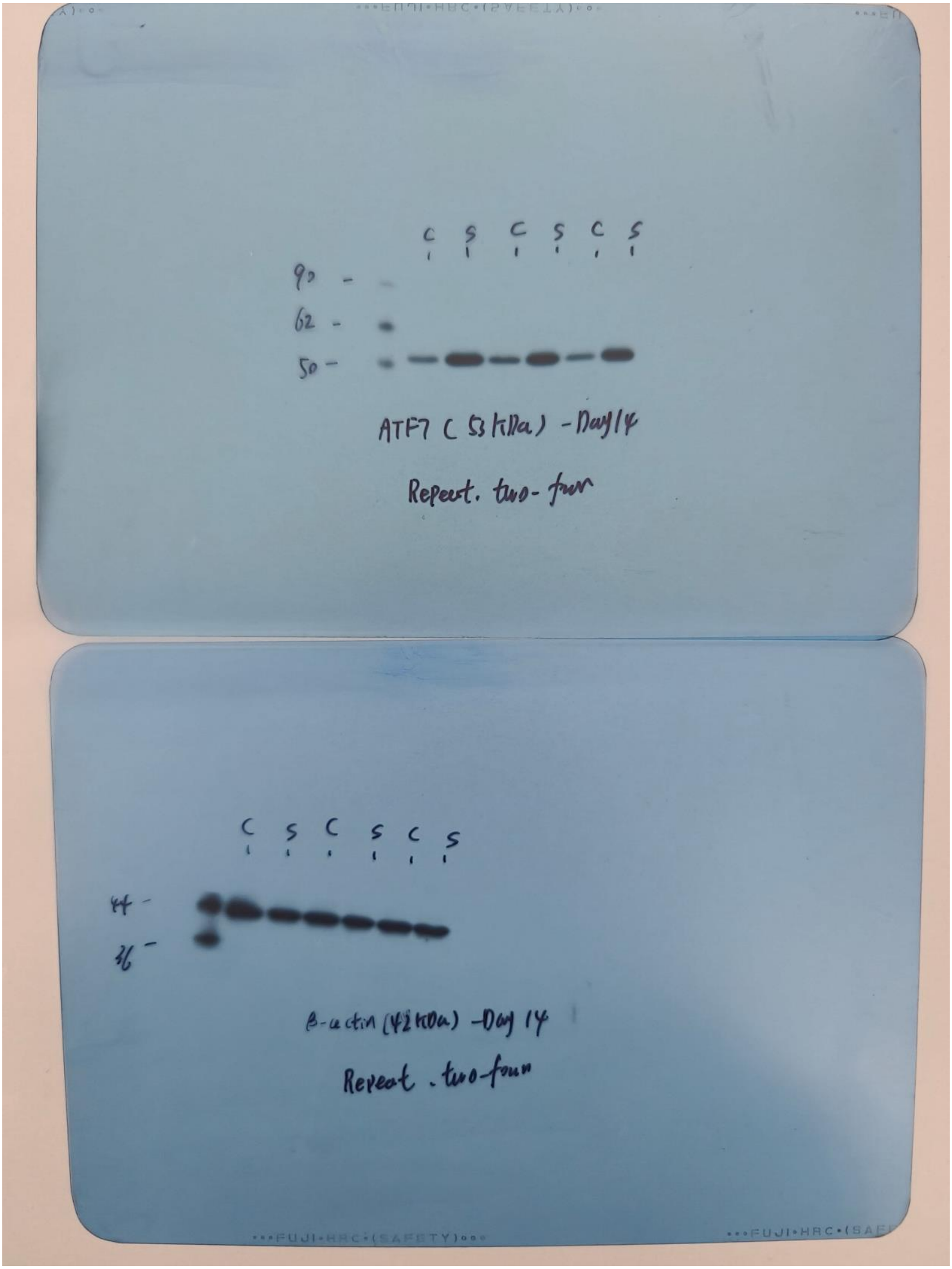

Full unedited gel for Figure 1B

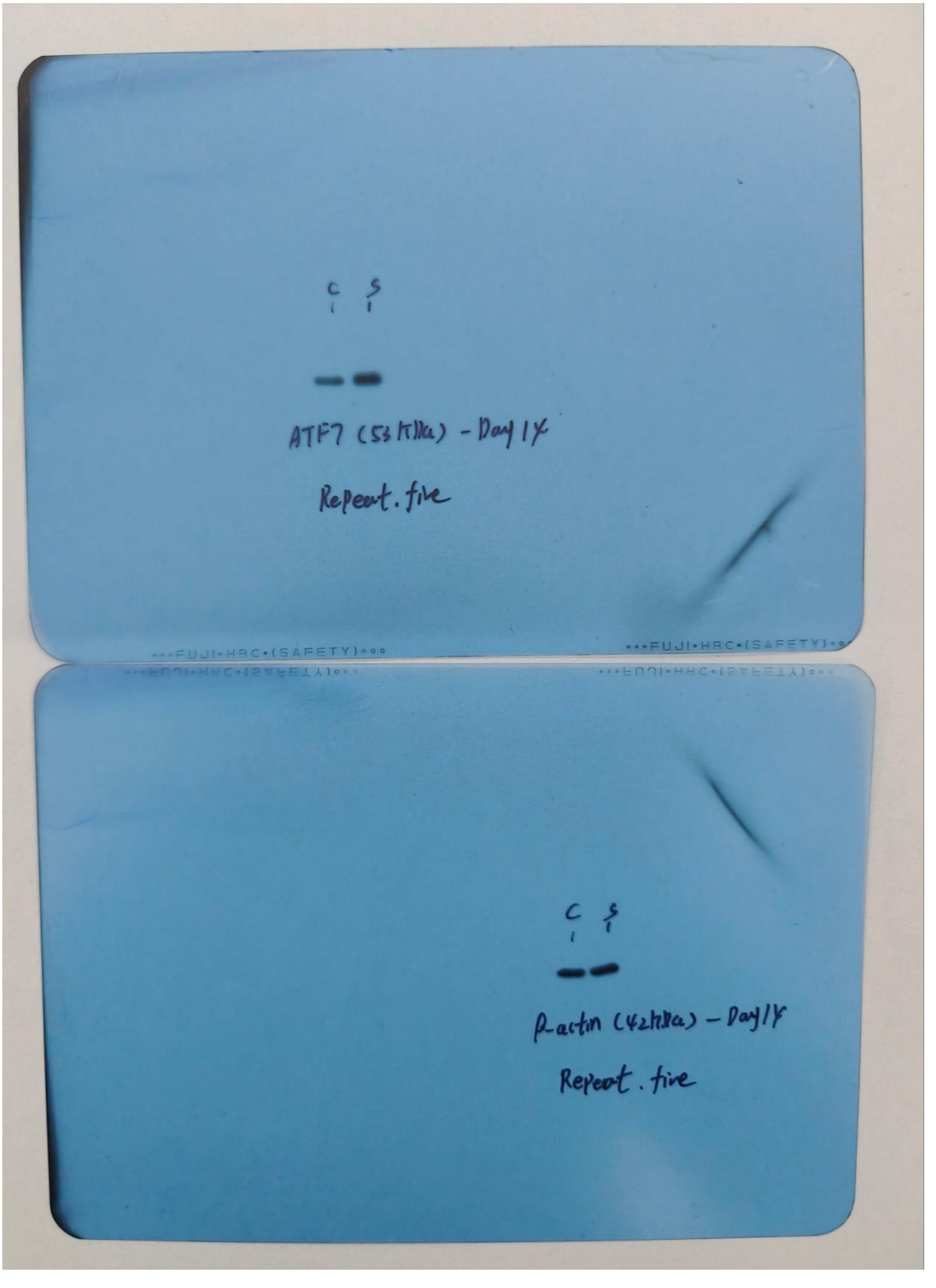

Full unedited gel for Figure 1B

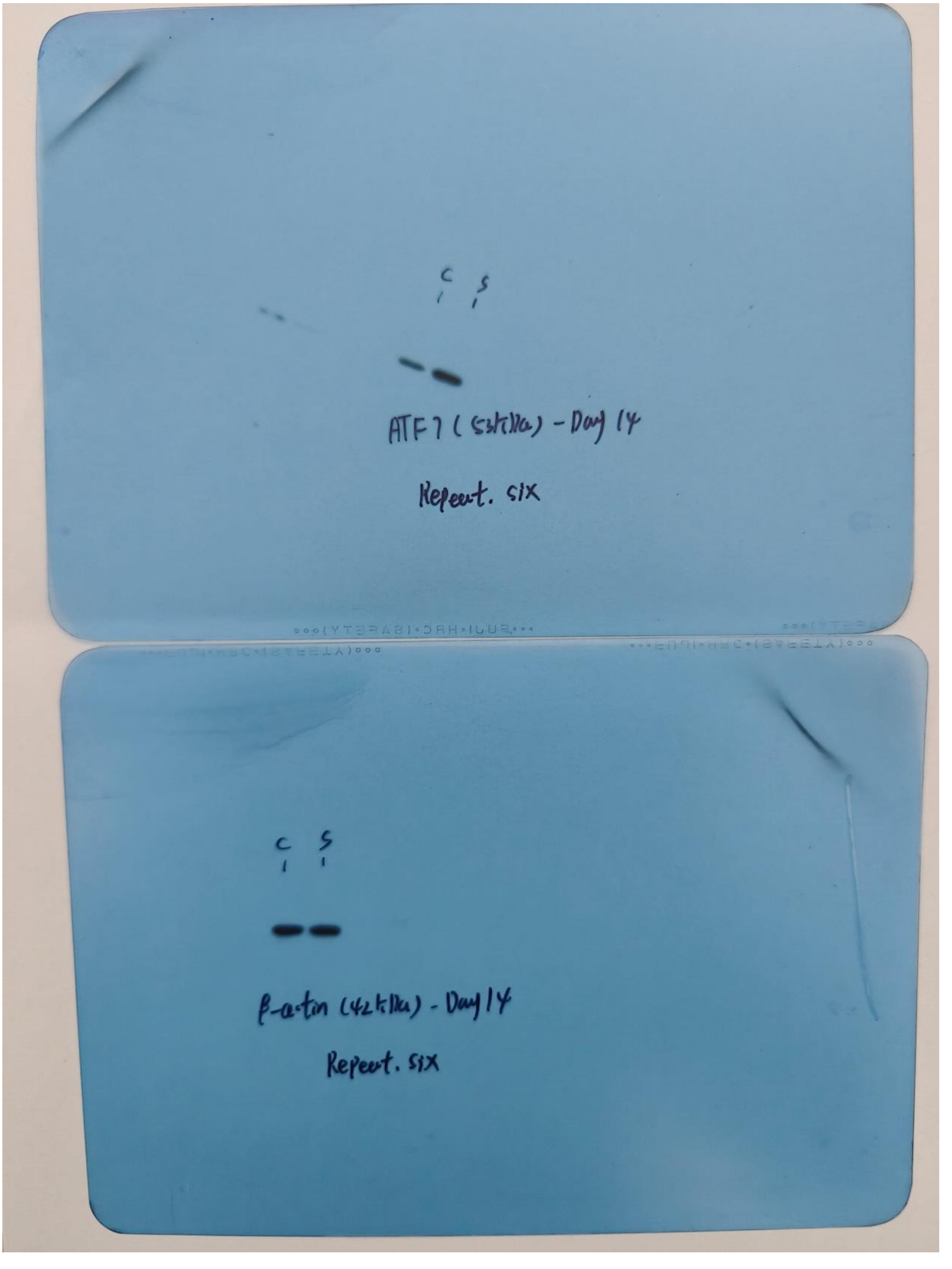

Full unedited gel for Figure 8A

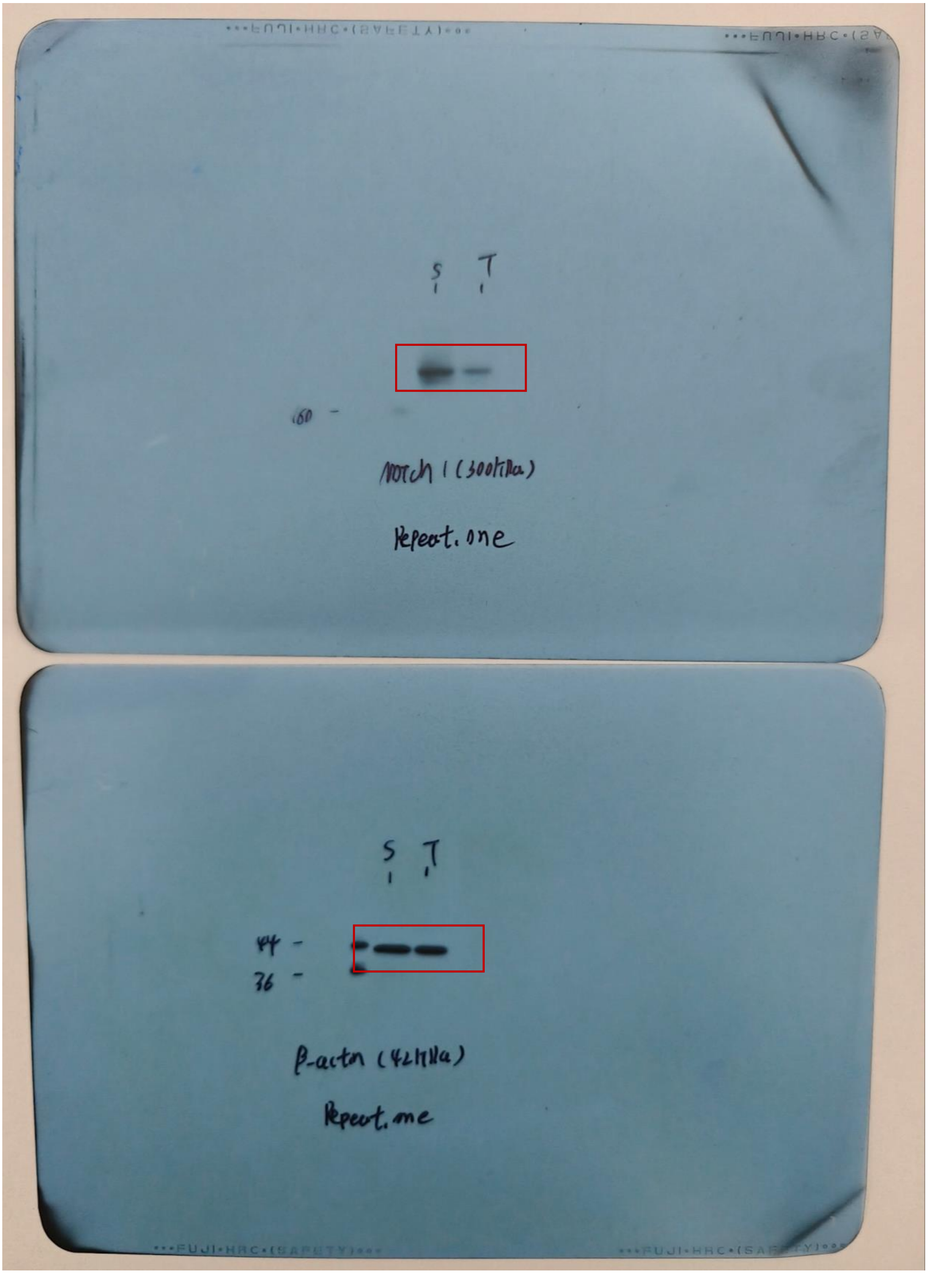

Full unedited gel for Figure 8A

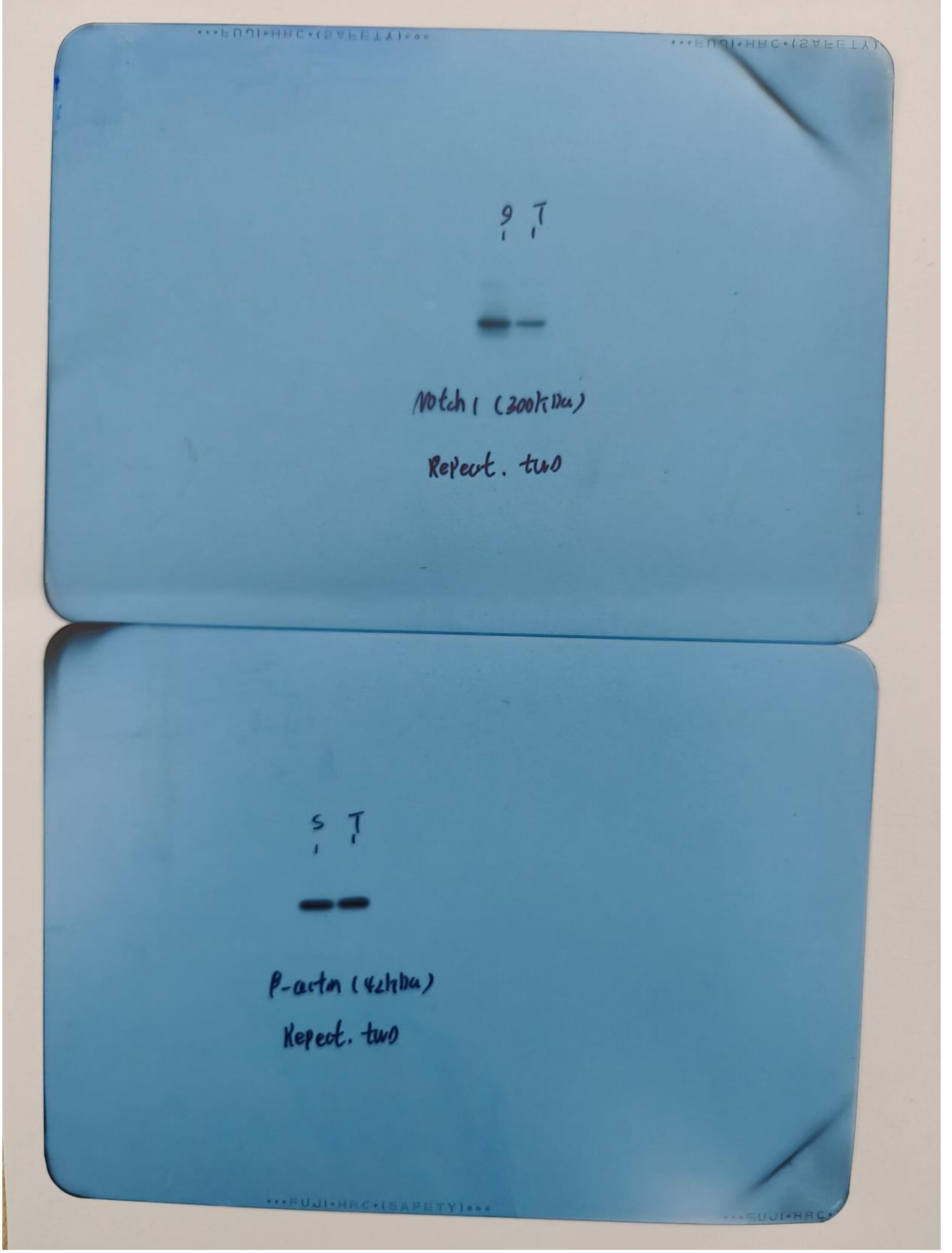

Full unedited gel for Figure 8A

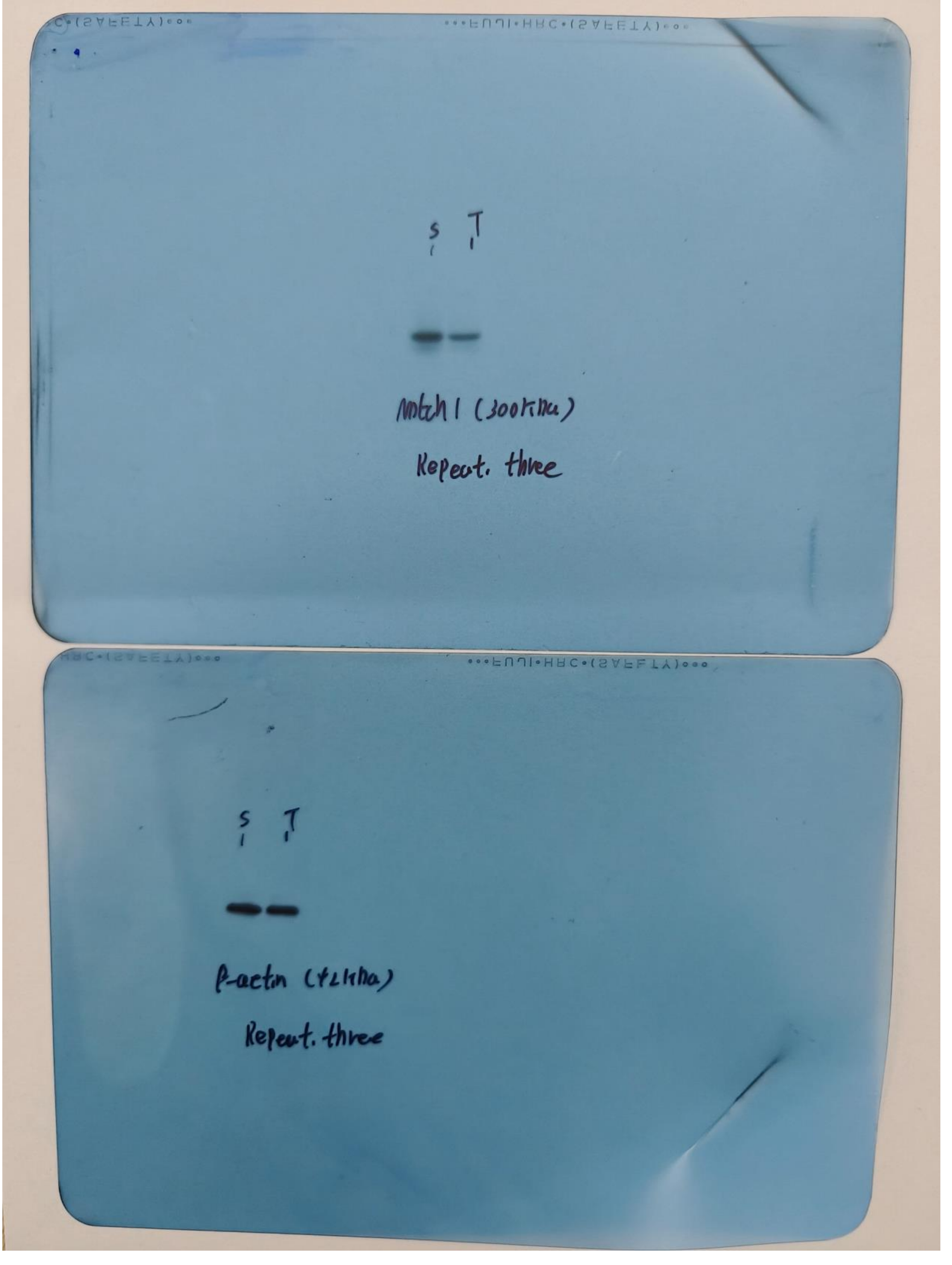

Full unedited gel for Figure 8B

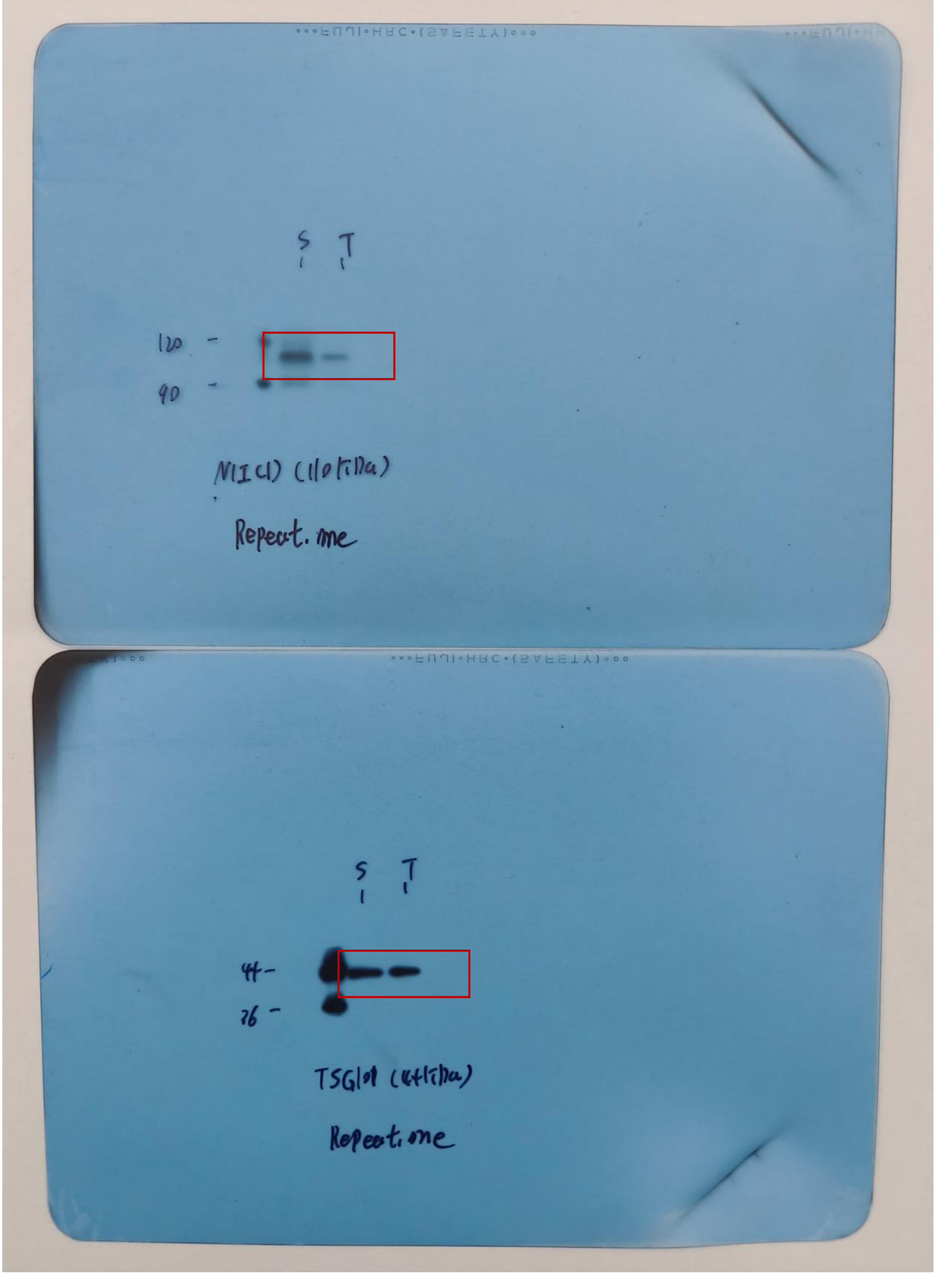

Full unedited gel for Figure 8B

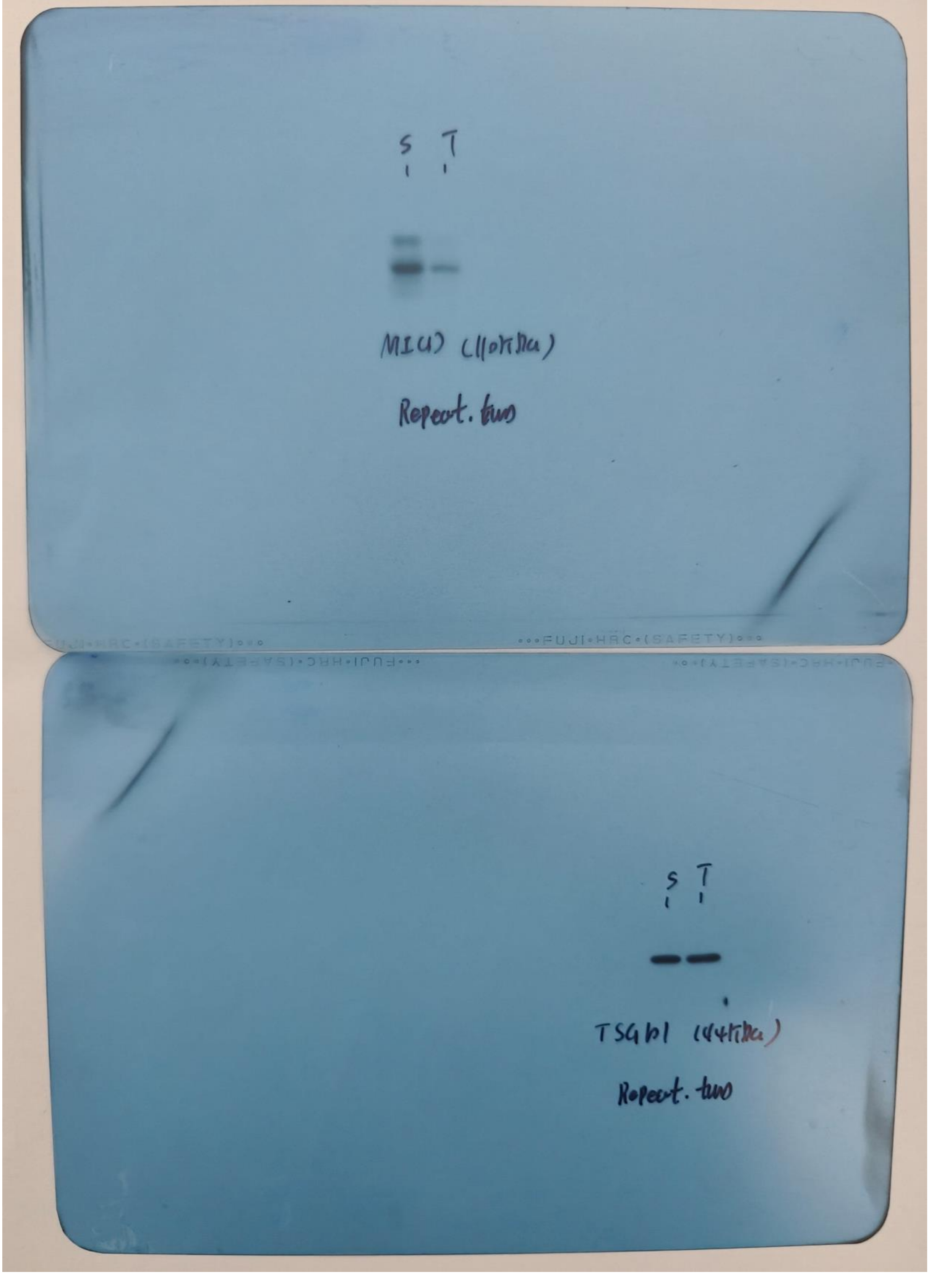

Full unedited gel for Figure 8B

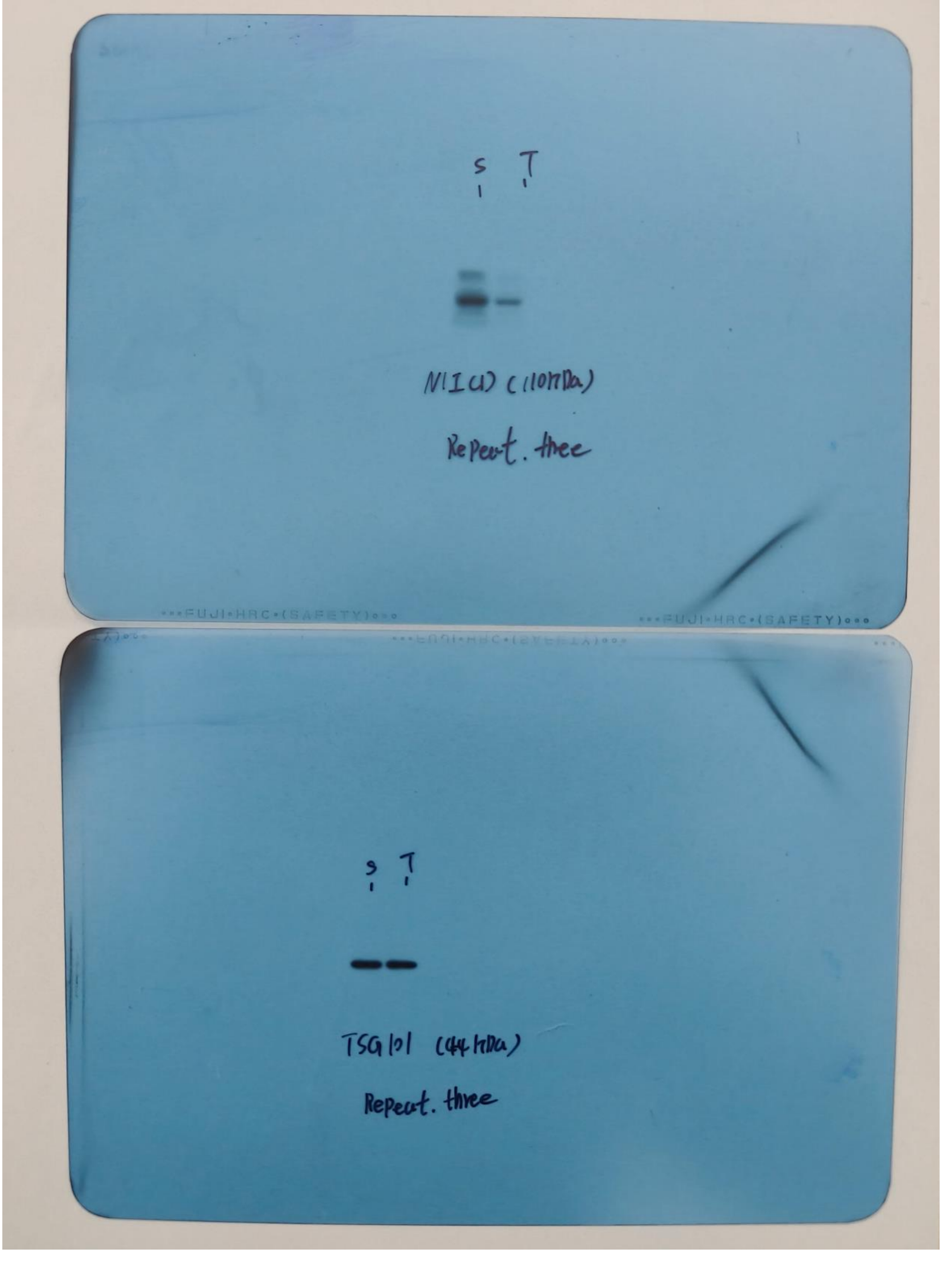

Full unedited gel for Figure 8D

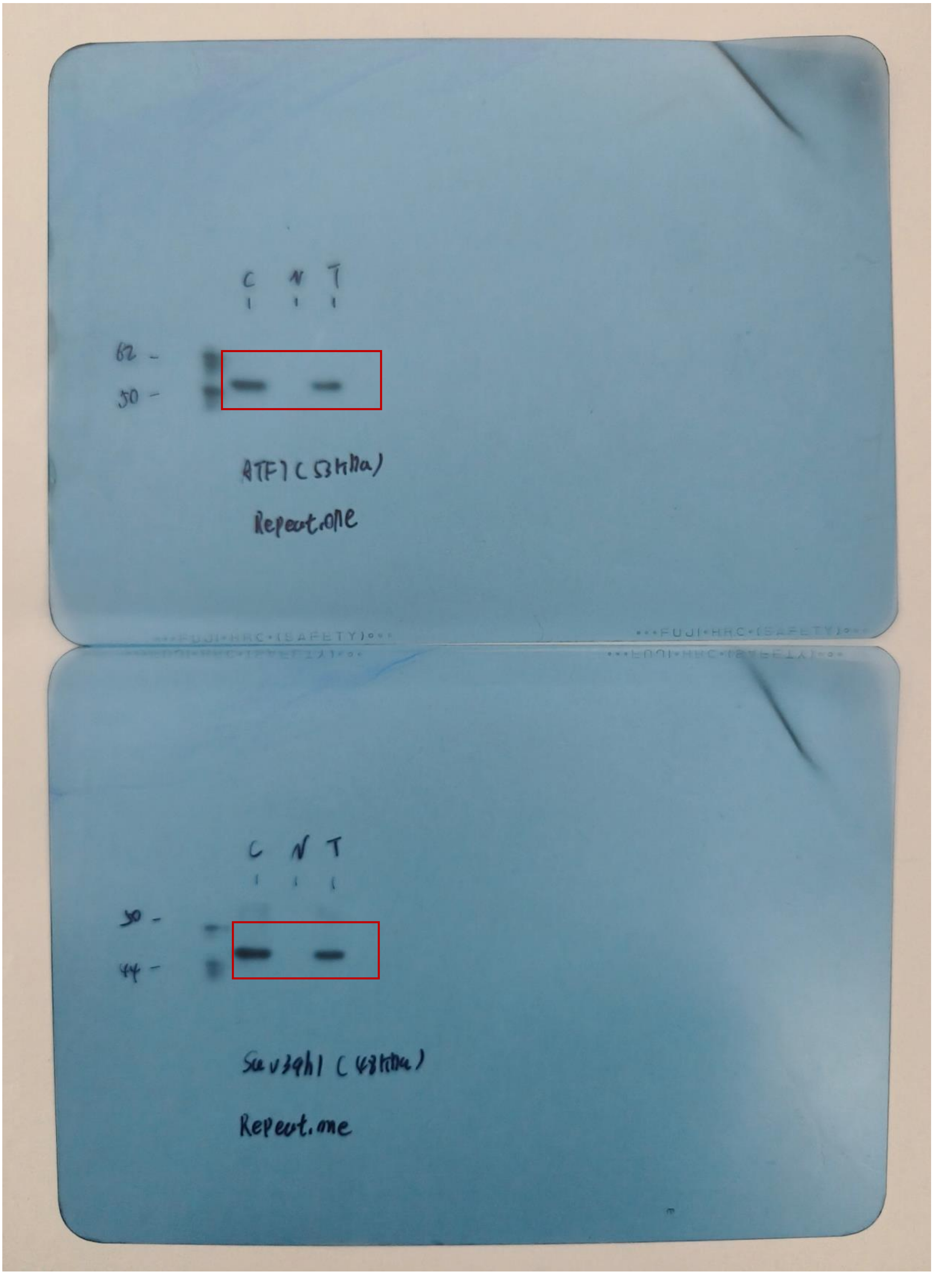

Full unedited gel for Figure 8D

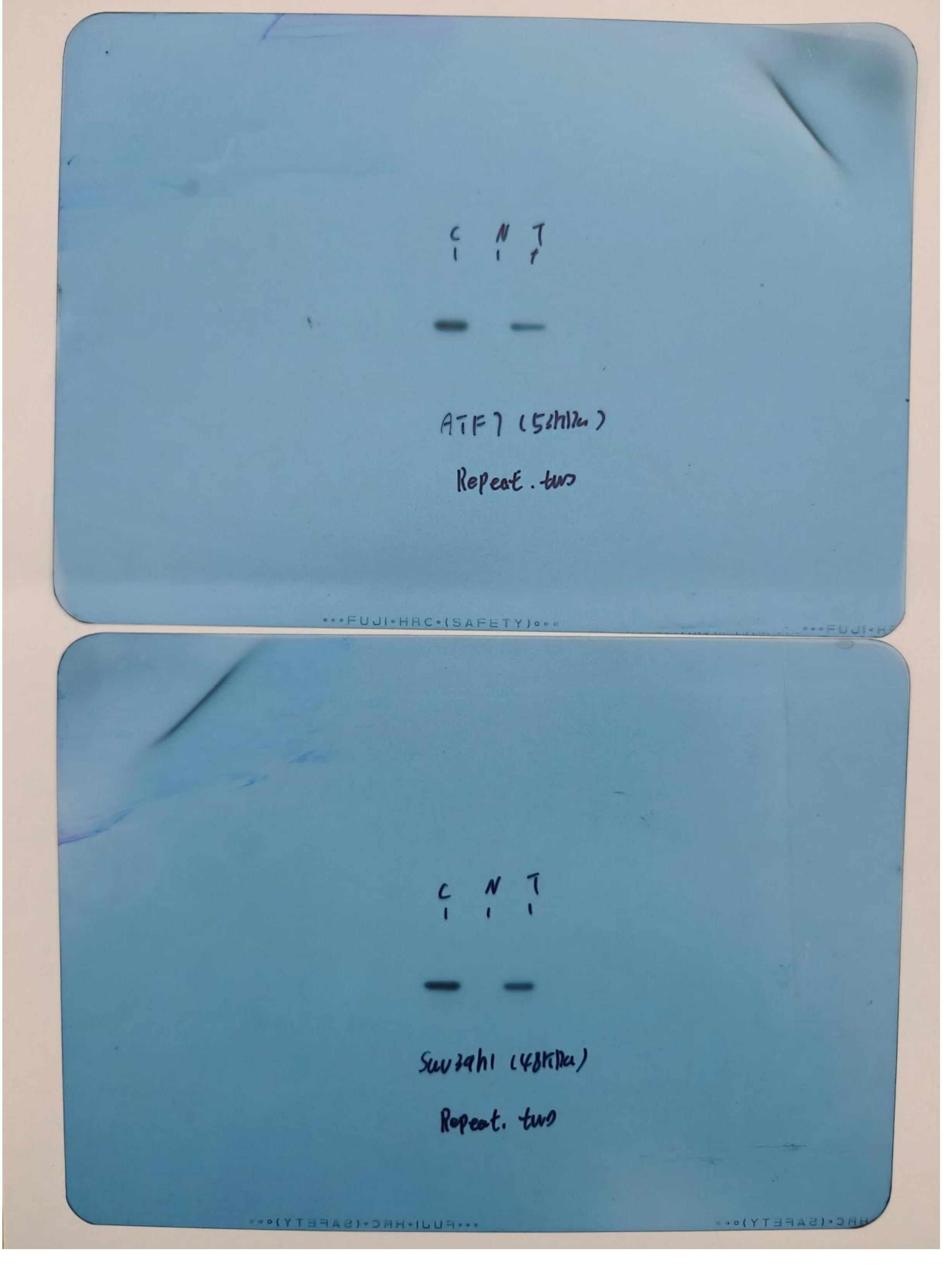

Full unedited gel for Figure 8D

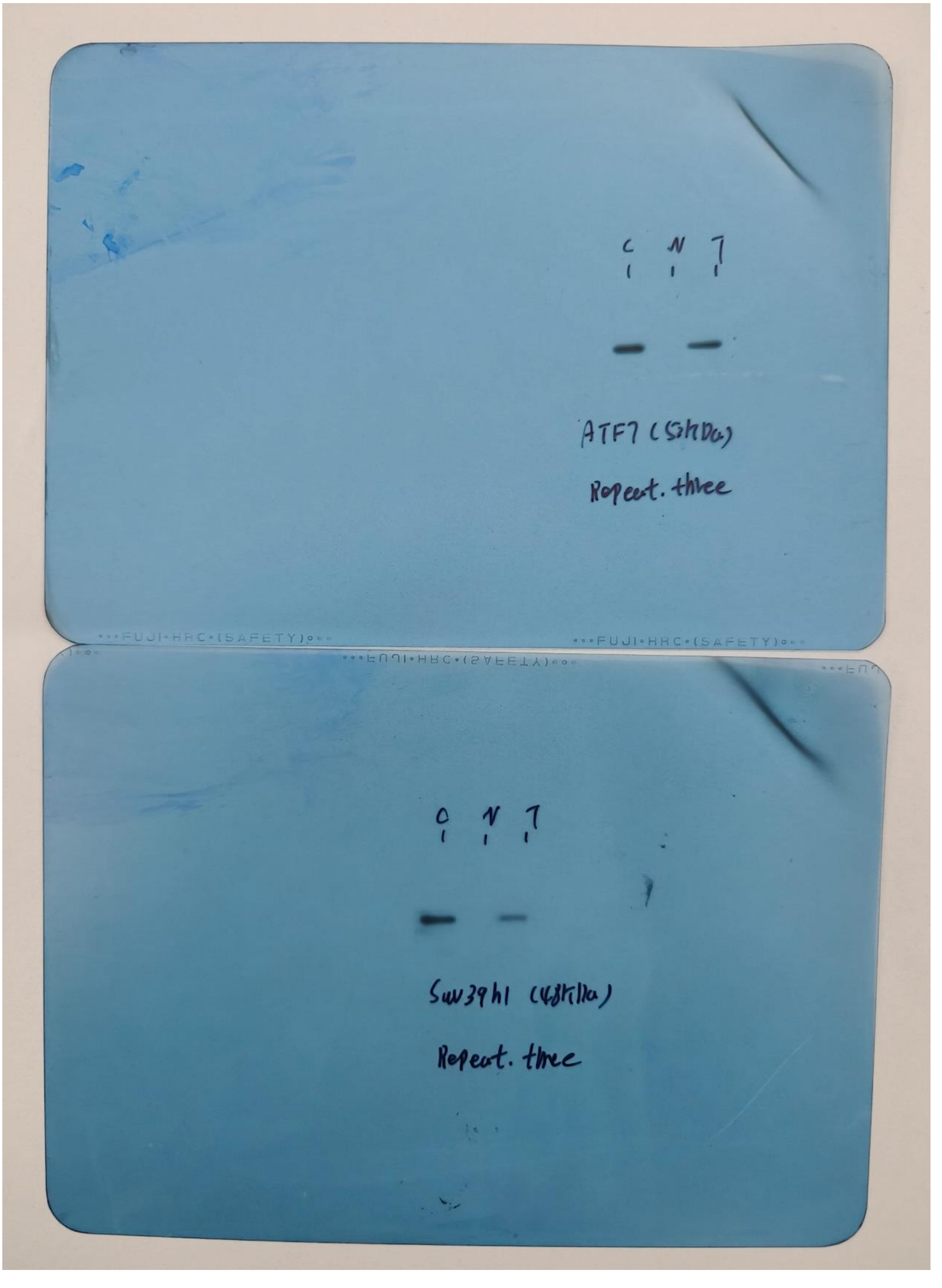

Full unedited gel for Figure 8G

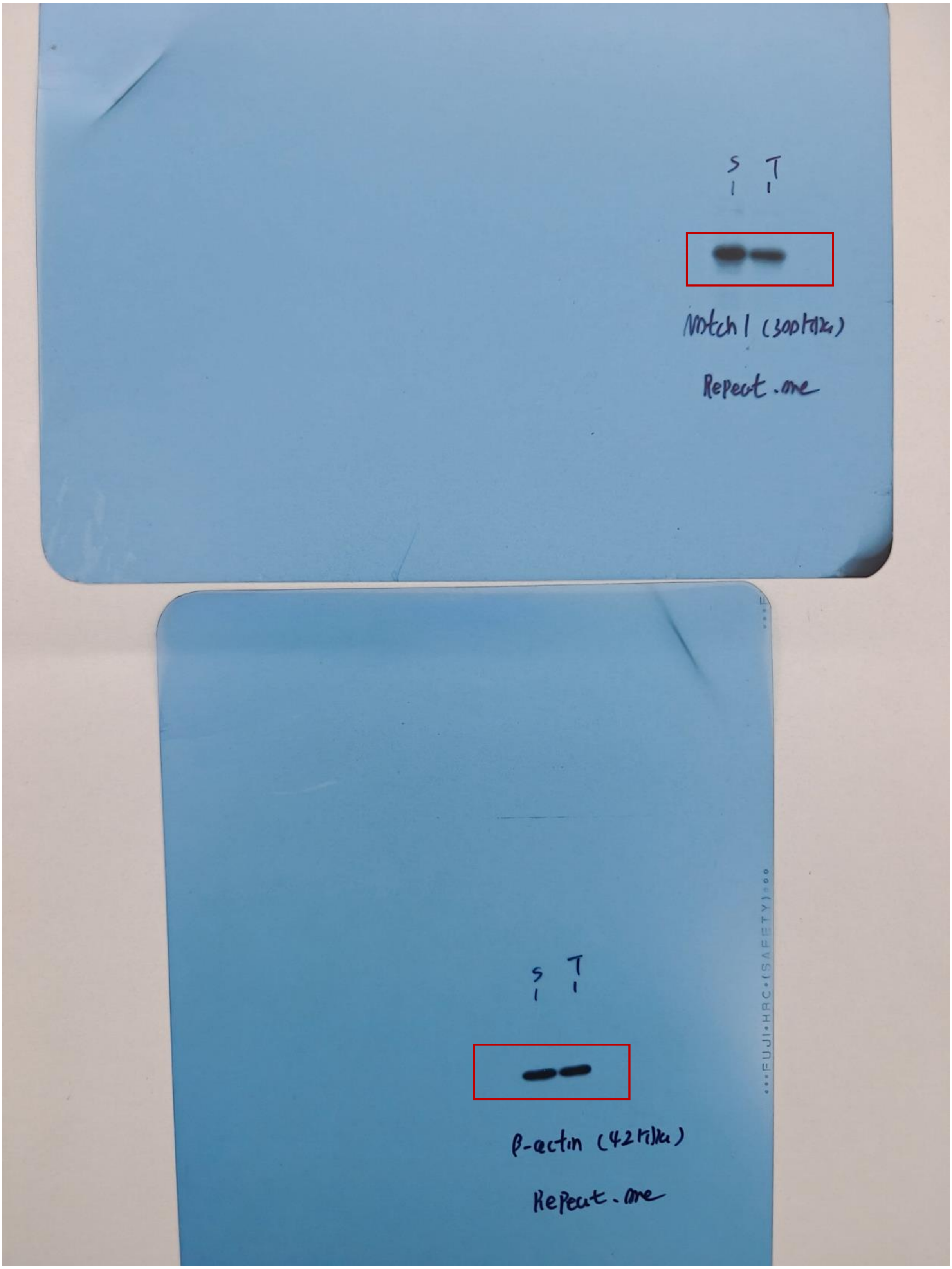

Full unedited gel for Figure 8G

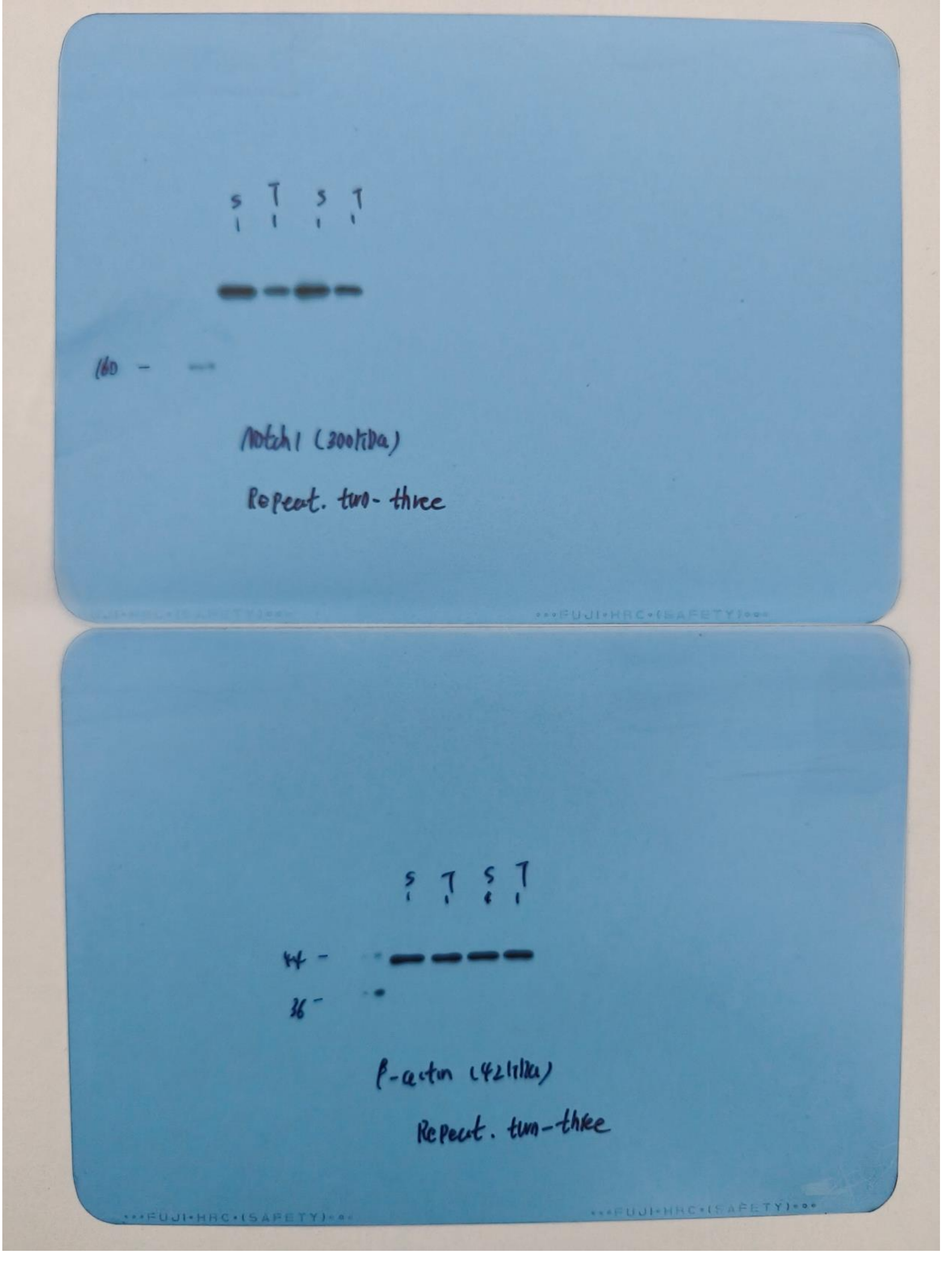

Full unedited gel for Figure 8G

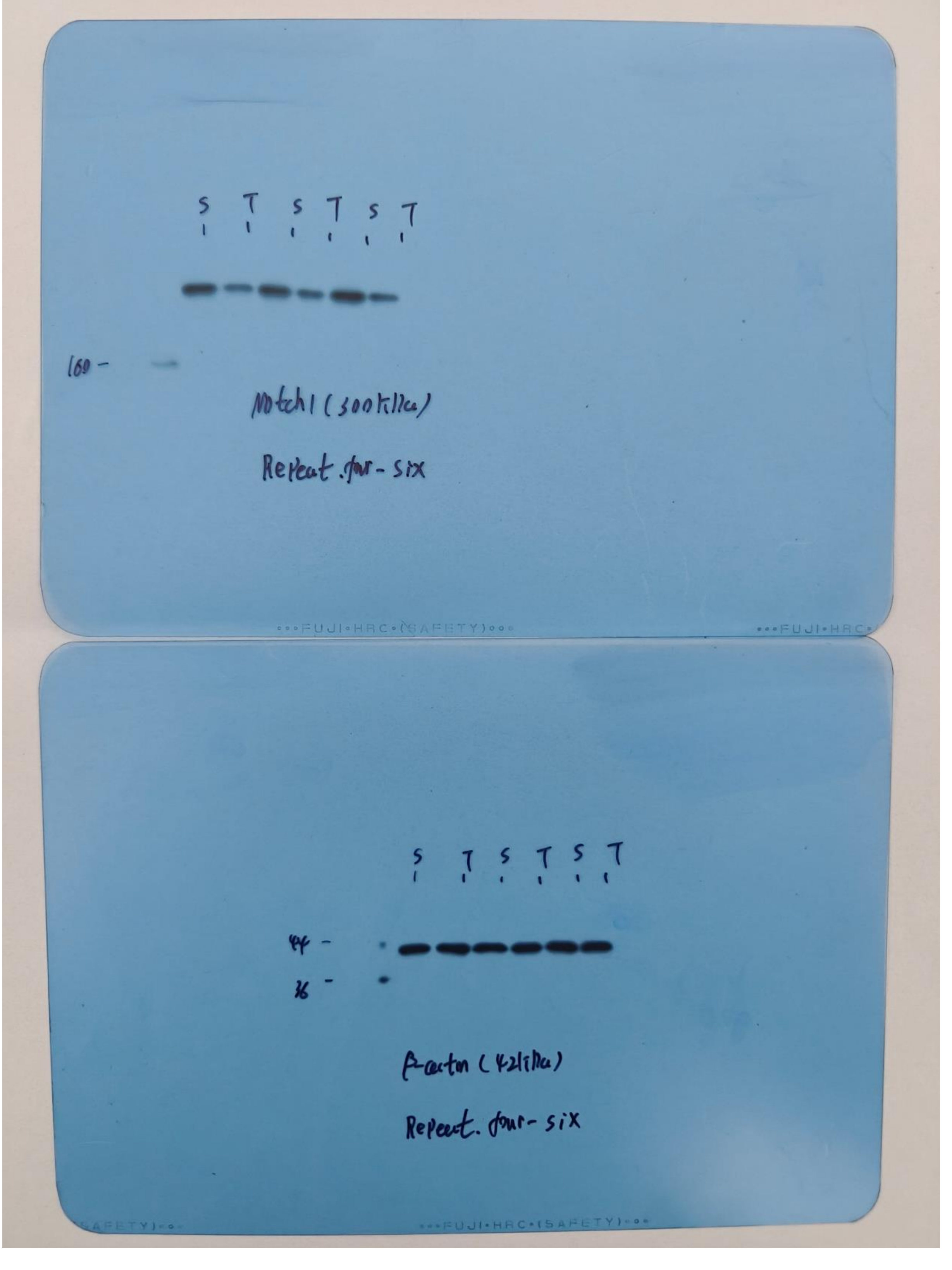

Full unedited gel for Figure 8H

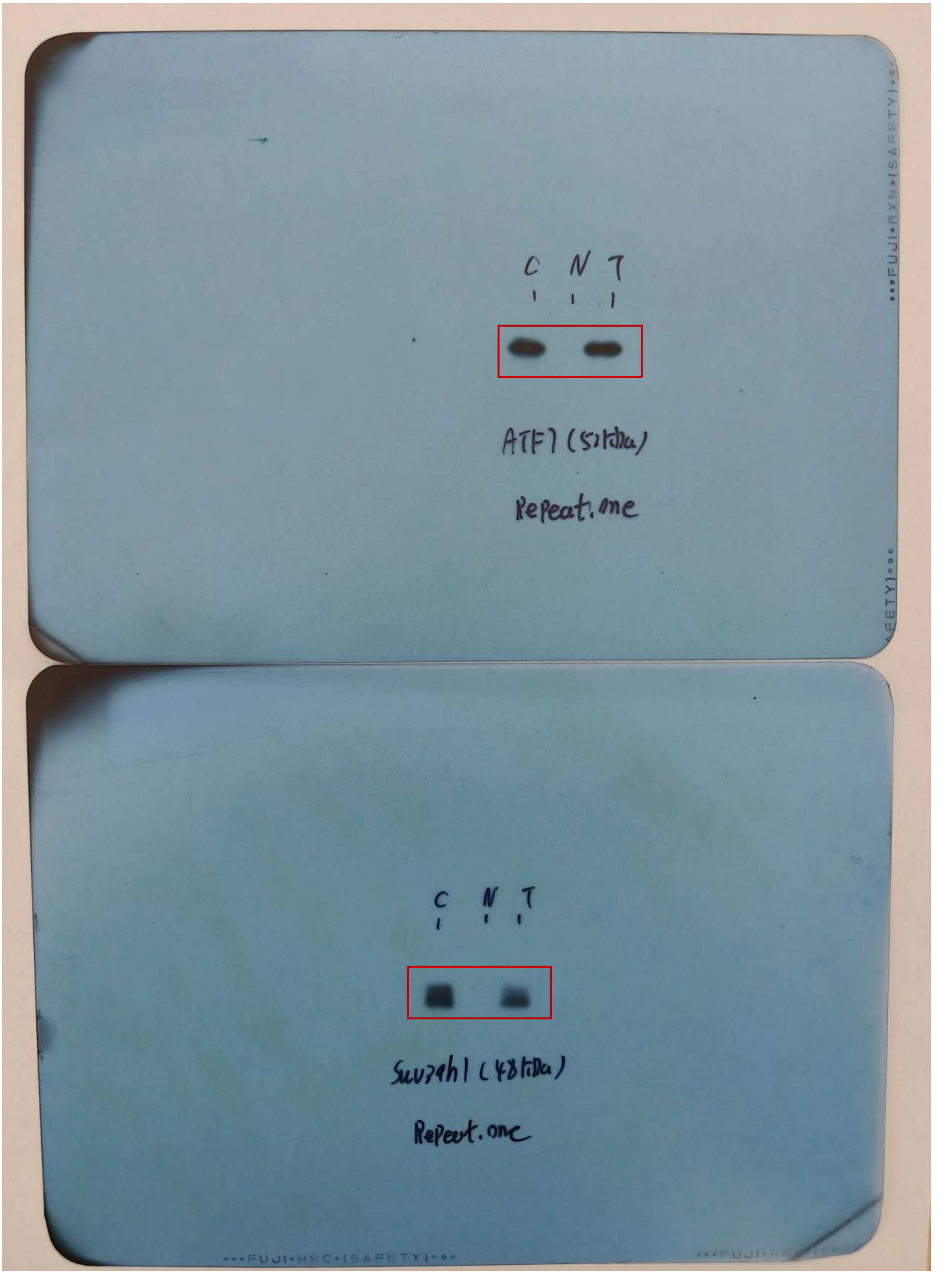

Full unedited gel for Figure 8H

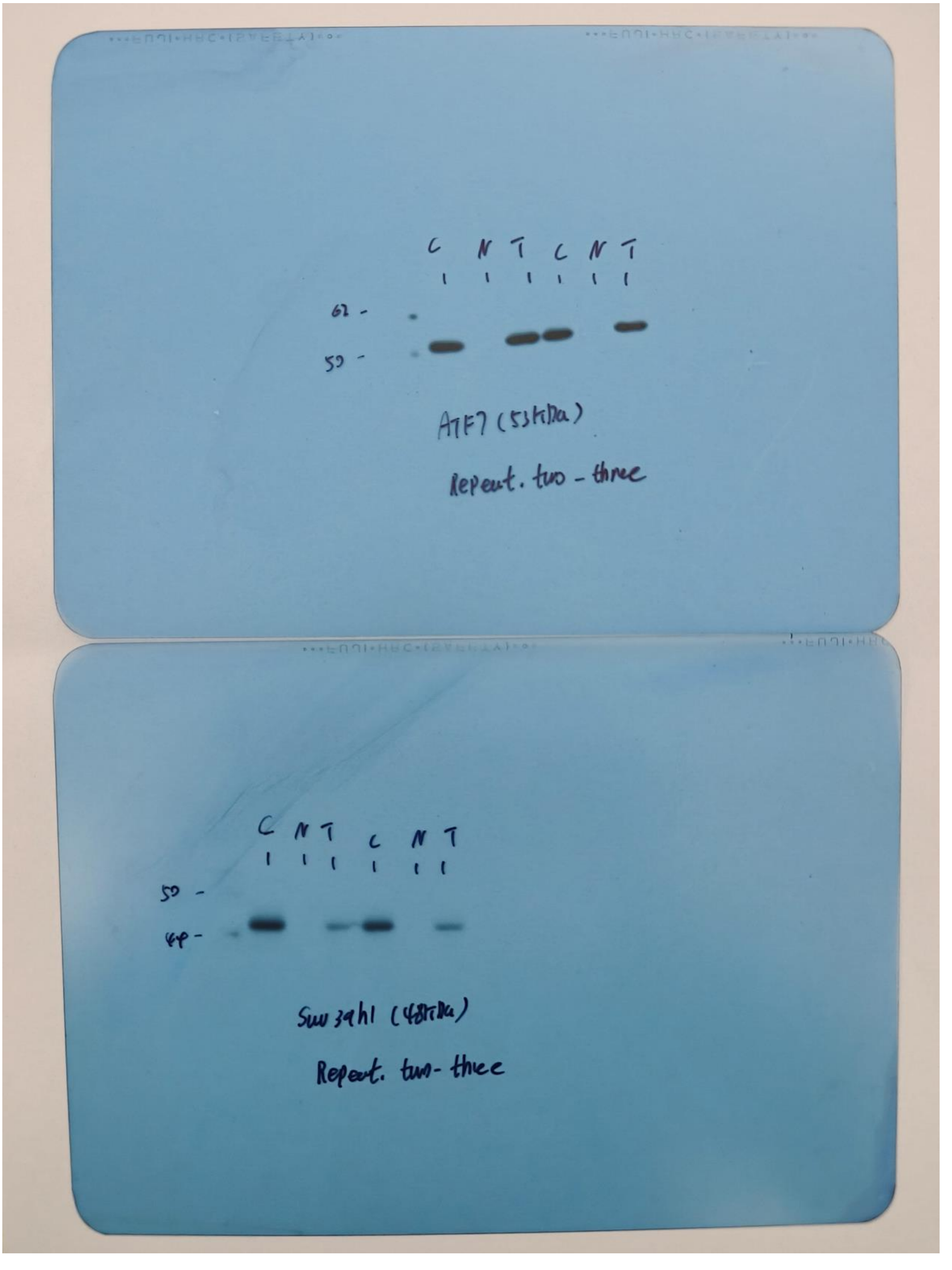

Full unedited gel for Figure 8H

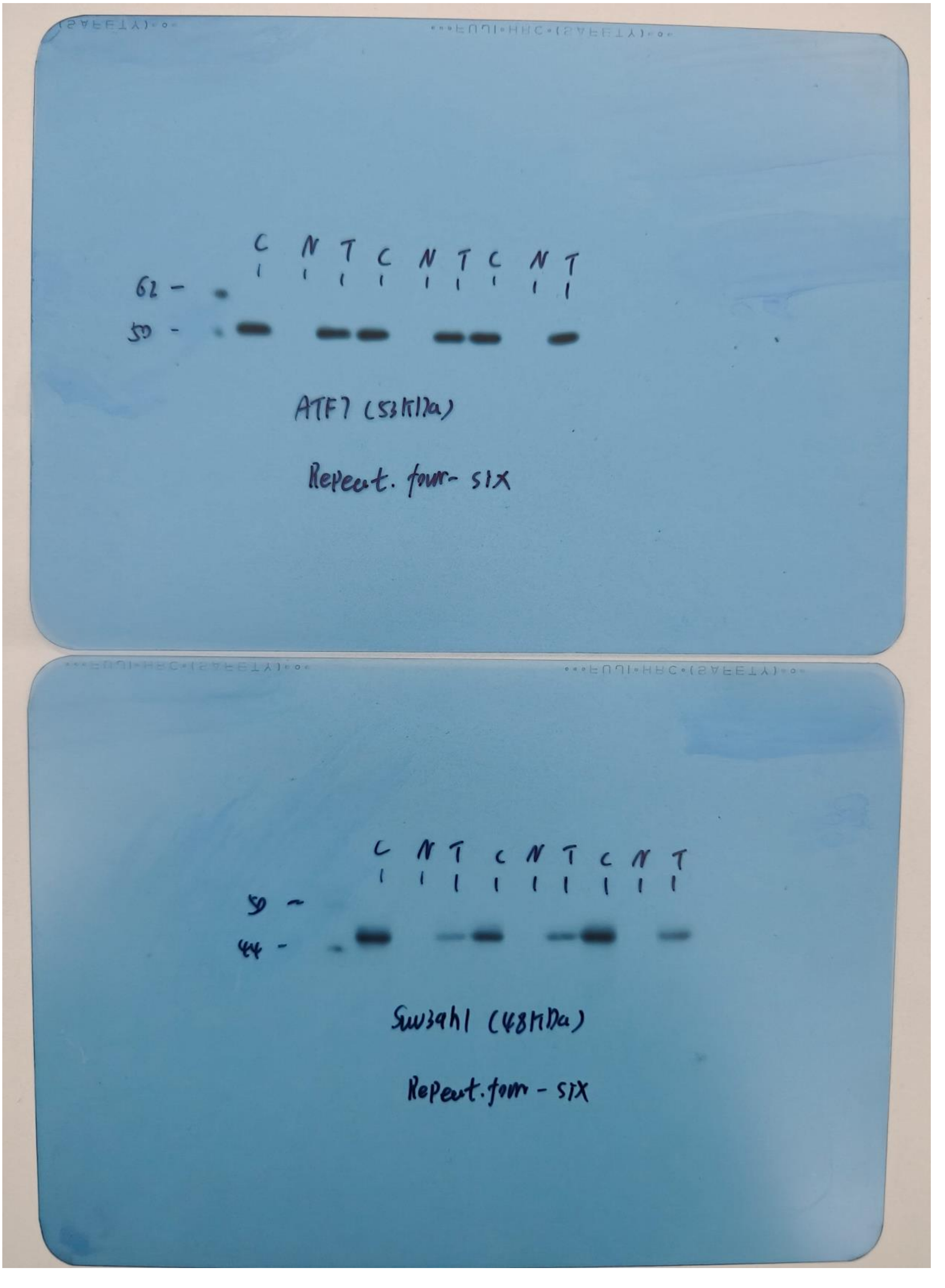

Full unedited gel for Figure 9C

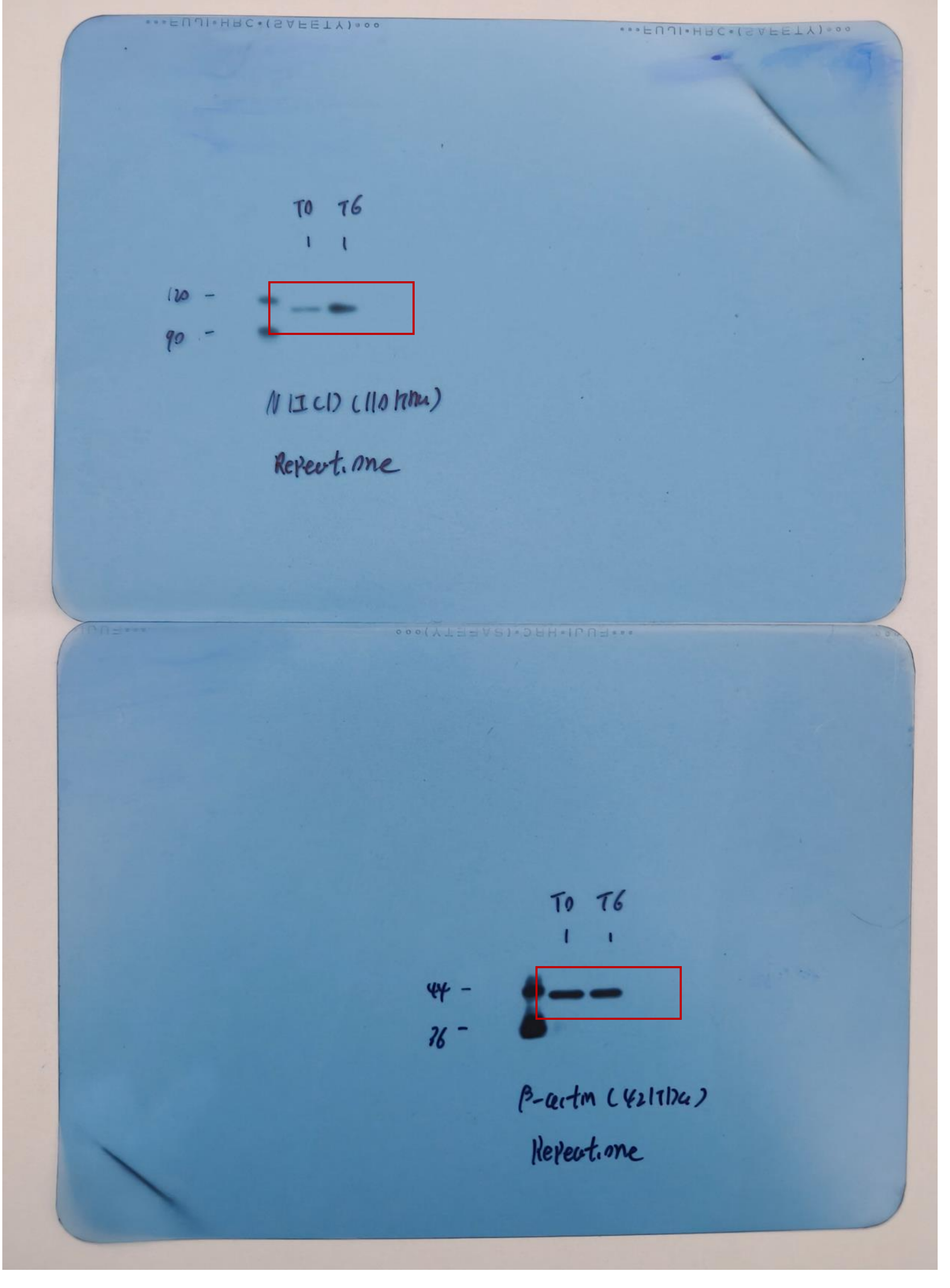

Full unedited gel for Figure 9C

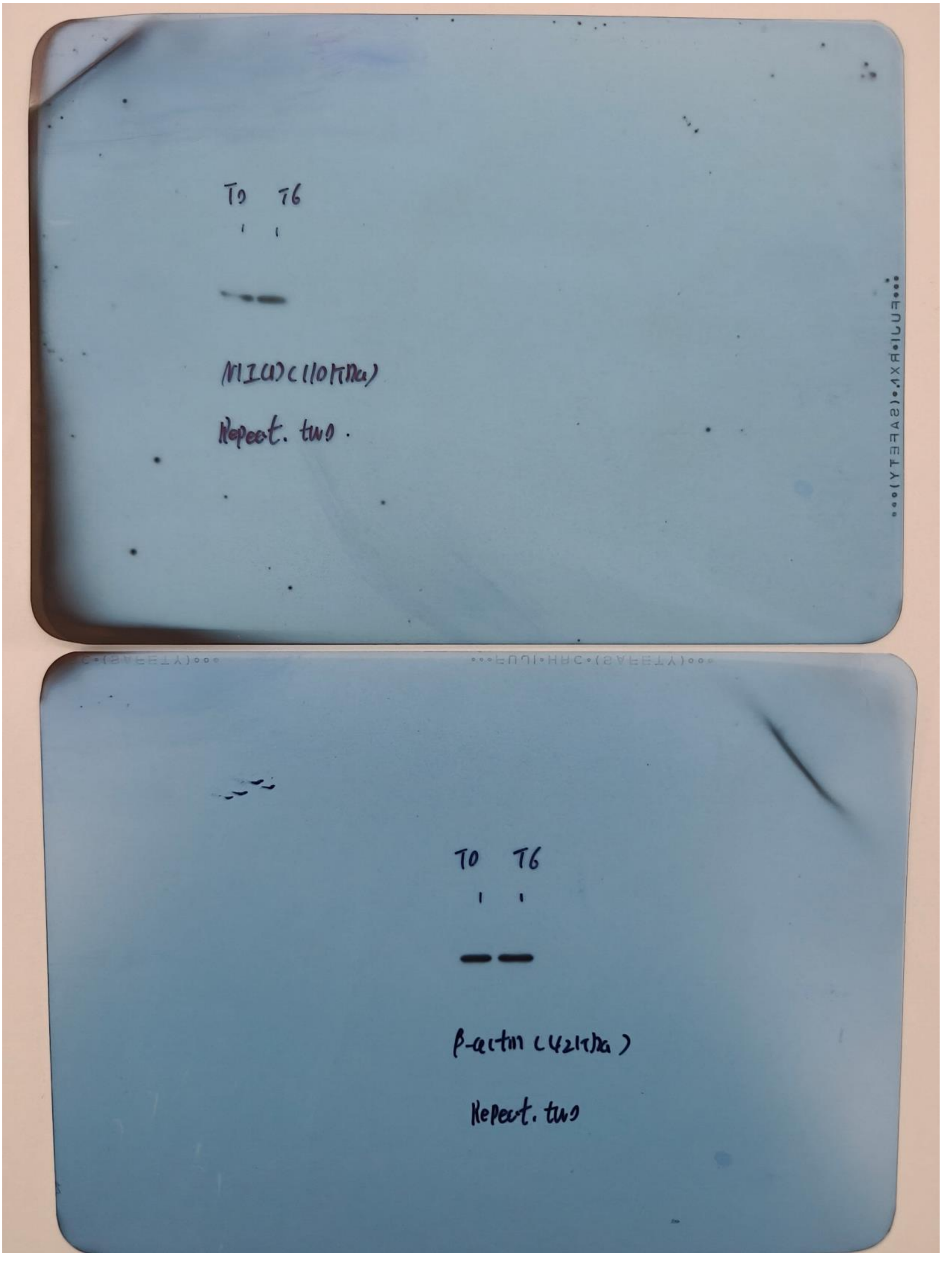

Full unedited gel for Figure 9C

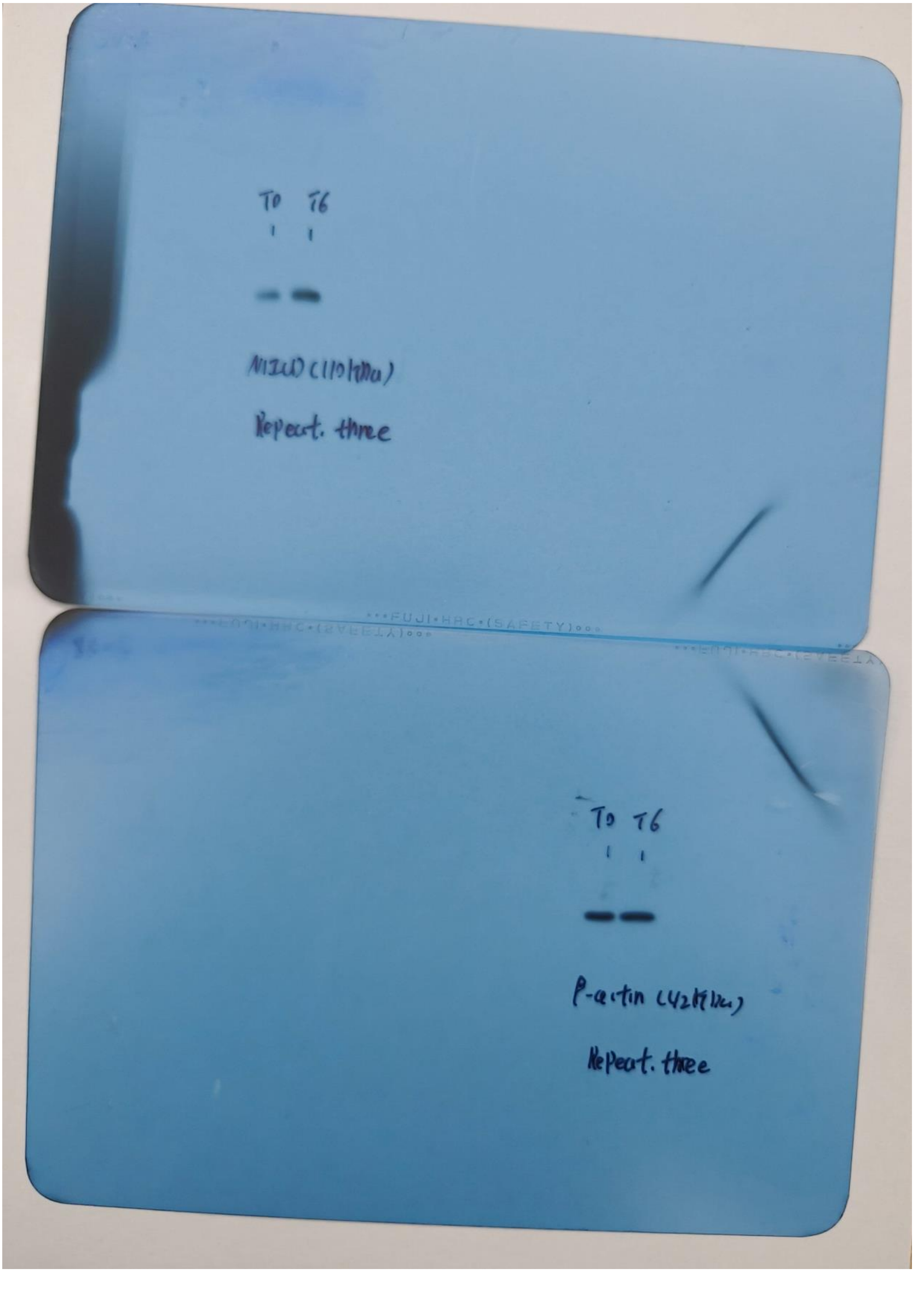

Full unedited gel for Figure 9D

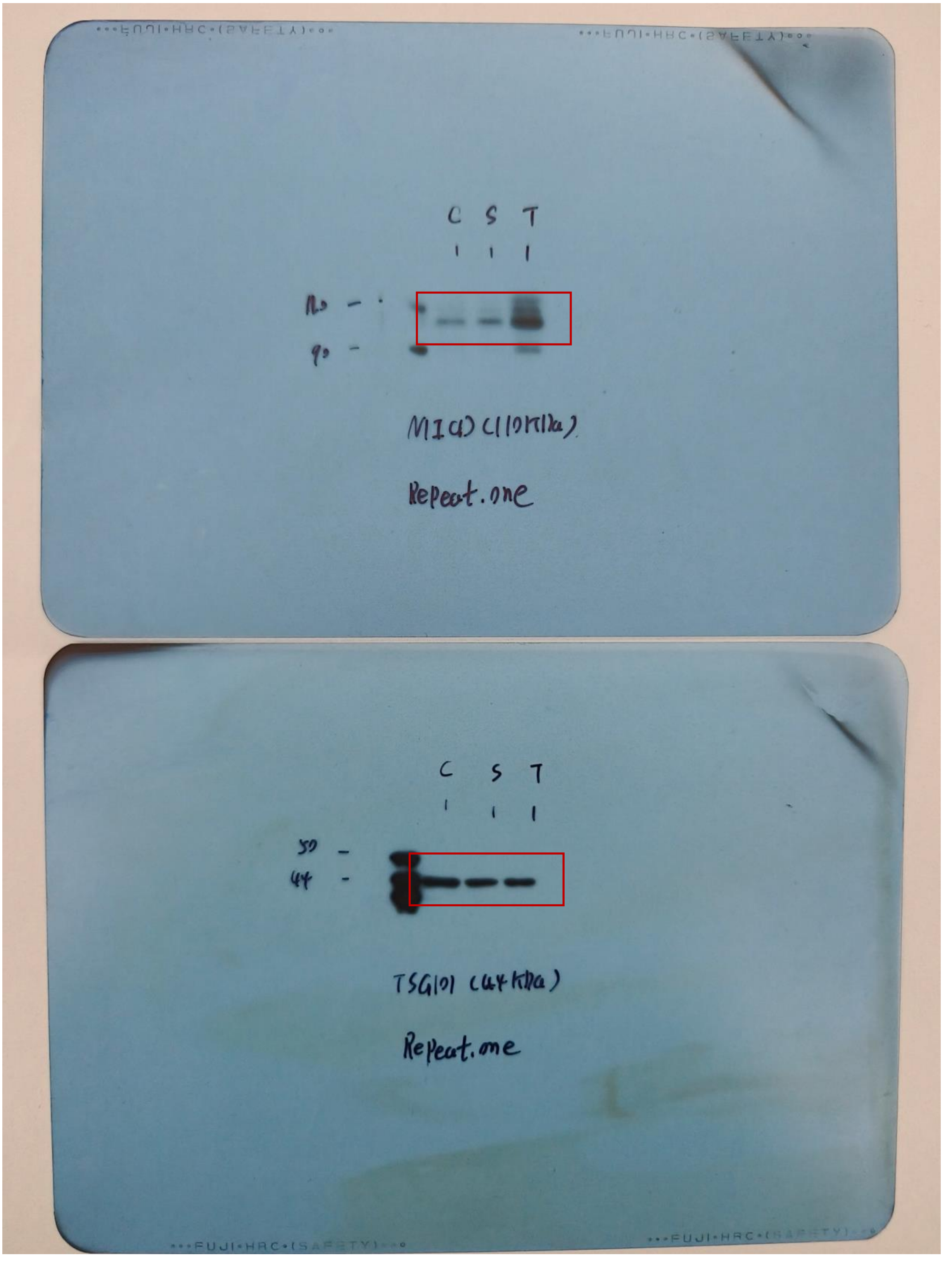

Full unedited gel for Figure 9D

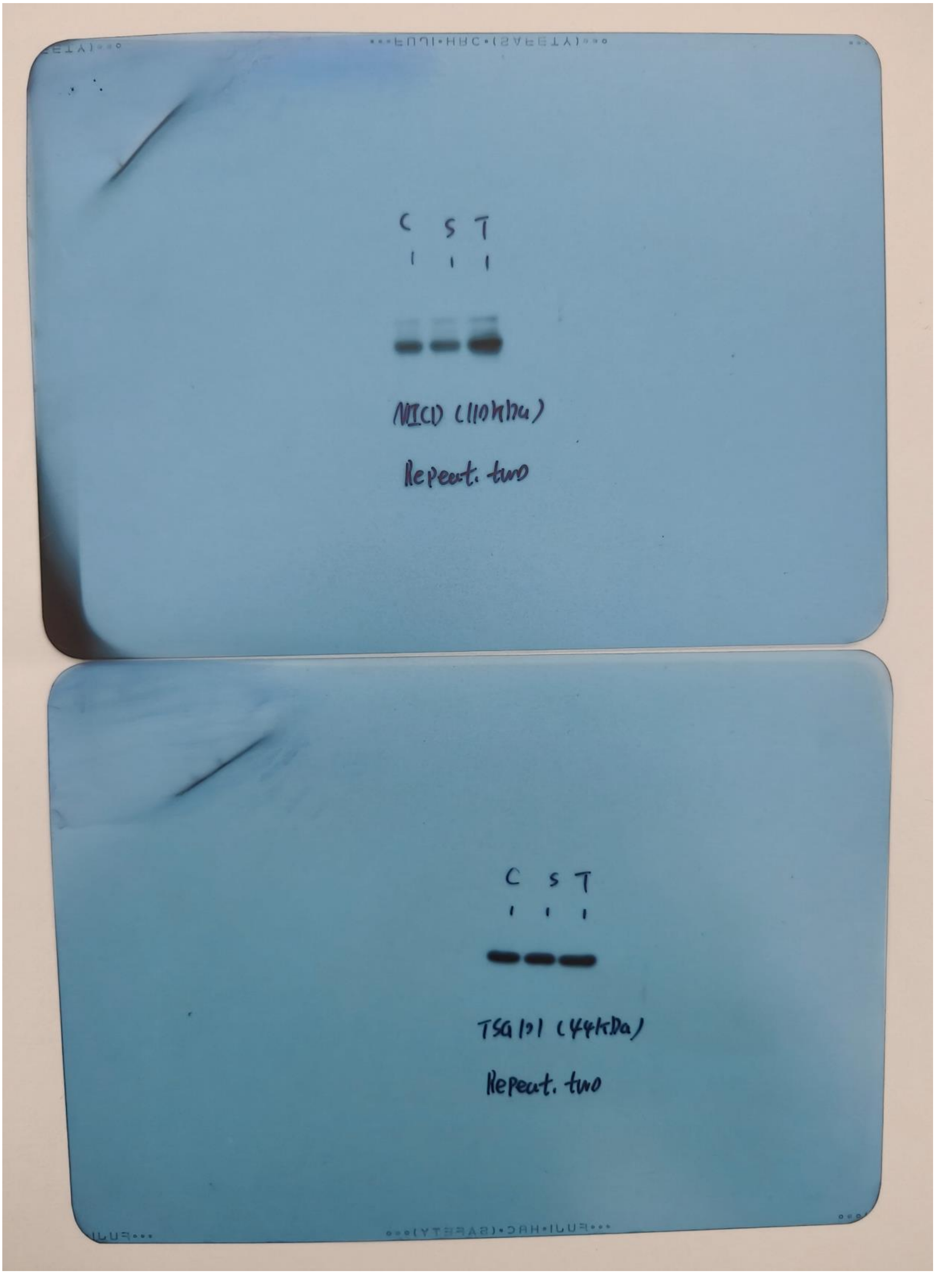

Full unedited gel for Figure 9D

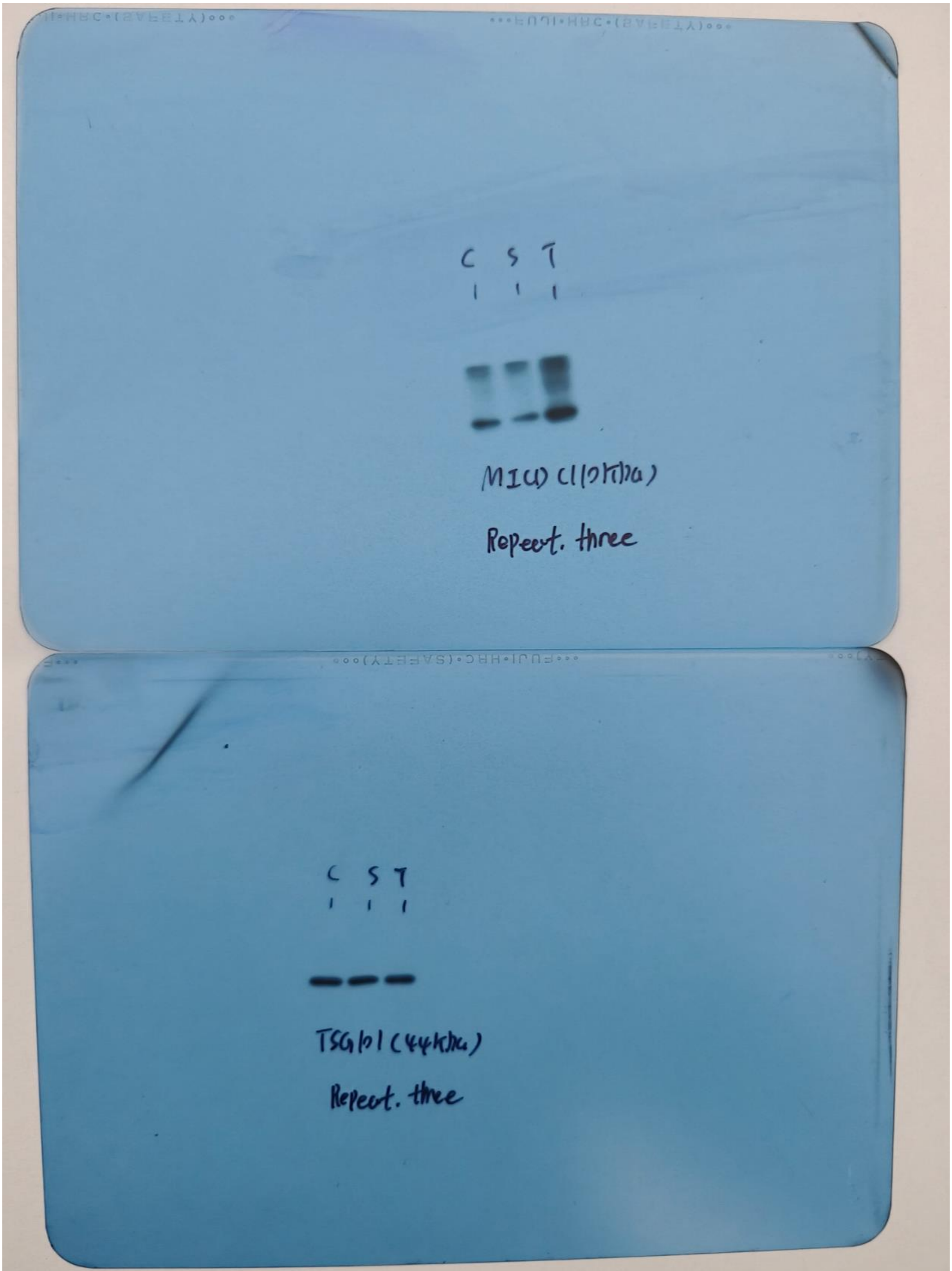

Full unedited gel for Figure 9E

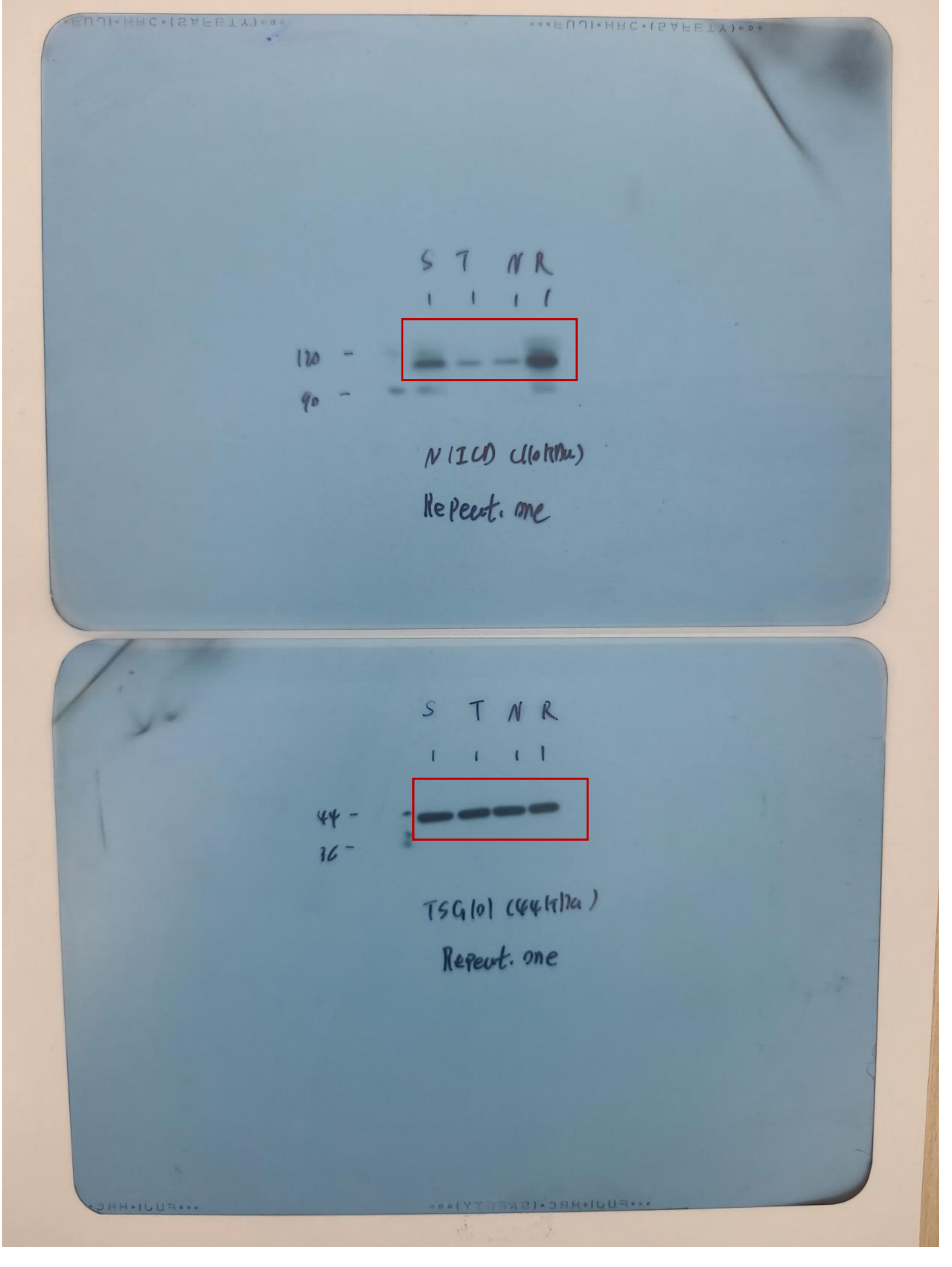

Full unedited gel for Figure 9E

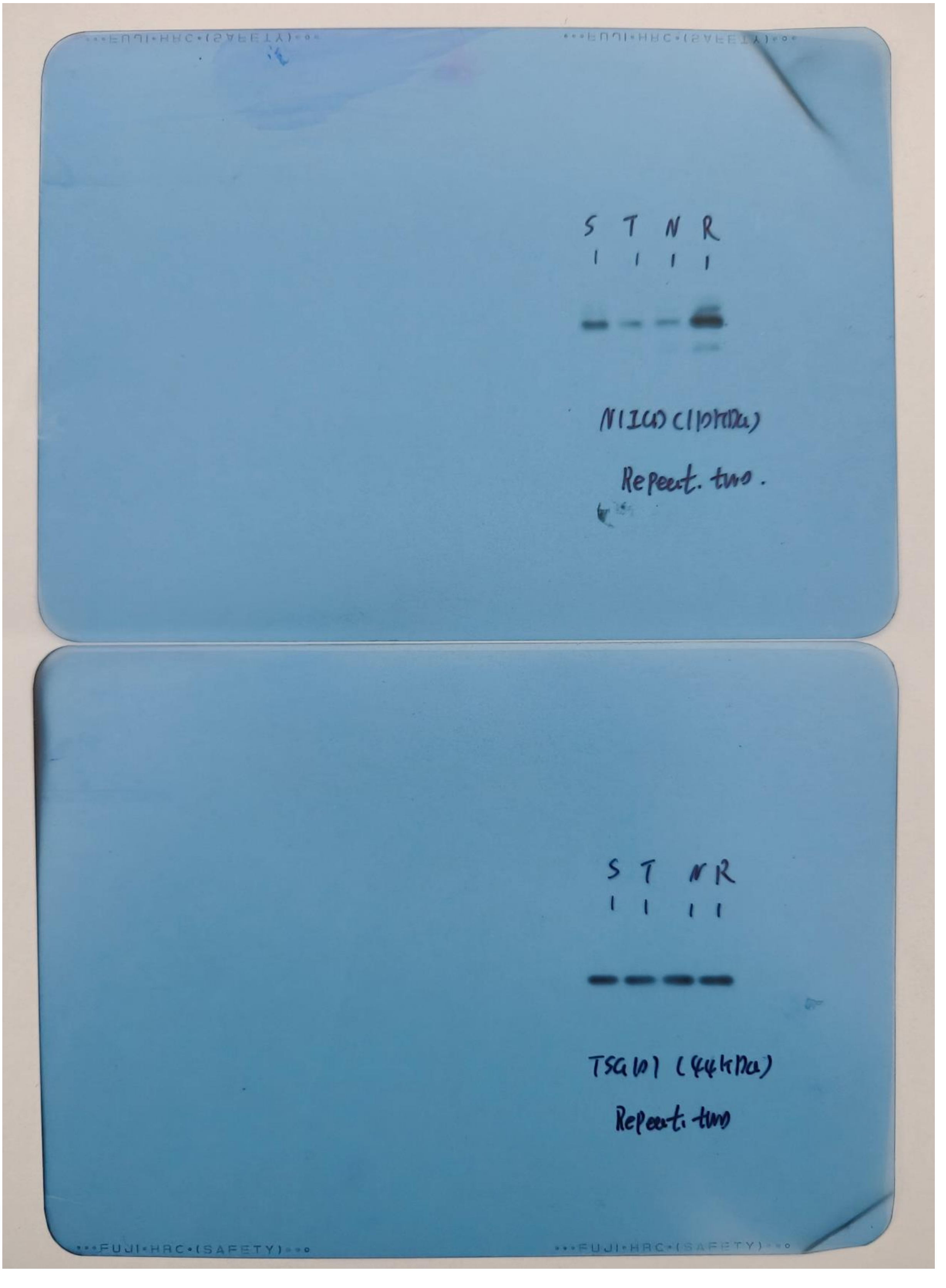

Full unedited gel for Figure 9E

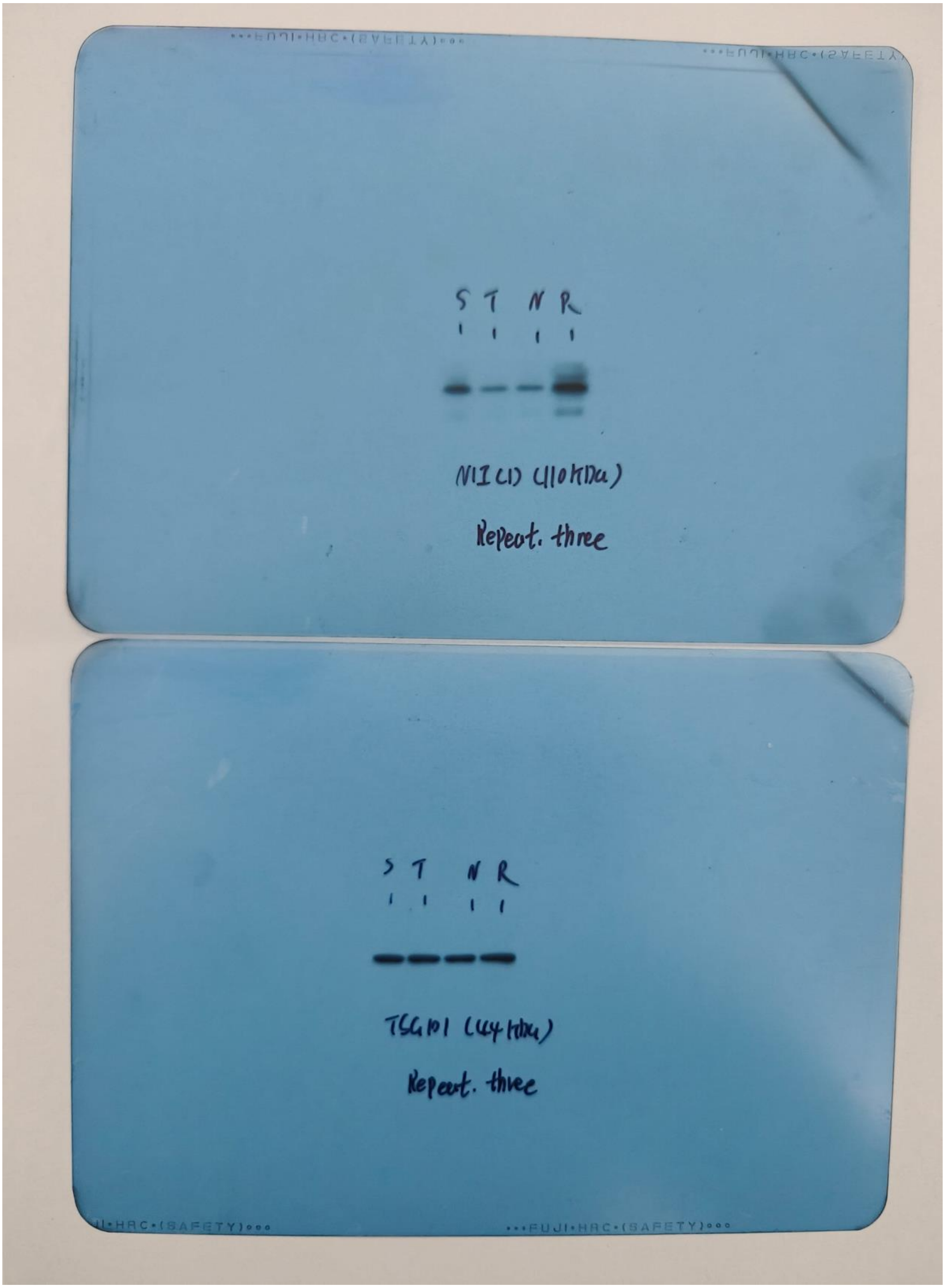

Full unedited gel for Figure 9F

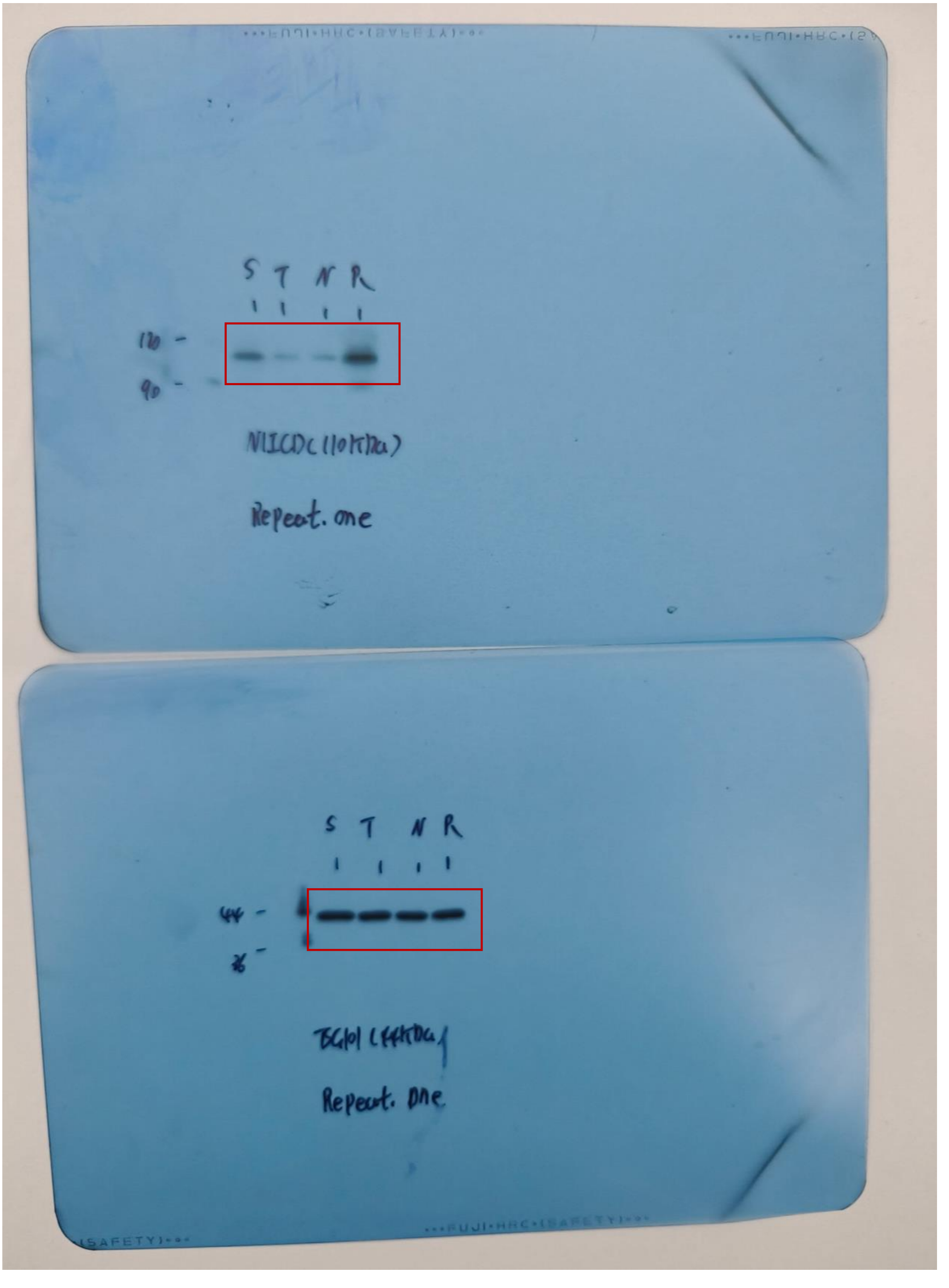

Full unedited gel for Figure 9F

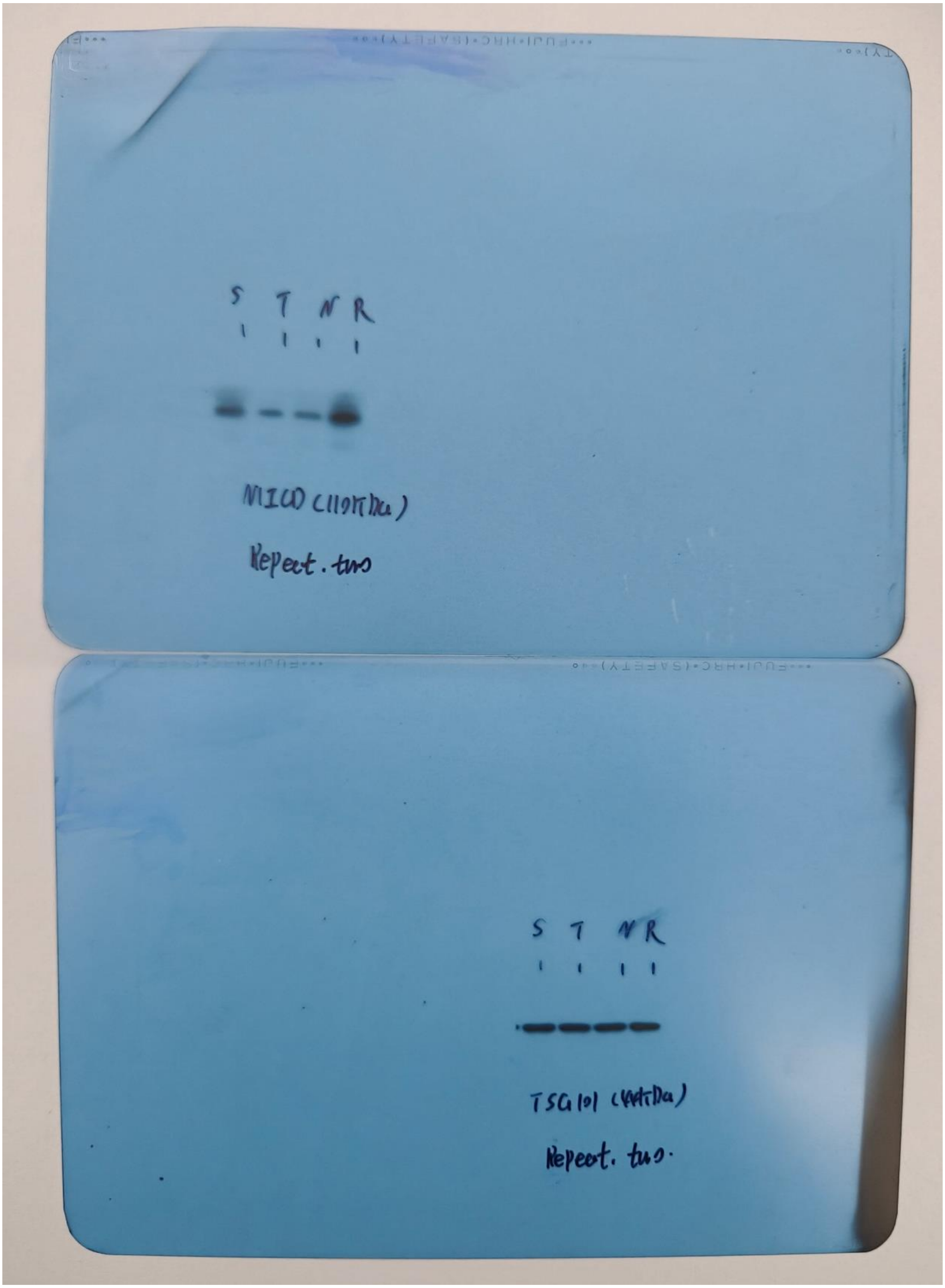

Full unedited gel for Figure 9F

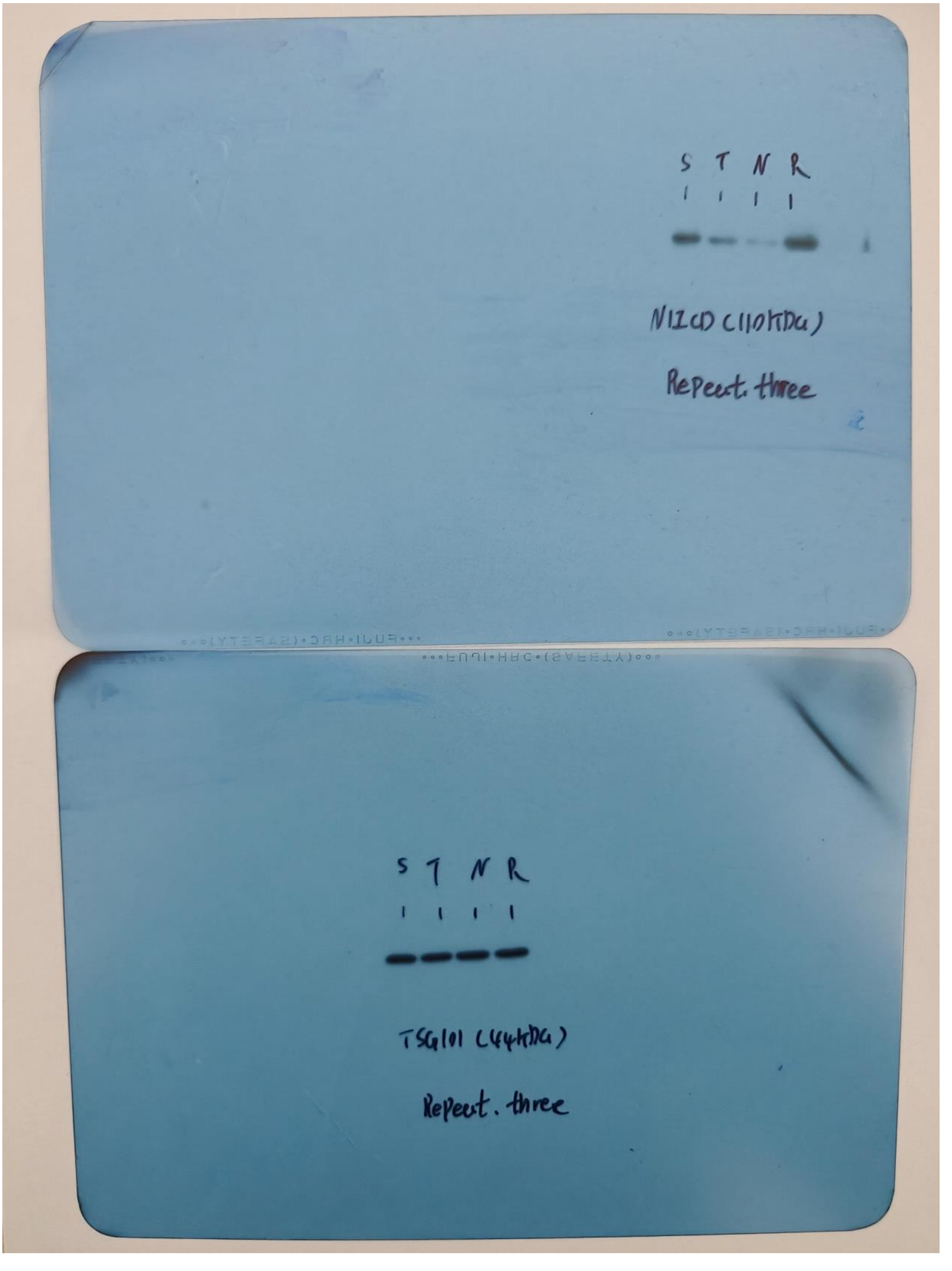

Full unedited gel for Figure S1A

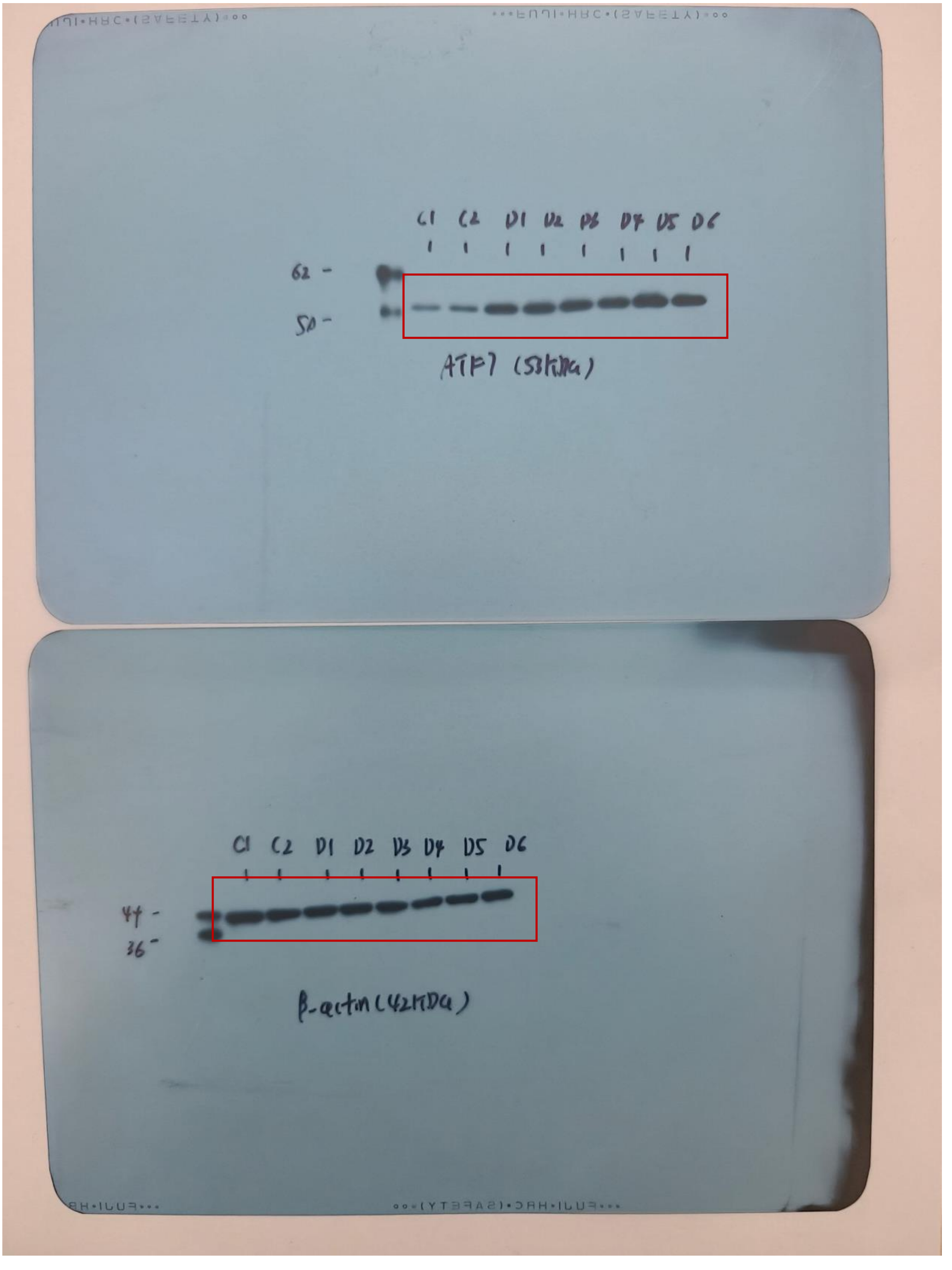

Full unedited gel for Figure S1A

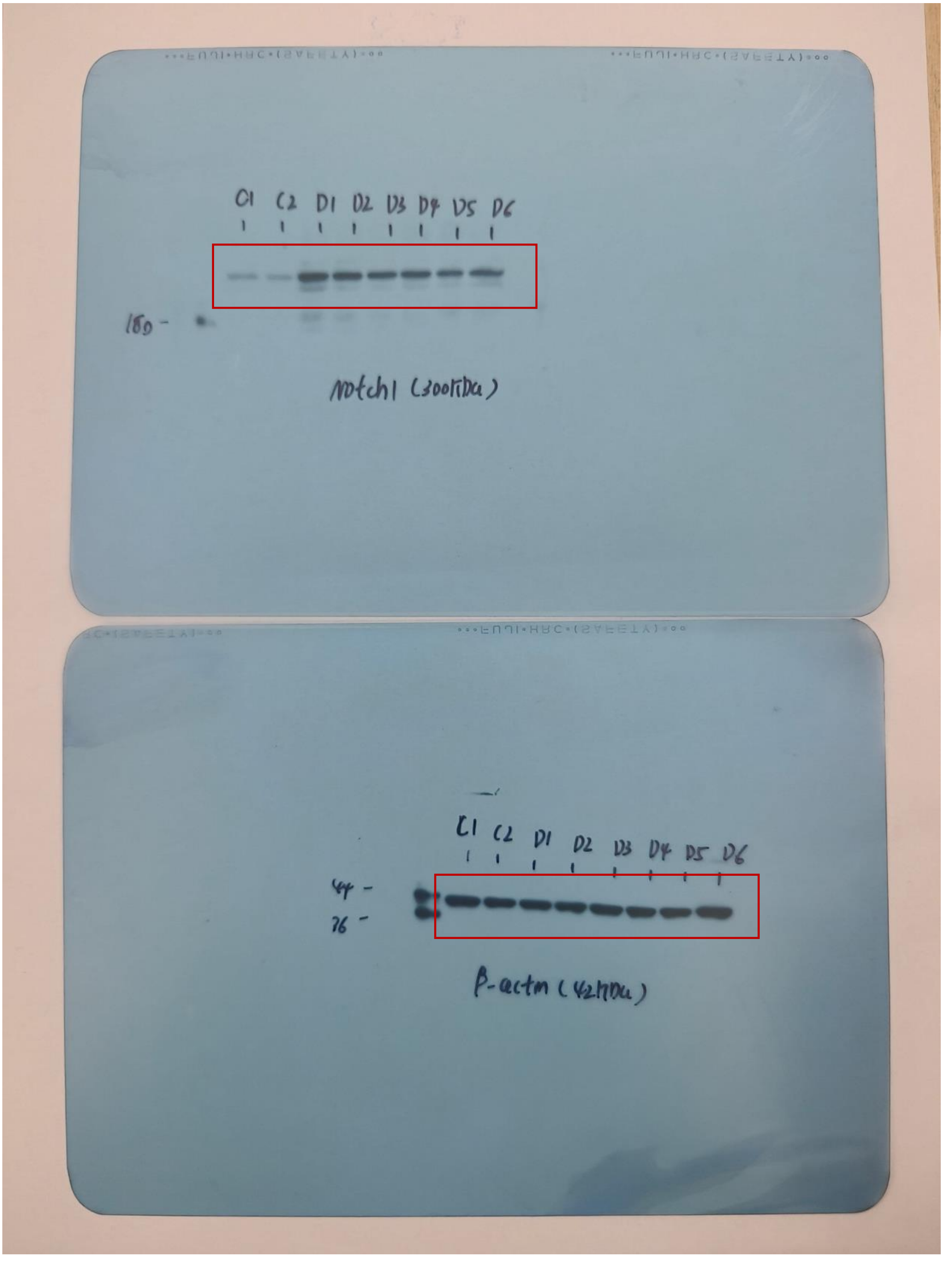

Full unedited gel for Figure S1B

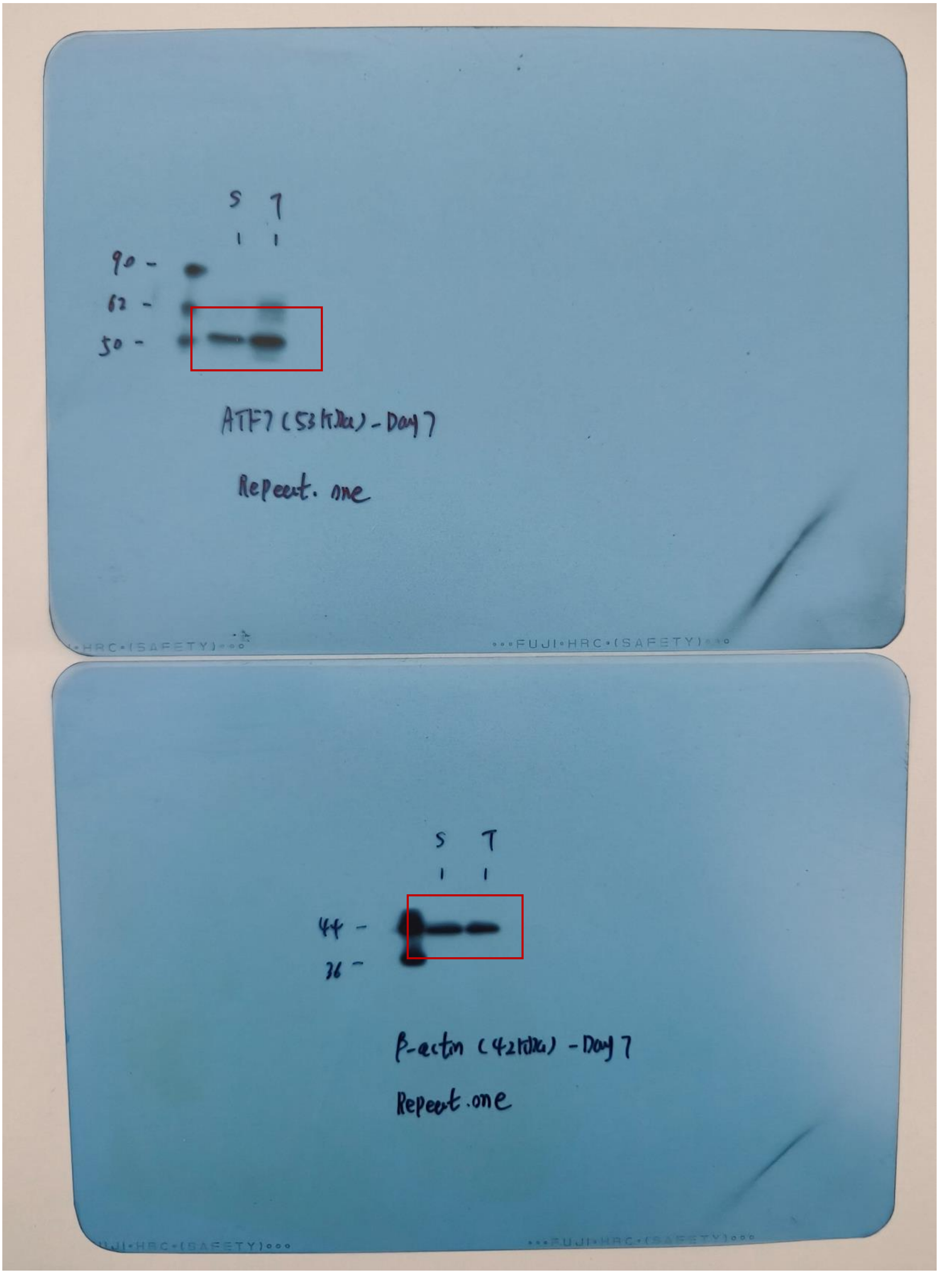

Full unedited gel for Figure S1B

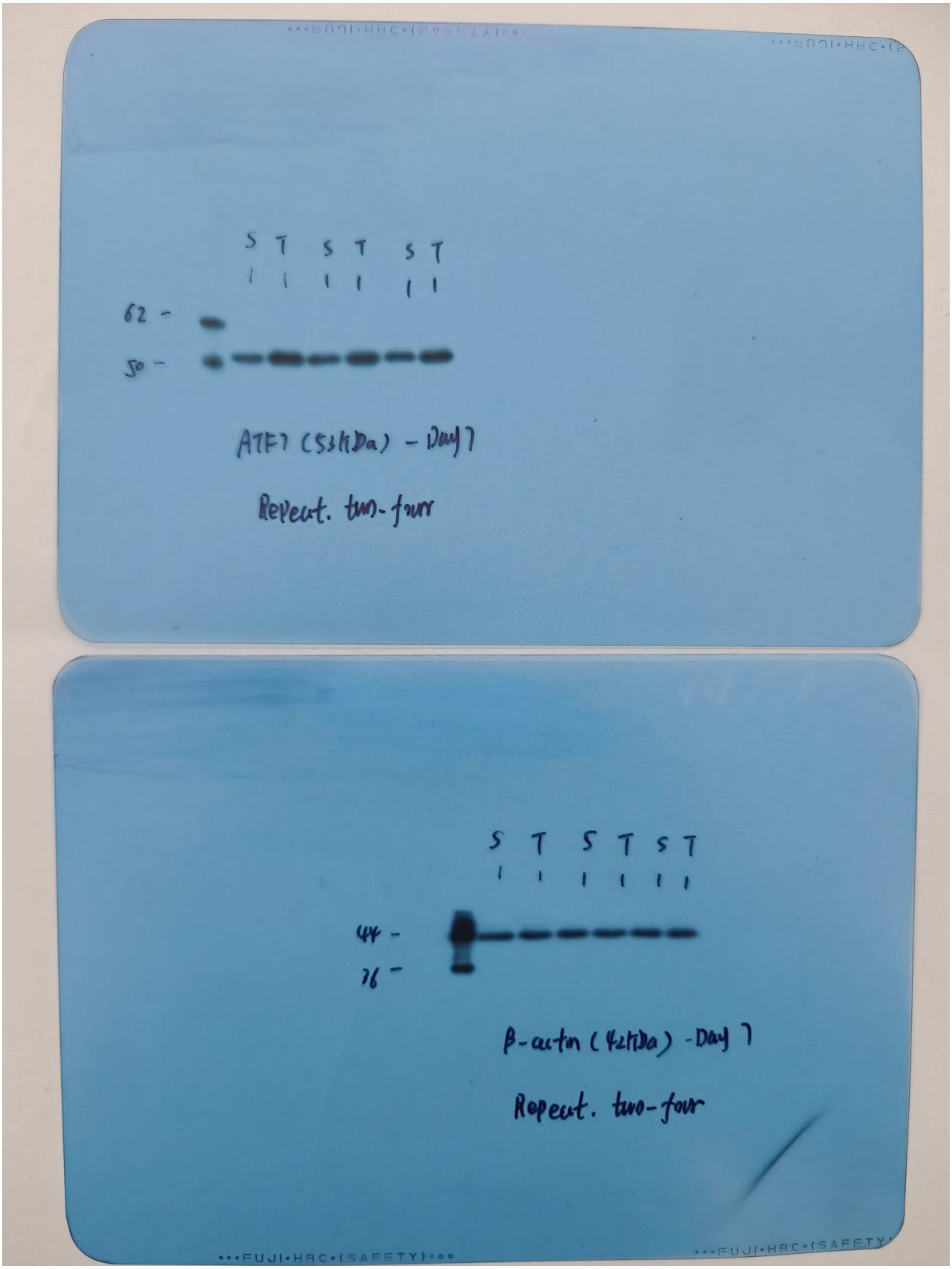

Full unedited gel for Figure S1B

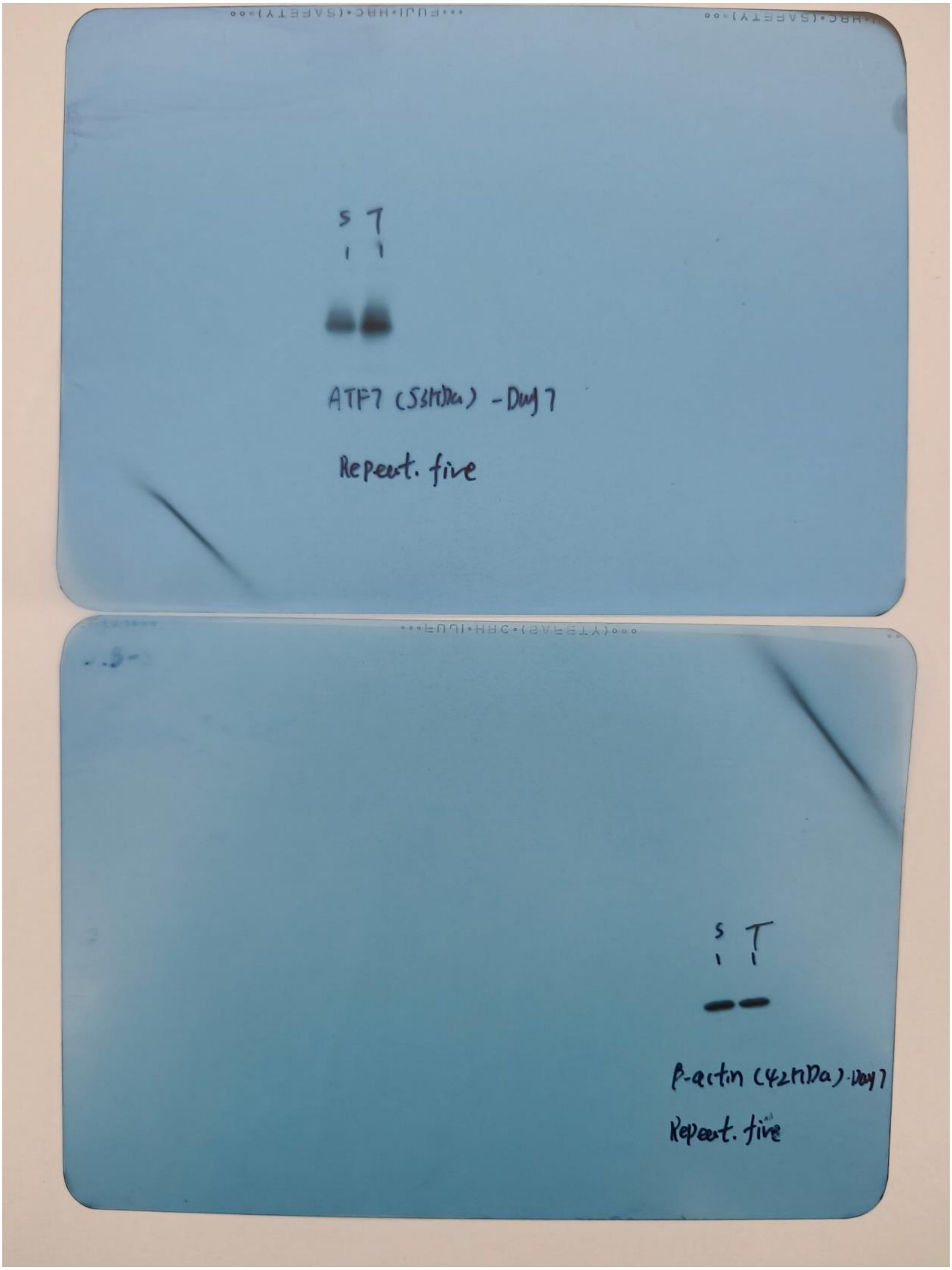

Full unedited gel for Figure S1B

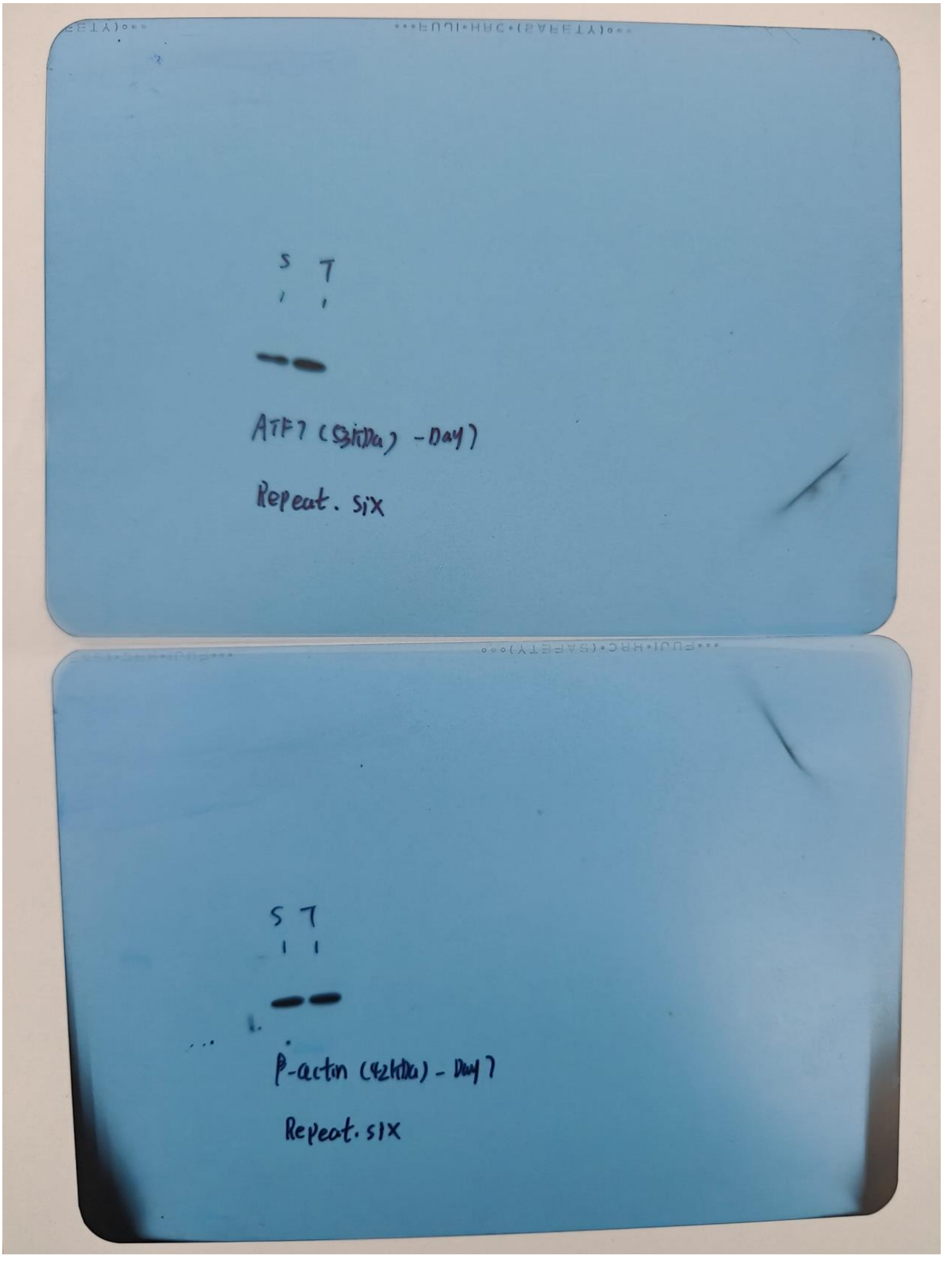

Full unedited gel for Figure S1B

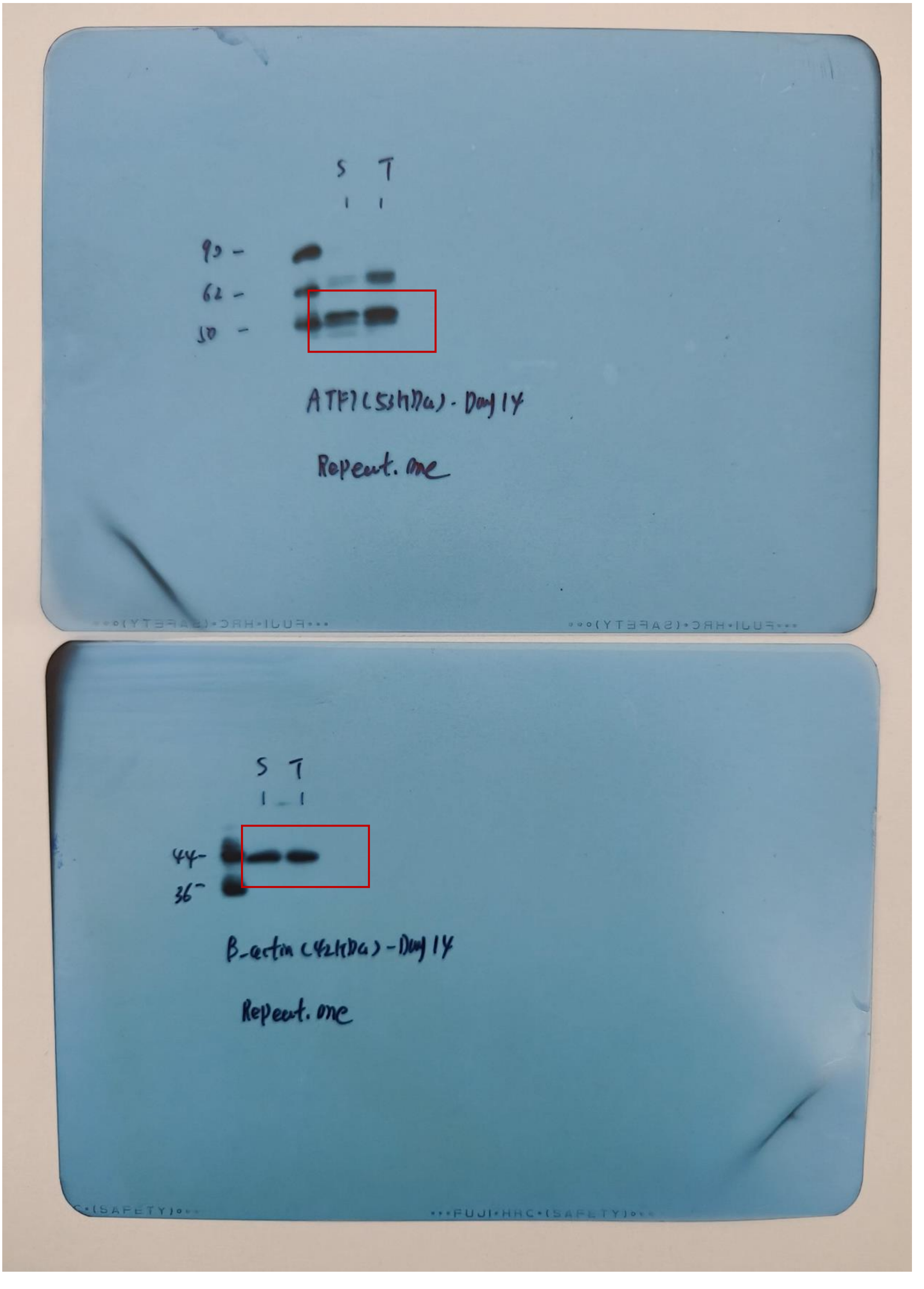

Full unedited gel for Figure S1B

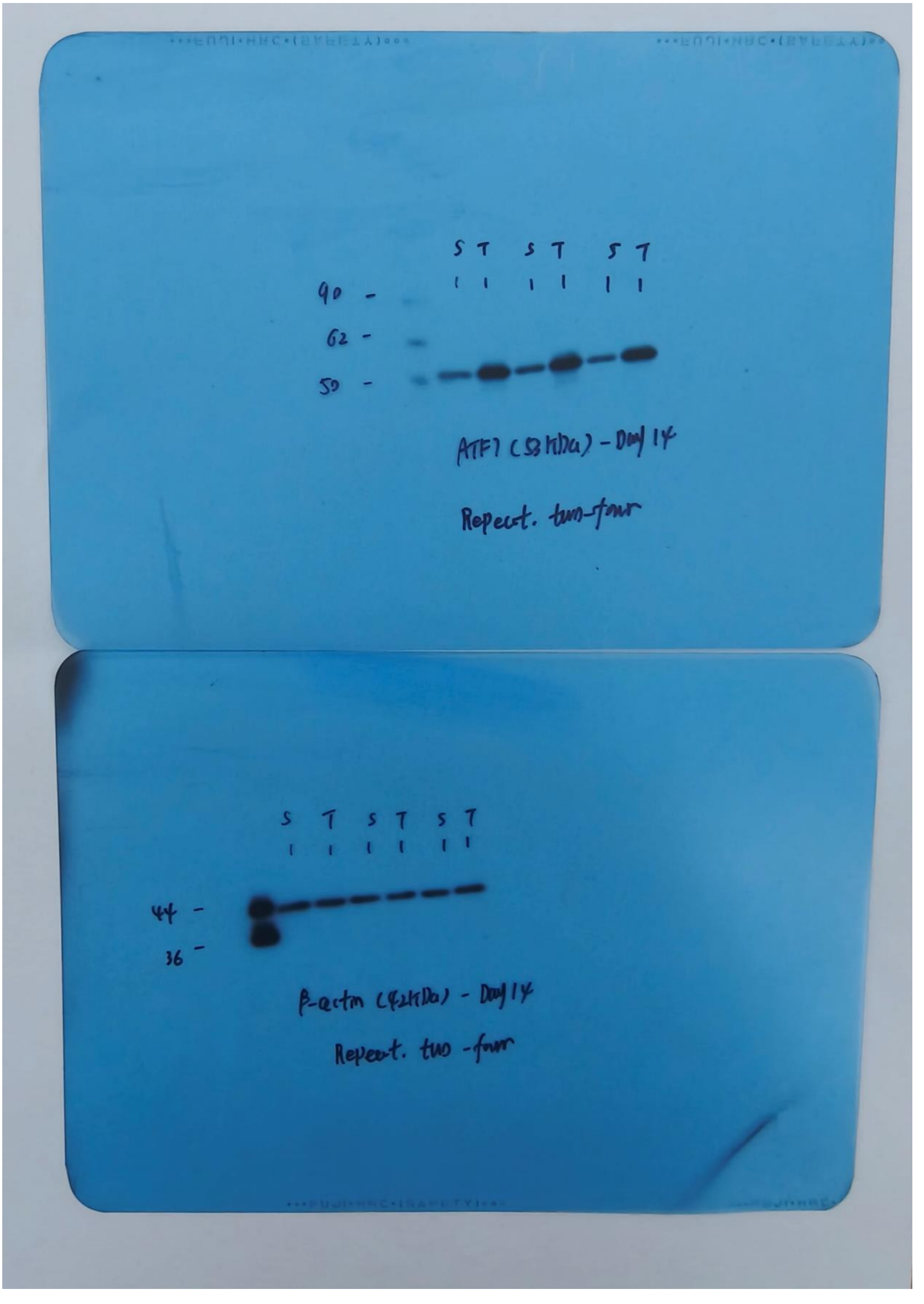

Full unedited gel for Figure S1B

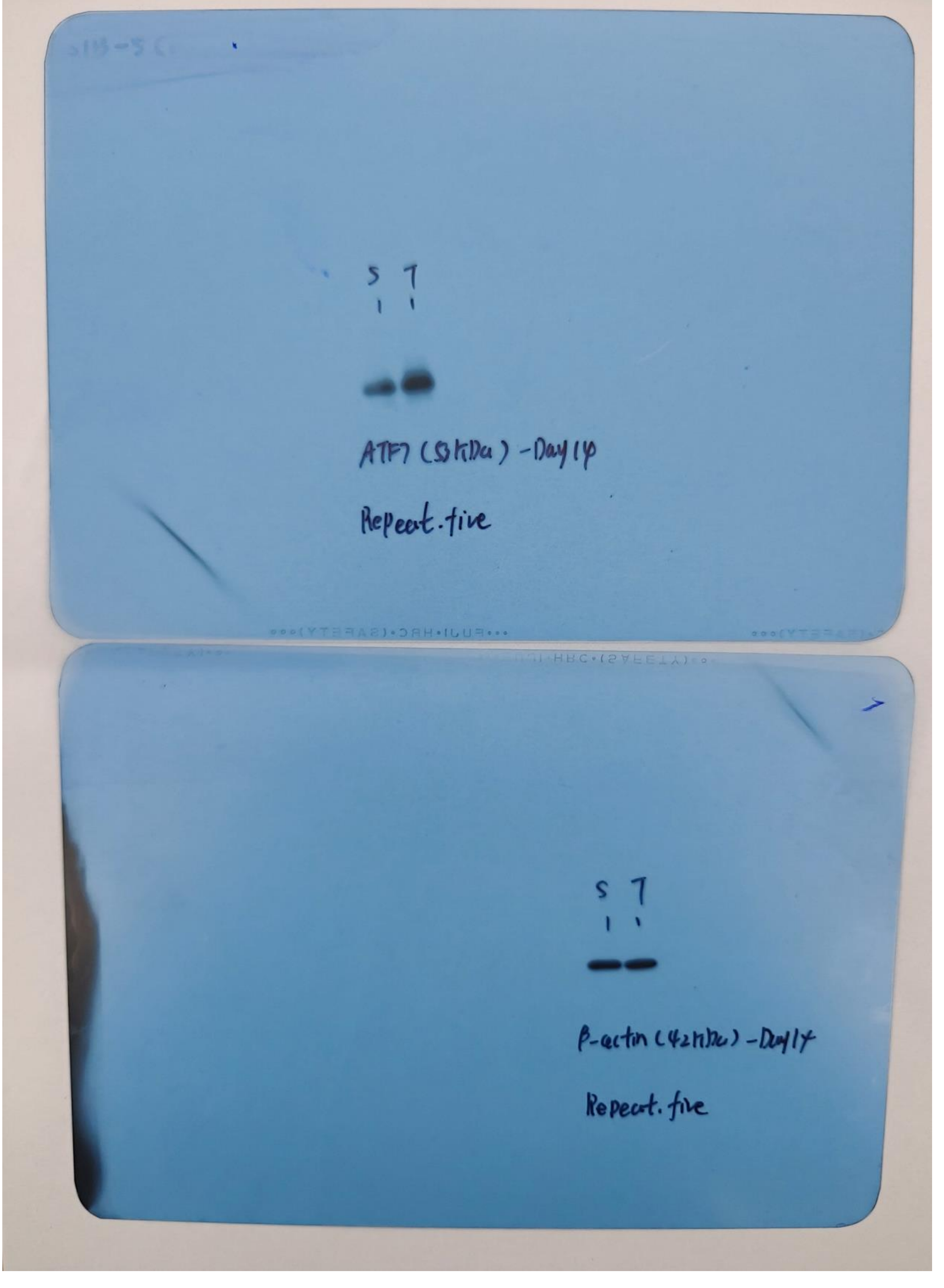

Full unedited gel for Figure S1B

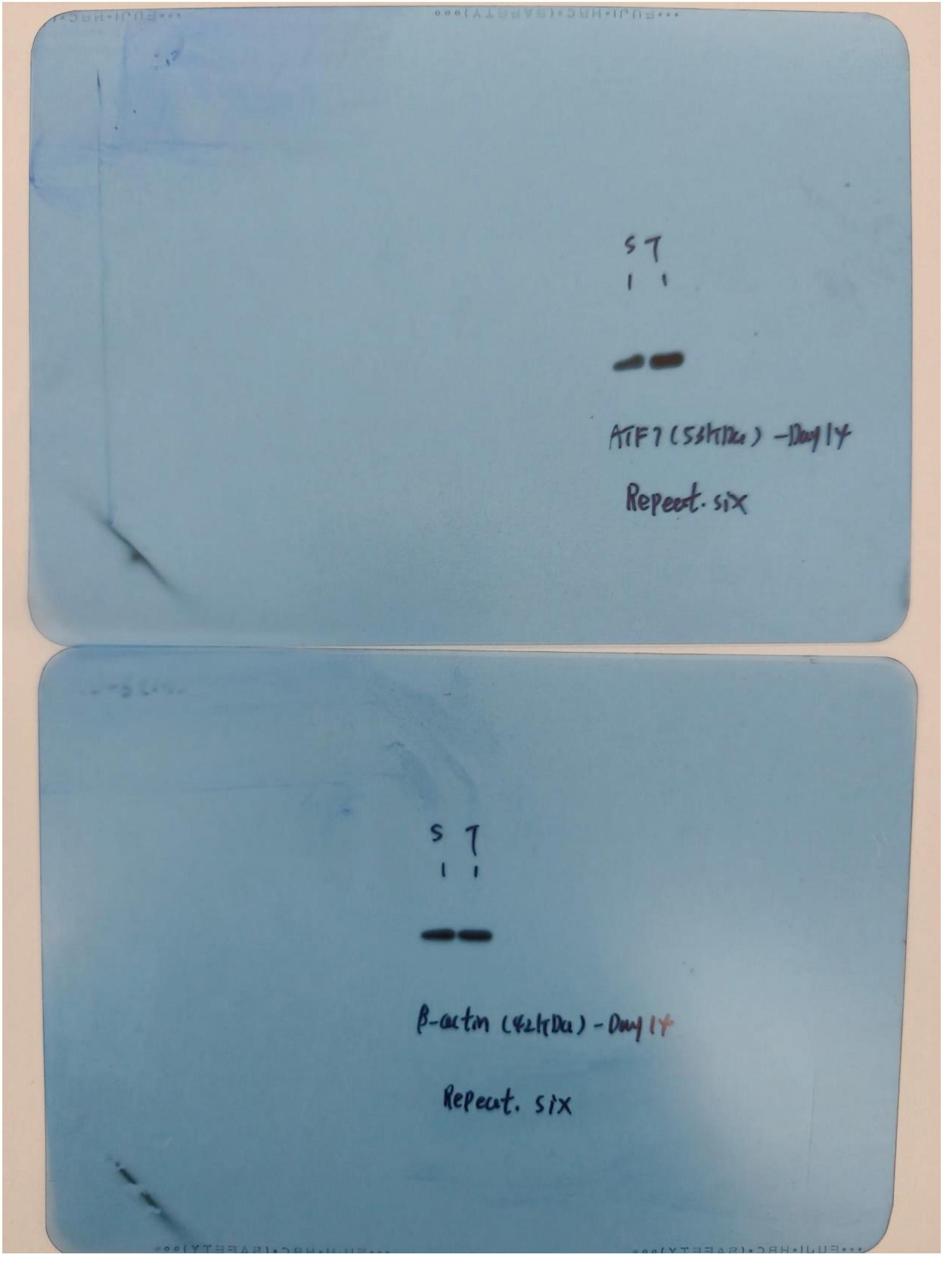

Full unedited gel for Figure S1C

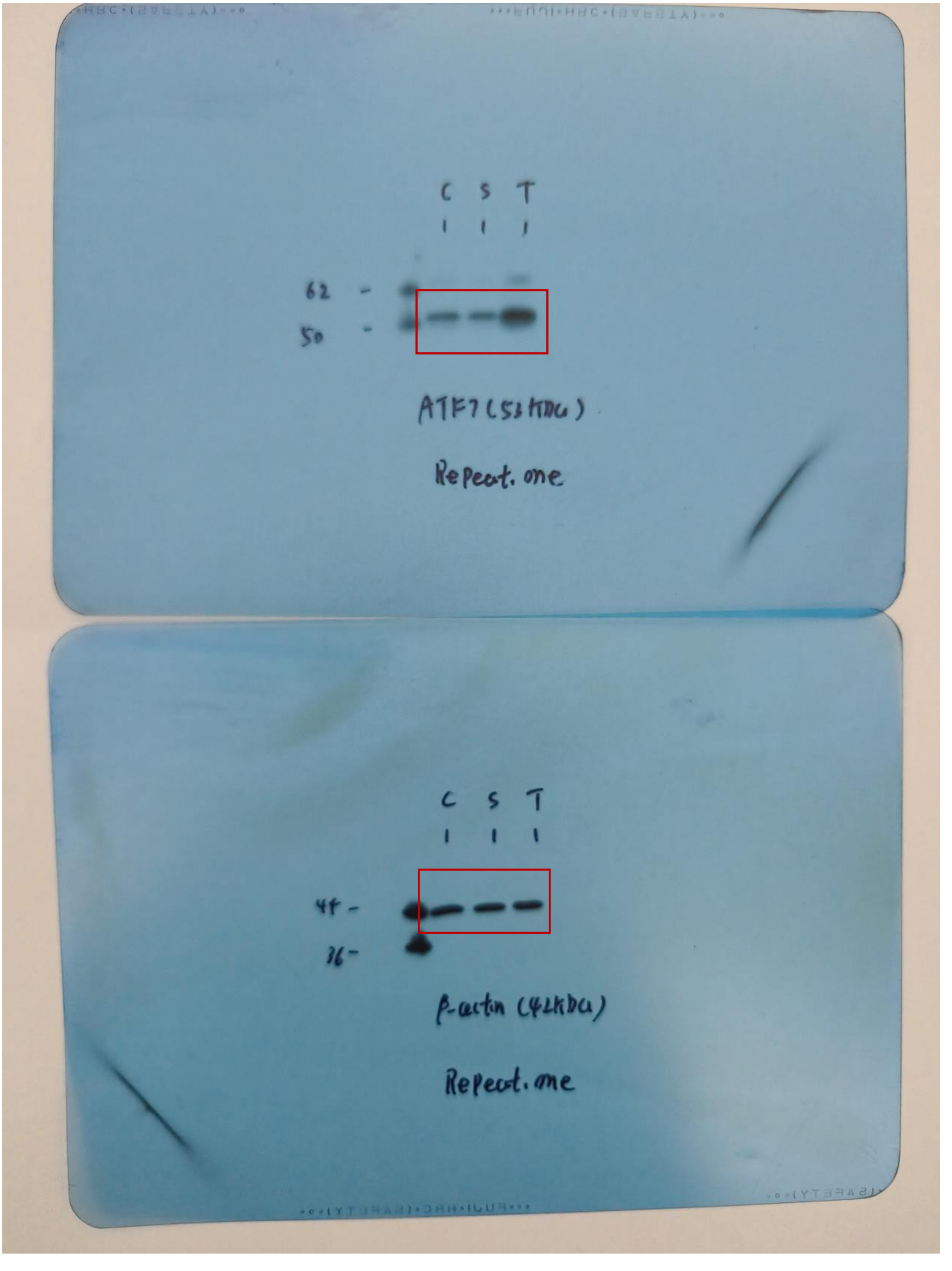

Full unedited gel for Figure S1C

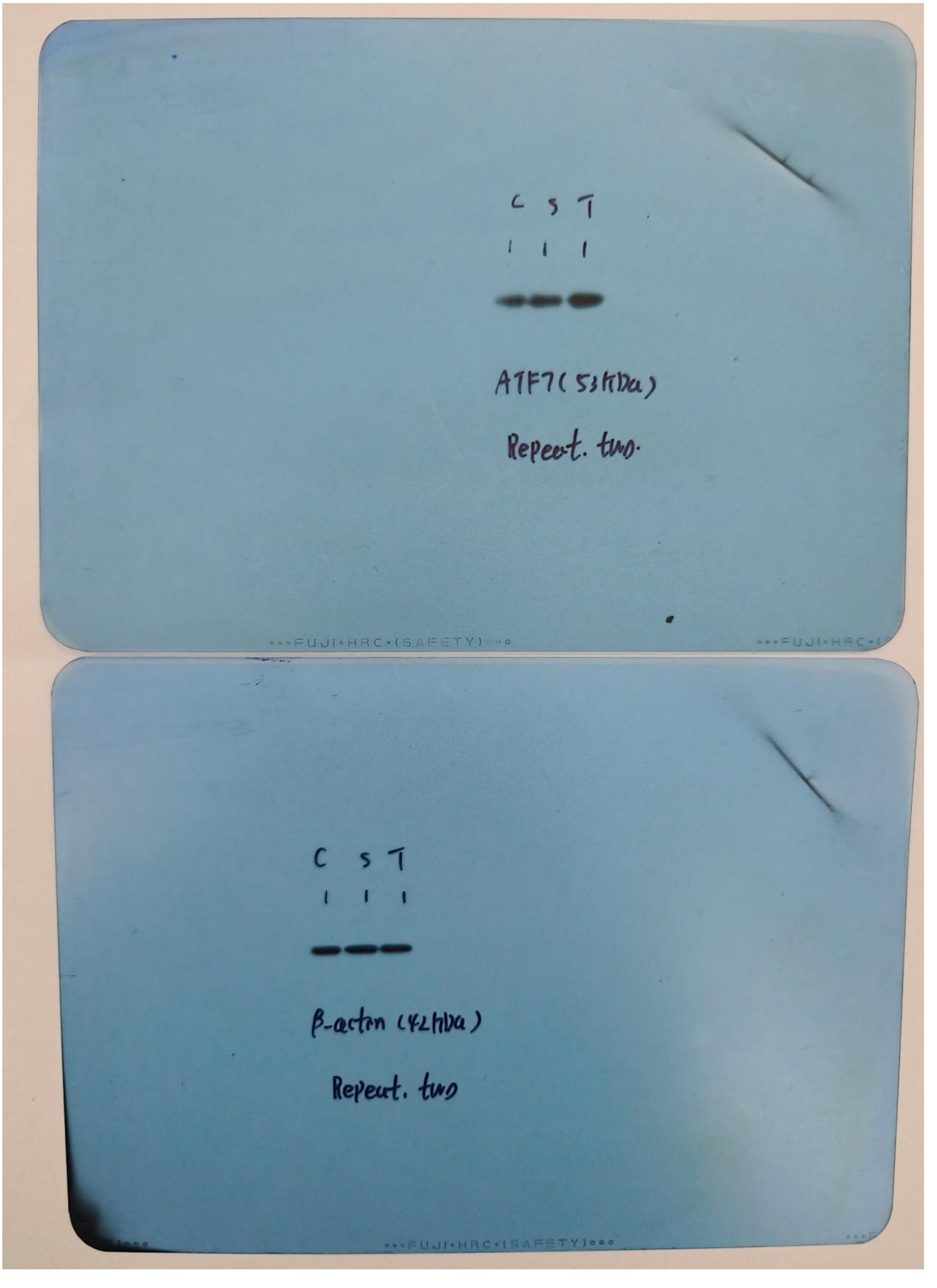

Full unedited gel for Figure S1C

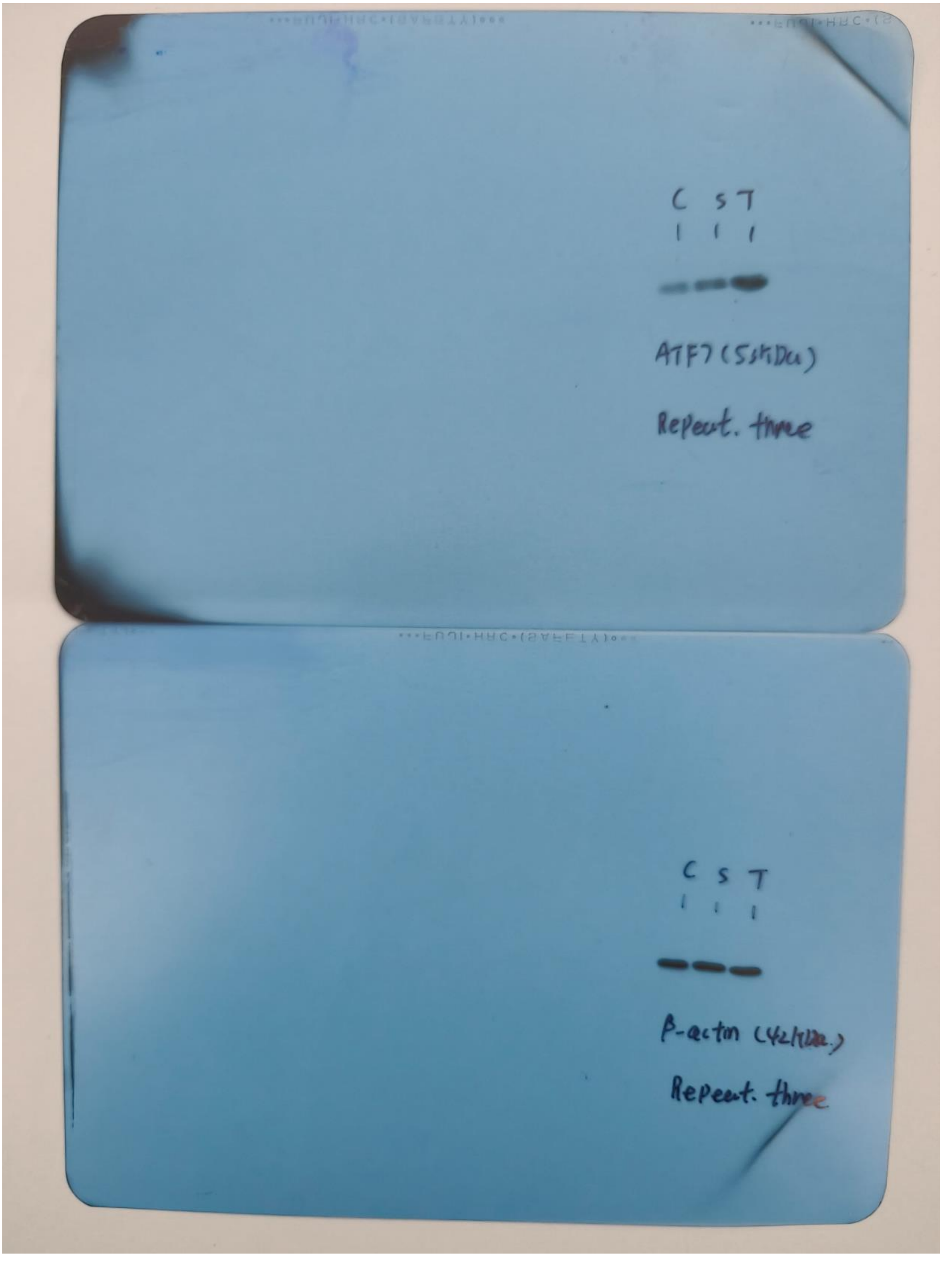

Full unedited gel for Figure S1D

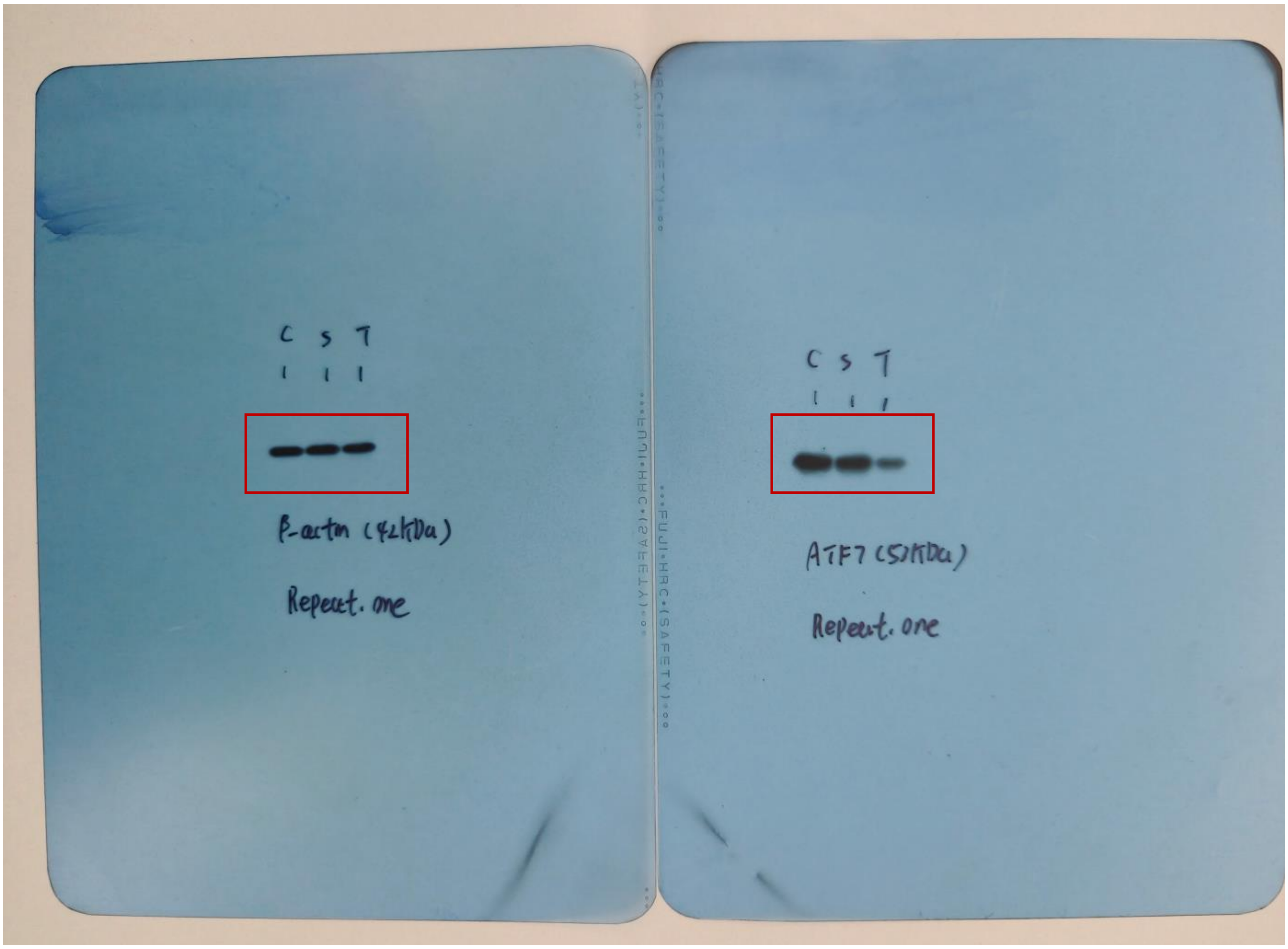

Full unedited gel for Figure S1D

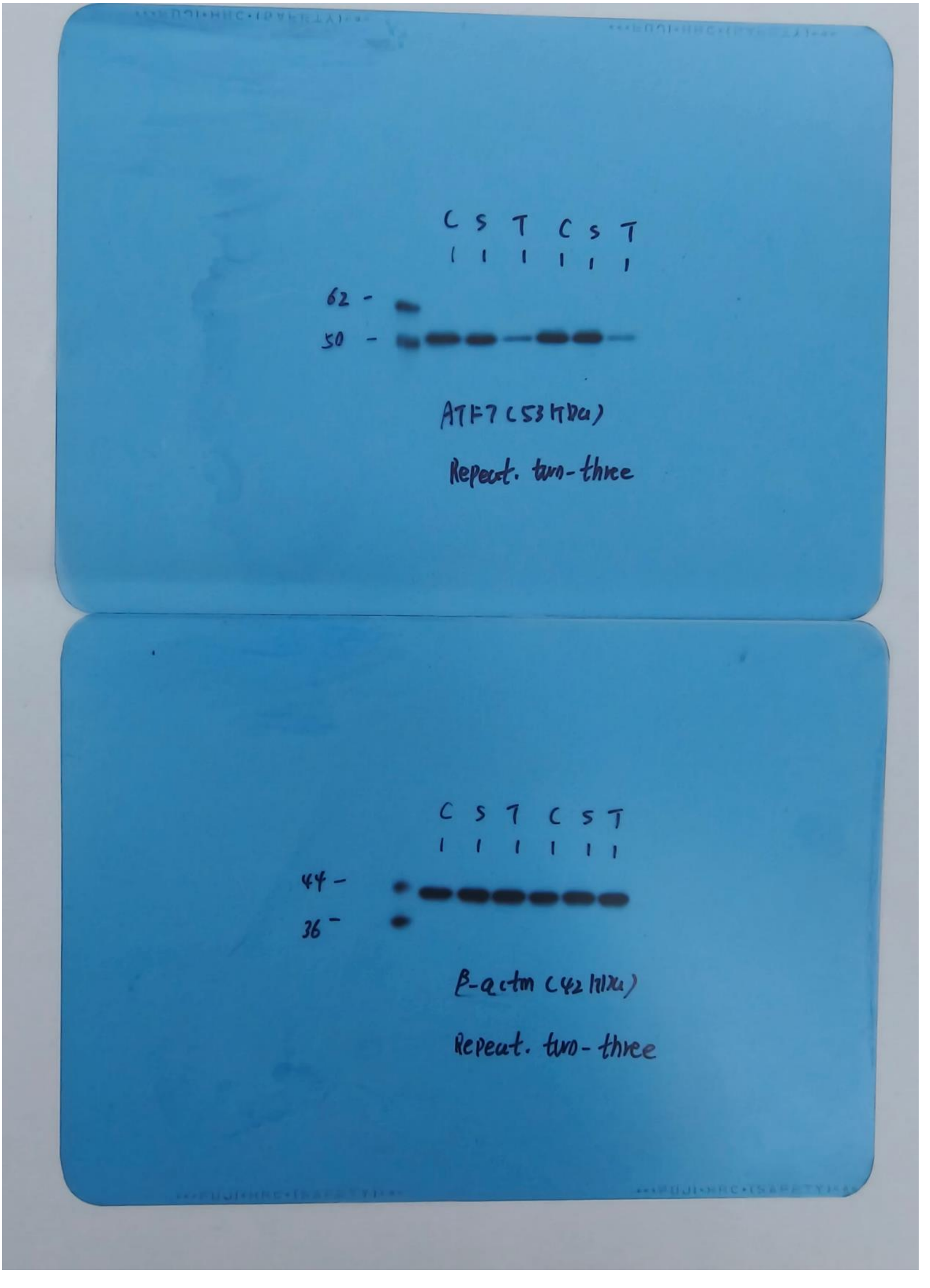

Full unedited gel for Figure S1E

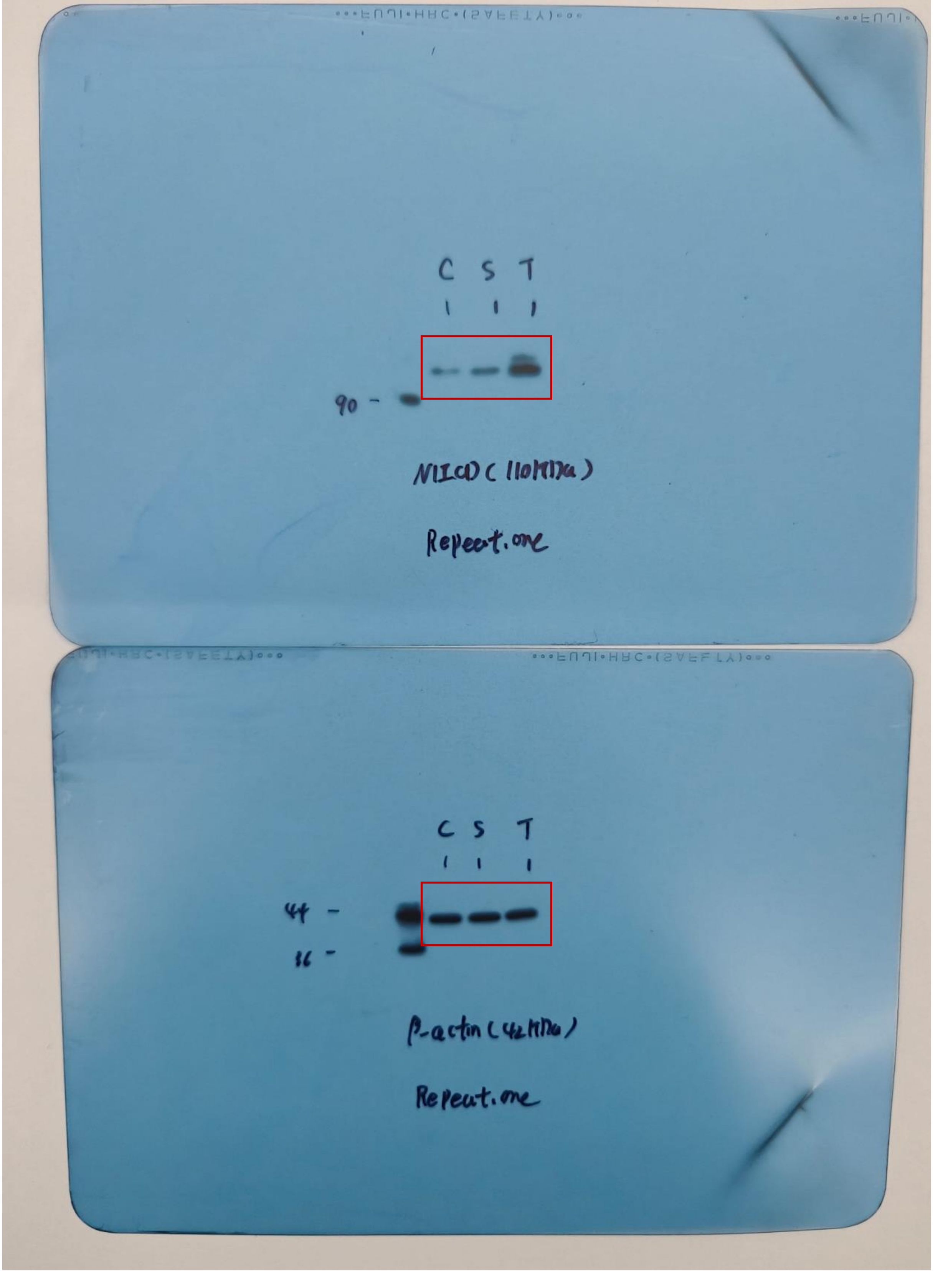

Full unedited gel for Figure S1E

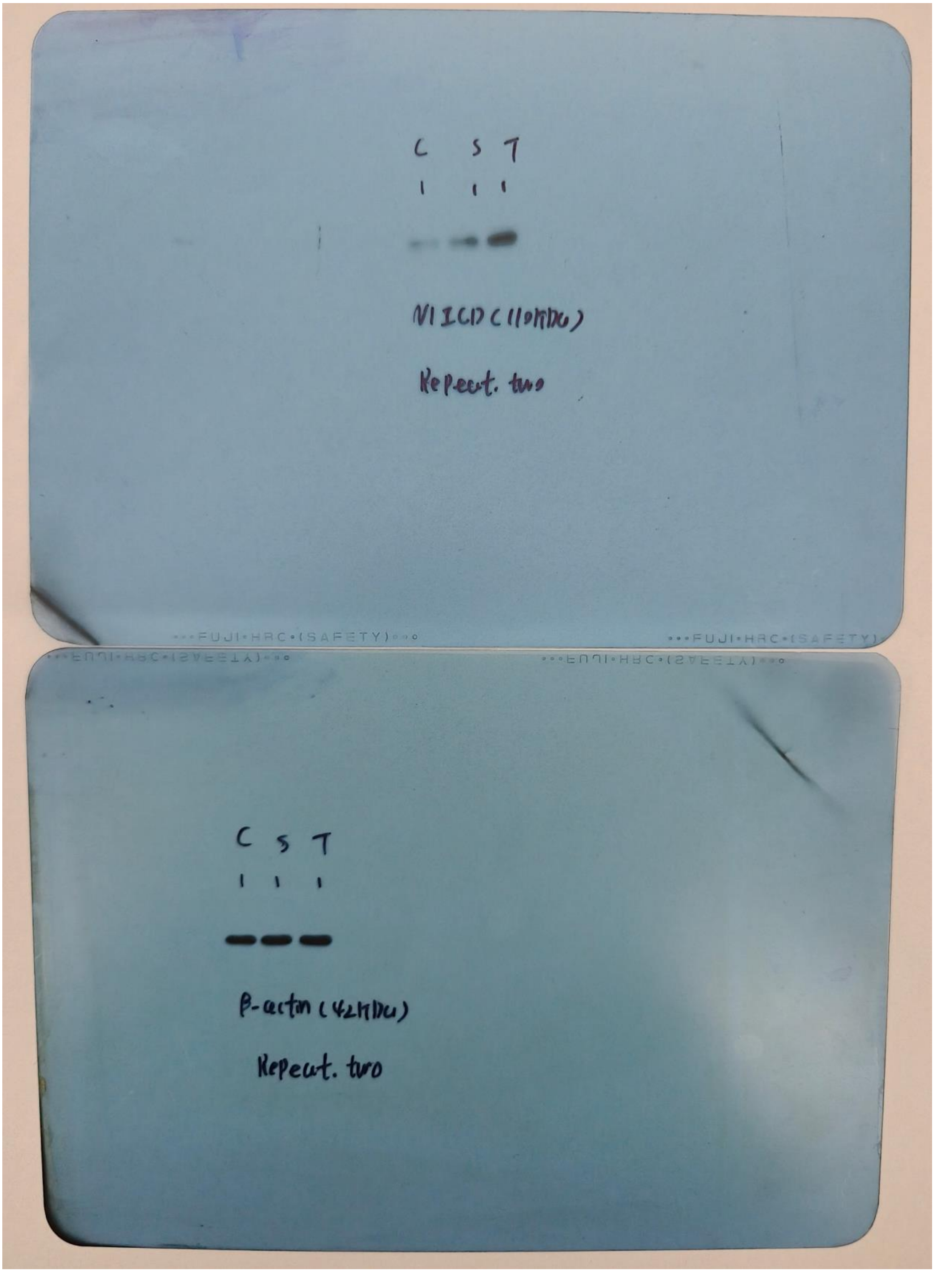

Full unedited gel for Figure S1E

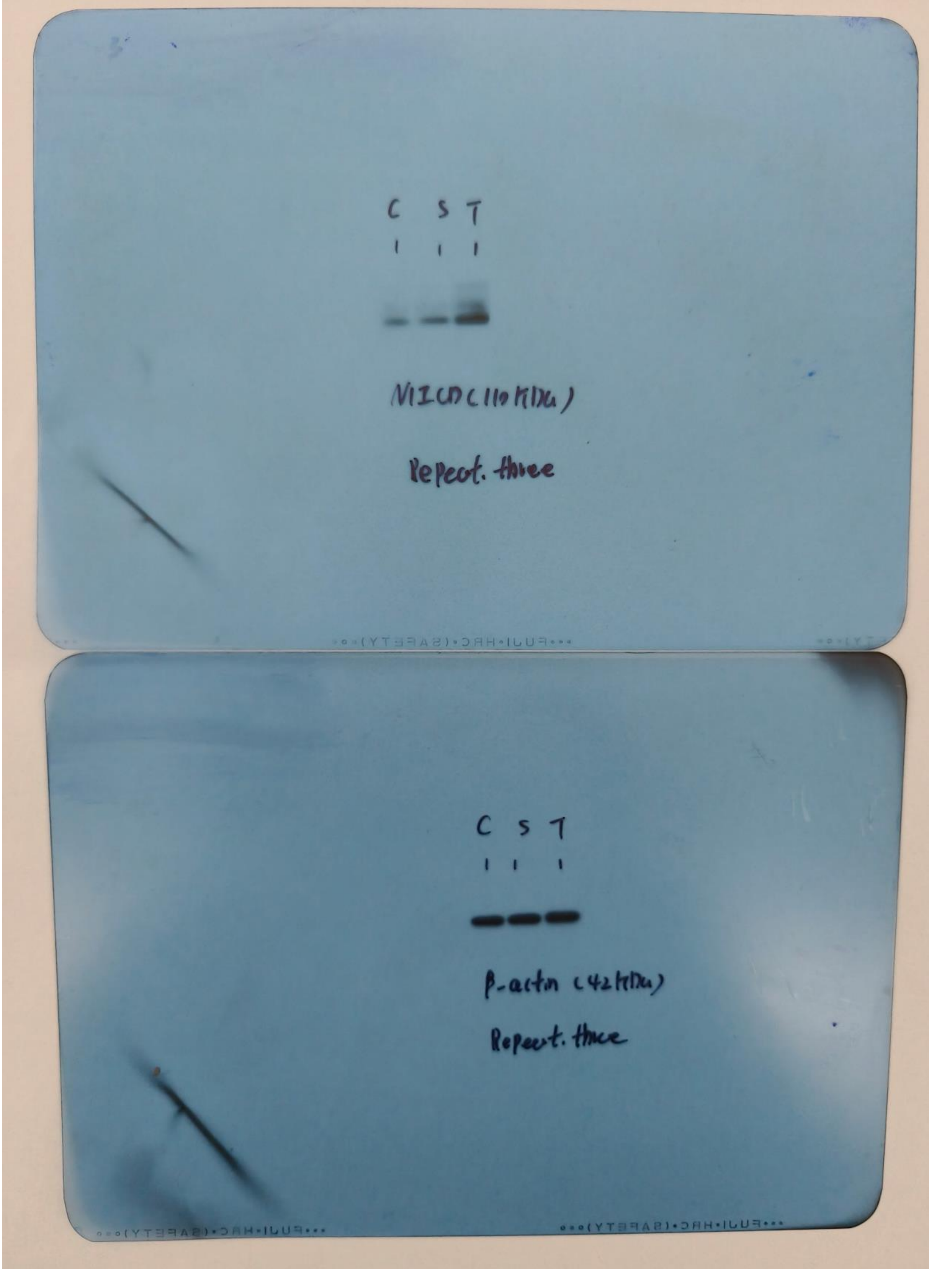

Supplement: Supplementary file 2 [file jciinsight-11-201178-s291.pdf]
